# Into the Cauldron of the Variant Soup: Insights into the Molecular Epidemiology and Transition to Endemicity of SARS-CoV-2 in Cyprus (November 2022–February 2024)

**DOI:** 10.3390/v16111686

**Published:** 2024-10-29

**Authors:** Andreas C. Chrysostomou, The COMESSAR Network, Leondios G. Kostrikis

**Affiliations:** 1 Department of Biological Sciences, University of Cyprus, Aglantzia, 2109 Nicosia, Cyprus; 2 Cyprus Academy of Sciences, Letters, and Arts, 60-68 Phaneromenis Street, 1011 Nicosia, Cyprus

**Keywords:** SARS-CoV-2, COVID-19, epidemic, molecular epidemiology, Cyprus

## Abstract

The coronavirus disease 2019 (COVID-19) pandemic, driven by the emergence of severe acute respiratory syndrome coronavirus 2 (SARS-CoV-2), has been characterized by the virus’s ongoing evolution, leading to the appearance of more transmissible variants that have often triggered infection surges. In this study, we analyzed the SARS-CoV-2 epidemic in Cyprus, utilizing 1627 viral sequences from infected individuals between November 2022 and February 2024. Over this period, 251 distinct lineages and sublineages were identified, predominantly categorized into three groups: Omicron 5, XBB, and JN.1 (parental lineage BA.2.86), all of which harbor S protein mutations linked to enhanced transmissibility and immune escape. Despite the relatively low numbers of new infections during this period, and the lack of any major waves, unlike earlier phases of the pandemic, these lineages demonstrated varying periods of dominance, with Omicron 5 prevailing from November 2022 to February 2023, XBB variants leading from March to November 2023, and JN.1 generating a wavelet from December 2023 to February 2024. These findings suggest that the SARS-CoV-2 epidemic in Cyprus has reached endemicity, with new variants gradually replacing previously circulating variants irrespective of seasonal patterns. This study highlights the critical importance of ongoing surveillance of SARS-CoV-2 evolution in Cyprus and emphasizes the role of preventive measures in limiting virus transmission, providing valuable insights for safeguarding public health.

## 1. Introduction

Severe acute respiratory syndrome coronavirus 2 (SARS-CoV-2) first appeared in China during December 2019 and swiftly spread worldwide, leading to the coronavirus disease 2019 (COVID-19) pandemic [1,2]. Over time, COVID-19 caused significant health and socioeconomic consequences, both globally and locally, including in Cyprus, which is the focal point of this investigation [3,4]. The impact of SARS-CoV-2 on humanity has been accentuated by the emergence of high-risk variants during the pandemic, which possess mutations that enable the virus to evade the immune system, make it more transmissible, and make it more virulent, often leading to substantial surges of new SARS-CoV-2 infections [5].

The appearance of new high-risk variants often result in massive outbreaks and new waves of SARS-CoV-2 infections and have been meticulously characterized from the beginning of the pandemic [5,6,7]. In fact, five variants of SARS-CoV-2 stood out throughout the course of COVID-19, and upon their emergence, they largely displaced other, less infectious circulating variants. Thus, they were termed as variants of concern (VOCs) by the World Health Organization (WHO), and this term included Alpha (B.1.1.7), Beta (B.1.351), Gamma (P.1), Delta (B.1.617.2), and Omicron (B.1.1.529) [7,8].

Since the emergence of Omicron during November 2021, it has defined the SARS-CoV-2 landscape, prevailing over all other variants [9]. Moreover, Omicron has proven to be highly polymorphic, and was first divided into different groups denoted as BA.1, BA.2, BA.3 (limited transmissibility), BA.4, and BA.5 (or Omicrons 1–5), characterized by a plethora of sublineages between the different groups [8,9,10]. The initial outbreak of Omicron was underlined by Omicron 1 (BA.1), followed by the dominance of Omicron 2 (BA.2), which was succeeded by Omicron 4 (BA.4) and 5 (BA.5), with the latter becoming more widespread compared to Omicron 4 [8,9,11]. Omicron 5’s reign was succeeded by numerous other Omicron 5 sublineages, as well as Omicron 2 sublineages, including “second-generation” BA.2 sublineages and recombinants [12,13]. This new era of SARS-CoV-2 was characterized by different sublineages convergently evolving to encompass key spike mutations, such as R346, K444, L452, L455, N460, F486, and P681. Sublineages such as BQ.*, BF.*, BA.2.75*, BA.2.12.1*, XBB*, BA.2.86*, and its progeny JN.1 (* refers to the inclusion of sublineages) formed a complex diversity of viral variants circulating around the world termed as “variant soup” [12,13,14,15,16,17,18]. The emergence of these variants was accompanied by a surge of new, albeit generally milder, infections, which had not reached the infection levels of their predecessors (such as Omicron 1 and 2) [19,20,21]. Thus, this new era of smaller waves was characterized by a hodgepodge of similar variants, none prevailing at a high enough degree to dominate as much as the previous VOCs [17,22,23]. In fact, the WHO had also announced that the emergency phase of COVID-19 ended as of 5 May 2023 [24].

The events culminating in this stage of the COVID-19 pandemic showcase the dynamic changes it has experienced, and it was closely researched both on an international and a local level, thanks to fields of study such as molecular epidemiology. In Cyprus, the study of the SARS-CoV-2 infection has served as a paradigm of the pandemic’s impact, with continuous and uninterrupted diachronic analyses, since the virus was identified on the island in the beginning of 2020. Specifically, from April 2020 to October 2022, the meticulous study of the SARS-CoV-2 infection in Cyprus has revealed that, during this period, there have been six major waves of SARS-CoV-2 infection [25,26,27]. The inaugural wave was typified by the B.1.1 lineage, particularly the B.1.1.29 sublineage, from April to June 2020. The subsequent wave, spanning from September 2020 to January 2021, was primarily marked by the B.1.258 lineage and its associated sublineages. The third wave, spanning February to May 2021, was characterized by the Alpha variant and its sublineages. Subsequently, the fourth wave, extending from June to September 2021, was predominantly shaped by the Delta variant and its corresponding sublineages. The fifth wave, spanning from November 2021 to May 2022, was marked by the appearance of Omicron and had the largest number of new infections. This wave was split into two peaks, with the first one being characterized by Omicron 1 (January 2022) and the second peak by Omicron 2 (March 2022). The sixth and final major wave took place from late May 2022 to August 2022, with Omicron 5 being the predominant variant during that period [25,26,27].

In this study, we investigated the impact of SARS-CoV-2 in Cyprus from November 2022 to February 2024, using genetic and molecular epidemiology analyses. Initially, this phase was dominated by sublineages of BA.5 and BA.2, including BQ., BF., BA.2.75*, and BA.2.12.1* [28]. This period also included Omicron 2 recombinants, classified as XBB* [29], which became dominant and remained as such until late 2023. Subsequently, BA.2 sublineages, specifically JN.1 (of the BA.2.86* group), succeeded the XBBs [18] during December 2023, briefly triggering a wavelet, with a minor surge in infections, that receded by February 2024. Thus, our analyses were aimed towards elucidating the circulating lineages and mutational landscape of Cyprus during the new study period, employing a genetic epidemiology approach to explore their dynamics. Unlike the previous periods (April 2020 to October 2022), there were no major waves of infection present, and no single variant had dominated to the degree and duration as in previous waves in Cyprus. Substantial progress has been made in managing SARS-CoV-2 and COVID-19, and as variants continue to evolve, vigilance remains paramount. This necessitates ongoing surveillance of variant circulation and flexible public health measures to adapt to the ever-changing landscape, ensuring continued public health security.

## 2. Materials and Methods

### 2.1. Collection of Samples, Extraction of RNA, Real-Time RT-PCR for SARS-CoV-2, and Next-Generation Sequencing (NGS)

The collection of samples, the extraction of RNA, and the real-time RT-PCR for SARS-CoV-2 were outlined in our previous published manuscripts [25,26,27]. In summary, medical facilities in Cyprus collected or received nasopharyngeal and/or oropharyngeal swab samples preserved in transport medium for diagnostic purposes. Subsequently, RNA extraction was conducted to identify SARS-CoV-2-positive samples through real-time RT-PCR. NGS was performed utilizing the COVIDSeq Assay (Illumina Inc., San Diego, CA, USA) and ARTIC V4.1 PCR primers (and updated versions such as V5.3.2). NGS was carried out by Medicover Genetics (formerly NIPD Genetics), as in our previously published studies [25,26,27].

### 2.2. Sequences Employed in This Research

The sequences employed in this prospective study were sourced for the molecular epidemiological surveillance study led by the Laboratory of Biotechnology and Molecular Virology at the University of Cyprus (BMV UCY). This study was conducted in collaboration and with the approval of the Cyprus Ministry of Health, as well as members of the Cypriot Comprehensive Molecular Epidemiological Study on SARS-CoV-2 (COMESSAR) network. As part of this joint initiative, BMV UCY acquired a total of 2439 whole-genome SARS-CoV-2 sequences from individuals infected in Cyprus between November 2022 and February 2024.

From the starting number of 2439 SARS-CoV-2 sequences, 812 sequences did not qualify based on the quality control protocol that was performed. Specifically, this first entailed the removal of one sequence with missing data and 35 sequences that were determined to be duplicates. The sequences were classified and underwent additional quality evaluation using the Nextclade webtool (Nextclade v.3.4.0) (https://clades.nextstrain.org/Nextclade, accessed on 7 May 2024) [30]. Through this step, 776 sequences of subpar quality were excluded. Consequently, the final list contained the 1627 sequences determined as “good quality” under the “qc.overallStatus” parameter, which were employed for the analyses performed in this study [30]. As it was outlined in our previous research, this detailed method was adopted to minimize possible misinterpretations arising from artifacts introduced during sequencing and assembly, covering concerns such as ambiguous nucleotides, data gaps, frameshifts, and premature stop codons [27,30].

Thus, the ensuing dataset consisted of 1627 near-whole-genome SARS-CoV-2 sequences that met the denoted quality control criteria and were then utilized for subsequent analyses. Furthermore, these meticulously curated sequences will be accessible through the GISAID database after the publication of the manuscript [31]. These 1627 sequences were derived from eight different medical facilities located in Cyprus that perform SARS-CoV-2 testing. Specifically, 723 sequences originated from Nicosia General Hospital, 320 from Limassol General Hospital, 311 from Ammochostos General Hospital, 139 from Medicover Genetics, 77 from Synlab Cyprus, 23 from Tymvios Medical Labs, 20 from S.C.I.N.A. Bioanalysis Sciomedical Centre Ltd. (Limassol, Cyprus), and 14 from Bioiatriki Healthcare Group/Yiannoukas Medical Laboratories Ltd. (Paphos, Cyprus).

The analysis of these sequences has been performed under the approval of the Cyprus National Bioethics Committee (EEBK 21.1.04.43.01). All sequences were double coded once received by BMV UCY in order to preserve anonymity. Furthermore, all sequences were handled in accordance with the guidelines and regulations denoted by the Cyprus National Bioethics Committee.

### 2.3. Bioinformatic Analysis

#### 2.3.1. Classification of Lineages and Mutation Calling

Lineage classification and mutation calling was performed using the Nextclade webtool (Nextclade v.3.4.0) (https://clades.nextstrain.org/Nextclade, accessed on 7 May 2024) [30].

#### 2.3.2. Phylogenetic Inference

This analysis was performed for the entirety of the dataset of 1627 near-whole-genome SARS-CoV-2 sequences. A multiple alignment was first performed using MAFFT v.7.505 [32], followed by visual inspection and manual editing through AliView v.1.26 [33]. The aligned dataset was then employed for the construction of a maximum likelihood (ML) tree using IQtree v.2.2 software [34]. The SH-like approximate likelihood ratio test (SH-aLRT) [35] and the ultrafast bootstrap (UFB) procedure [36] were used to validate the reliability of the branches in the generated tree.

### 2.4. Figure Details and Calculations

The data shown in “Results Section 3.1” were obtained from the Cyprus Ministry of Health, the Press and Information Office, and the KIOS Research and Innovation Center of Excellence (KIOS CoE), operating within the University of Cyprus. These data were then processed to ascertain the percent positivity [37,38,39], which was achieved by dividing positive SARS-CoV-2 cases per week by the weekly number of SARS-CoV-2 tests [39].

The aggregation of positive SARS-CoV-2 cases per month documented in Cyprus from March 2020 to February 2024 was proportionally linked with SARS-CoV-2 variants. Furthermore, the visual depictions of spike proteins on the colored virions underneath each lineage, shown in the following figures, were produced through PyMol (Version 2.4.1, Schrödinger, LLC, https://www.pymol.org, accessed on 18 February 2021). These illustrations were based on the Protein Data Bank entry 6XEY [40,41], along with other sources, to outline the spike protein domains [42,43,44,45,46,47,48,49,50].

## 3. Results

### 3.1. The Circulating SARS-CoV-2 Lineages in Cyprus from November 2022 to February 2024

In this study, 1627 SARS-CoV-2 near-whole-genome sequences obtained in Cyprus were analyzed for the November 2022 to February 2024 period. This analysis showed an extensive variety of 251 different lineages/sublineages (Table 1).

**Figure 1:**
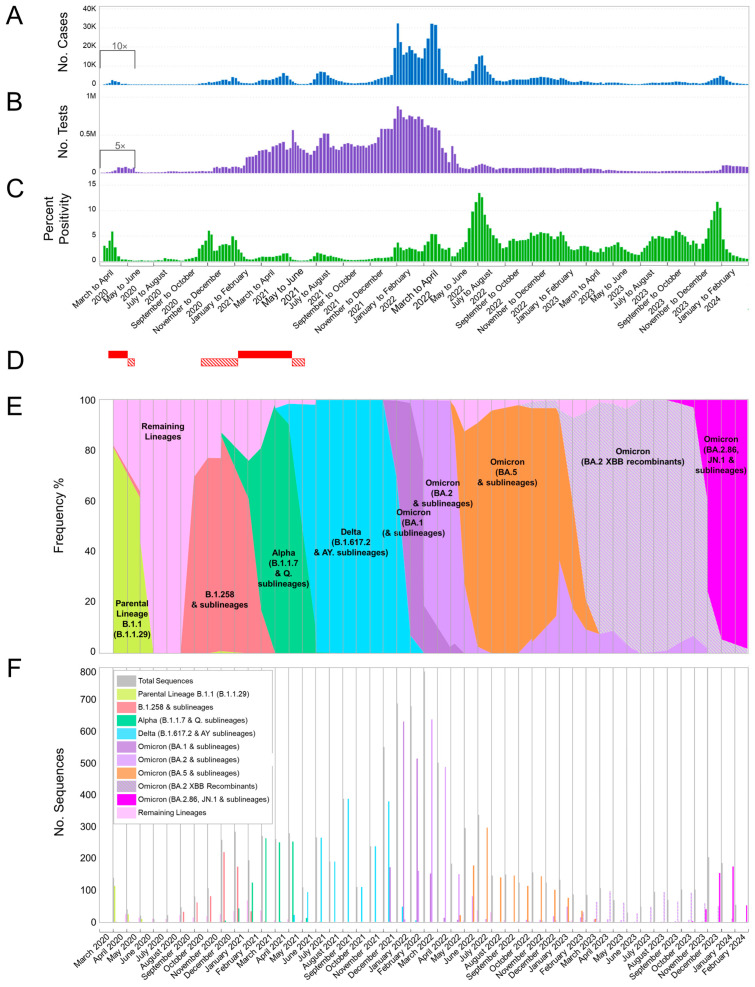
This study comprises a thorough analysis of SARS-CoV-2 cases, testing, percent positivity, and prevalent lineages in Cyprus, covering the period from March 2020 to February 2024. Specifically, we delve into the timeframe from November 2022 to February 2024, building upon our earlier research conducted between April 2020 and October 2021 [25,26,27]. (**A**) Illustrates the weekly tally of confirmed SARS-CoV-2 infections (dark blue columns). (**B**) Displays the overall count of SARS-CoV-2 tests, including both PCR and rapid tests, conducted on a weekly basis in Cyprus (purple columns). To improve clarity, the (**A**,**B**) data from 1 March to 17 May 2020 were multiplied by 10 and 5, respectively. (**C**) Depicts the calculated weekly percent positivity of SARS-CoV-2 testing (green columns). (**D**) Lockdowns (red rectangles) and partial lockdowns (diagonal red pattern rectangles) in Cyprus. The first lockdown was from 24 March to 3 May 2020, and the second lockdown was from 10 January to 9 May 2021. The first partial lockdown was from 4 May to 20 May 2020, the second from 23 October 2020 to 9 January 2021, and the third from 10 May 2021 to 10 June 2021. Brackets underneath (**A**–**C**) group weeks into approximately 2-month periods. (**E**,**F**) Depicts the frequency (proportion) and the number of sequences for the most prevalent lineages in Cyprus per month, respectively. Specifically, the sequences for lineages B.1.1.29 (parental lineage B.1.1), B.1.258 and sublineages, Alpha (B.1.1.7 and Q. sublineages), Delta (B.1.617.2 and AY. sublineages), Omicron 1 (BA.1 and sublineages), Omicron 2 (BA.2 and sublineages), Omicron 5 (BA.5 and sublineages), Omicron (BA.2) XBB recombinants, and Omicron (BA.2.86, JN.1 and sublineages) (including recombinants containing JN.1) are represented in bright green, red, green, light blue, purple, lilac, orange, lilac-gray stripes, and magenta, respectively. The “Remaining Lineages” shown in pink were determined by subtracting the monthly sequences of the lineages mentioned above from the total number of sequences indicated in gray. Sequencing results were provided from April 2020 and onwards.

**Figure 2:**
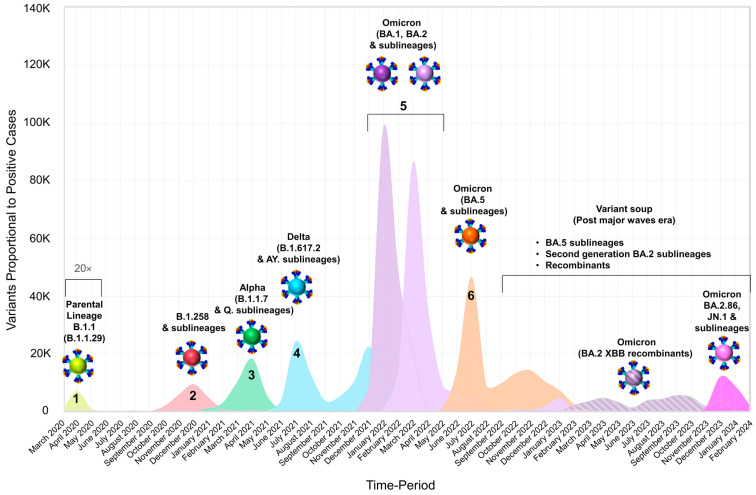
The chronological development and waves of SARS-CoV-2 in Cyprus from March 2020 to February 2024. The figure illustrates the progression of the SARS-CoV-2 epidemic in Cyprus and is based on our previous publications with sequencing data from April 2020 to October 2021 [25,26,27]. The illustration employs a proportional scaling of the number of SARS-CoV-2 infections (March 2020 to February 2024) to the prevalence of SARS-CoV-2 variants, as shown in Figure 1A,E,F. The SARS-CoV-2 infections proportional to sequences beneath the black bracket (until May 2020) have been multiplied by 20 to improve their visibility. The smooth line chart includes B.1.1.29 (parental lineage B.1.1), B.1.258 and sublineages, Alpha (B.1.1.7 and Q. sublineages), Delta (B.1.617.2 and AY. sublineages), Omicron 1 (BA.1 and sublineages), Omicron 2 (BA.2 and sublineages), and Omicron 5 (BA.5 and sublineages), which were all part of epidemiological waves indicated by the numbers 1–6. Following the sixth wave was a period with a plethora of different Omicron lineages (“variant soup”: BA.5 sublineages, BA.2 sublineages, and recombinants), none of which had reached the dominance of previous Omicron waves. The lineages of the six waves are shown in bright green, red, green, light blue, purple, lilac, and orange, respectively. The BA.2 XBB recombinants, highly prevalent during the “variant soup”, are depicted with lilac-gray stripes, while Omicron BA.2.86 and JN.1 and sublineages are shown in magenta, and the remaining lineages are shown in pink.

The first four-month period of this study (November 2022–February 2023) (Table 1) was dominated by Omicron 5 lineages (Figure 1E,F and Figure 2), with 351 out of 492 sequences (71.34%). Specifically, there was a high variability of Omicron 5 (*n* = 57 lineages), with BQ.1.1 being the most common Omicron 5 sublineage during this period, comprising 94 out of 351 (26.78%) sequences. The next most common sublineages of Omicron 5 were BA.5.2.1 and BF.11, and they were significantly lower, with 20 out of 351 sequences (5.7%) and 17 out of 351 sequences (4.84%), respectively. The remaining non-Omicron 5 sequences (28.46%, 140/492) identified during this period were Omicron 2 sublineages and recombinants (Table 1 and Figure 1E,F). Additionally, one Omicron 4 lineage was identified. This pattern of lineage prevalence, predominantly Omicron 5 with a smaller proportion of Omicron 2 and Omicron 4, resembled that observed during the final period of our previous study (July 2022–October 2022) [27]. However, during that period, Omicron 2 had a very low prevalence (2.77%, 19/686), in contrast to the relatively higher prevalence observed in the first period of the current study. Importantly, this four-month period (November 2022–February 2023) marked the first increase of Omicron 2 XBB* recombinant cases, which first appeared during the previous period (Figure 1E,F and Figure 2). Despite the rise of XBB* sequences during this period, which were approximately equal to Omicron 5 for the month of February 2023 (Figure 1E,F), the levels of new cases remained similar to those at the end of the previous four-month period and even started dropping in February (Figure 1A–C). Furthermore, the percent positivity dropped after January 2023 (Figure 1A–C).

The pattern of lineage representation shifted towards Omicron 2 XBB* recombinants over the next four-month period, March–June 2023 (Table 1 and Figure 1E,F and Figure 2). During this second period, Omicron 2 XBB* recombinants dominated with 245 out of 286 total sequences (85.66%) (Table 1). Among the highly variable XBB* recombinants of this period (*n* = 55 lineages), the most common was XBB.1.9.1, accounting for 22.86% of XBB* sequences (56/245), followed by XBB.1.5 (15.92%, 39/245) and EG.1 (10.61%, 26/245) (Table 1). In contrast, the previously dominant Omicron 5 group of lineages from the November 2022–February 2023 period only comprised 11 out of 286 sequences (3.85%) during this period (March–June 2023). It was also noteworthy that Omicron 5 sequences were only identified during the initial month of this period, in March 2023, with no other occurrences during this period. Other Omicron 2 lineages (BA.2.75*) and non-XBB recombinants (XAY.1.1.2, XBF.7*, and XBU) only comprised 8.04% (23/286) and 2.45% (7/286) of the sequences, respectively (Table 1). Despite this shift in lineage dominance, the overall number of cases continued to drop, while percent positivity fluctuated until it ultimately declined by the end of this period (Figure 1A–C and Figure 2).

The following four-month period, July to October 2023, marked the first phase after the sixth wave without any Omicron 5 sequences (Table 1 and Figure 1E,F and Figure 2). During this third period, only Omicron 2 sequences were circulating, with the diverse group of Omicron 2 XBB* recombinants continuing to dominate, comprising 297 out of 312 sequences (95.19%), with 72 different XBB* lineages (Table 1 and Figure 1E,F and Figure 2). Specifically, the most prevalent XBB* lineage during this period was GE.1, accounting for 19.19% (57/297) of the sequences, followed by EG.6.1 with 6.4% (19/297) and HK.15 with 5.05% (15/297). Additionally, XBB.1.9.1, which was dominant in the previous period, only accounted for 2.02% (6/297) of the XBB* sequences (Table 1). Despite no longer being the most prevalent in this new period, XBB.1.9.1 remained the most represented XBB* lineage over the course of the entire study (Table 1). Throughout the dominance of the diverse XBB* lineages, there were no significant changes in the number of new cases; however, the percent positivity was higher than the previous period (Figure 1A–C). Additionally, by the end of this third study period, the first identification of BA.2.86 (parental) and its JN* sublineages was observed, comprising only 1.28% (4/312) of the sequences (Table 1).

The final four-month period, November 2023 to February 2024, witnessed the fall of the XBB* group and the rise of BA.2.86 and its sublineages to dominance (77.47%, 416/537 total sequences) (Table 1 and Figure 1E,F and Figure 2). Even though the latter group of lineages was identified during September and October 2023 of the previous period (July–October 2023), it quickly overcame the XBB* group by December 2023 (Figure 1E,F). In January of 2024, the BA.2.86* group and, specifically, JN.1 and their sublineages peaked, before eventually dropping by the last month of the sample collecting period during February 2024 (Figure 1E,F and Figure 2). During this four-month period, the most prevalent BA.2.86* lineage was JN.1, with 188 out 416 sequences (45.19%), followed by JN.1.1 with 66 sequences (15.87%) and, with 60 sequences, JN.1.4 (14.42%) (Table 1). Additionally, there was also a significant rise in new cases that resulted in a wavelet, characterized by BA.2.86*/JN* sequences, with a percent positivity that rivaled the levels of the sixth wave with Omicron 5 (Figure 1A–C). However, despite the introductions of new lineages throughout this whole study, such as XBB* and BA.2.86*/JN*, only the latter resulted in a rise of cases high enough to form a minor wave, and none rivaled the increase in cases of the previous major Omicron waves in Cyprus (Figure 1A–C and Figure 2).

### 3.2. Spike Protein Mutations of Lineages/Variants in Cyprus from November 2022 to February 2024

**Figure 3:**
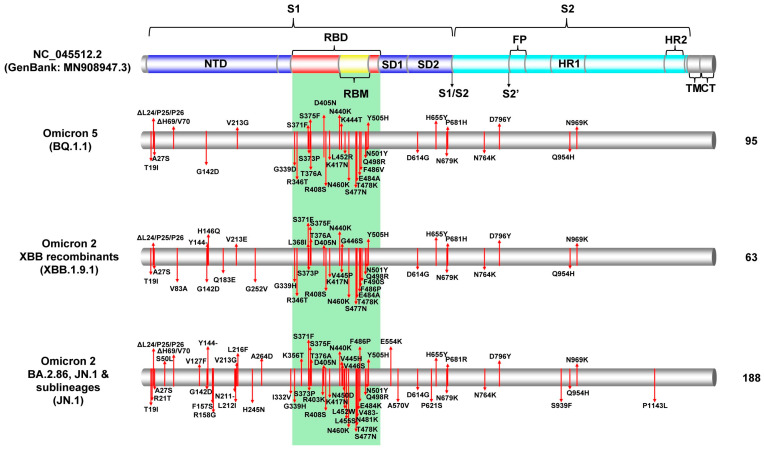
This diagram highlights the key S protein mutations in major SARS-CoV-2 lineages identified in Cyprus, based on the analysis of sequences collected from November 2022 to February 2024. It focuses on the predominant S protein mutations in the prevalent lineages shown in brackets: Omicron 5 (BQ.1.1), Omicron 2 XBB recombinants (XBB.1.9.1), and Omicron 2 BA.2.86 and JN.1 and sublineages (JN.1). The illustration includes a colored representation of the SARS-CoV-2 S protein’s crucial domains (GenBank: MN908947.3), such as the N-terminal domain (NTD), the receptor-binding domain (RBD) marked in red, the receptor-binding motif (RBM) shown in yellow, the subdomains 1 and 2 (SD1 and SD2), the fusion peptide (FP), subunit 1 (S1) colored blue, subunit 2 (S2) in cyan, the heptad repeats (HR), and the transmembrane domain (TM) and the cytoplasmic tail (CT), both in gray. Black arrows highlight the cleavage sites (S1/S2 and S2′). The area underlined in green denotes the receptor-binding domain (RBD) [41,42,43,44,45,46,47,48,49,50]. Red lines signal the sites of the most frequently identified mutations across the sequences within each lineage. On the figure’s right side, the total count of sequences evaluated per lineage is detailed.

The era following the sixth major epidemiological wave, represented by Omicron 5, was characterized by a high variability of variants, essentially all categorized as progeny of Omicron (Table 1). Indeed, all lineages that were identified in this study boasted the signature high number of S protein mutations (30 or more), depending on subvariant (Figure 3 and Tables S1 and S2). As shown above, for the first four months of this study, Omicron 5 sublineages were the most prevalent, with BQ.1.1 being the most commonly identified lineage (26.78%, 94/351 Omicron 5 sequences) encompassing 37 S protein mutations. This lineage retained the highest prevalence among all Omicron 5 sequences until the end of the sampling period, with 95 out of 362 (26.24%) Omicron 5 sequences (Table 1, Figure 3). The most commonly identified mutations for the BQ.1.1 lineage were T19I, ΔL24/P25/P26, A27S, ΔH69/V70, G142D, V213G, G339D, R346T, S371F, S373P, S375F, T376A, D405N R408S, K417N, N440K, K444T, L452R, N460K, S477N, T478K, E484A, F486V, Q498R, N501Y, Y505H, D614G, H655Y, N679K, P681H, N764K, D796Y, Q954H, and N969K (Figure 3 and Table S1). The next most common Omicron 5 lineages of the entire sampling period were BA.5.2.1 and BF.11. BA.5.2.1 (5.52%, 20/362), and they lacked the R346T, K444T, and N460K mutations, in contrast to the BQ.1.1 lineage, while BF.11 (4.7%, 17/362) lacked K444T and N460K. However, such mutations were found within different lineages among the Omicron 5 group. For example, within the BQ.1 parental lineage of BQ.1.1, the R346T mutation was typically not found, while, in other, less related Omicron 5 lineages, such as BA.5.2.34, this mutation was characteristic (Figure 3 and Table S1).

The XBB* Omicron 2 recombinant group of lineages was next to ascend to dominance and retained this position for the next nine months. During these nine months, XBB.1.9.1 was identified as the most common XBB* lineage (10.32%, 62/601), followed by GE.1 (9.65%, 58/601) and XBB.1.5 (6.66%, 40/601). The prevalence of these lineages remained the same for the entirety of the sampling period, with XBB.1.9.1 comprising 8.97% (63/702) of the XBB* sequences, GE.1 with 8.26% (58/702), and XBB.1.5 with 7.41% (52/702) (Figure 1 and Table 1). The 42 S protein mutations identified for the most common XBB* lineage, XBB.1.9.1, were T19I, ΔL24/P25/P26, A27S, V83A, G142D, ΔY144, H146Q, Q183E, V213E, G252V, G339H, R346T, L368I, S371F, S373P, S375F, T376A, D405N, R408S, K417N, N440K, V445P, G446S, N460K, S477N, T478K, E484A, F486P, F490S, Q498R, N501Y, Y505H, D614G, H655Y, N679K, P681H, N764K, D796Y, Q954H, and N969K (Figure 3). The differences between the S protein of the second-most prevalent lineage, GE.1 (45 mutations), and XBB.1.9.1 were as follows: GE.1 encompassed ΔN185, F186I, D253G, T478R, and P521S, which were not present in XBB.1.9.1. Conversely, XBB.1.9.1 had the G252V and T478K mutations, which were not present in GE.1. The S protein mutations were the same between XBB.1.5, the third-most prevalent XBB* lineage, and XBB.1.9.1 (Table S1). Nonetheless, similar to Omicron 5, different and recurring mutations can be observed across variants of the XBB* group. For example, the G252V mutation is commonly found in XBB.1* but not in XBB.2* (Figure 1 and Table S1). Additionally, the L452R mutation, which recurs in Omicron 5, Delta, and other variants, was not observed in the XBB* group [51] (Figure 3 and Table S1).

The last 3 months of the study were dominated by BA.2.86* and its sublineages (JN*, which succeeded the XBB* group, even leading to a wavelet) (Figure 1 and Figure 2). The most prevalent BA.2.86* lineage during this period was JN.1 (47.61%, 176/376), with the second spot shared by JN.1.1 and JN.1.4 (15.69%, 59/376). Similarly, these three lineages had the highest prevalence amongst the entirety of the BA.2.86* during the whole study period, with JN.1 again dominating (44.76%, 188/420), followed by JN.1.1 (15.71%, 66/420) and then JN.1.4 (14.29%, 60/420) (Table 1 and Figure 1). This group of lineages had significantly more mutations within the S protein compared to other Omicron variants, both within Omicron 2 and XBB recombinants, as well as Omicron 5 found within this study (Figure 3 and Table S1). The 60 S protein mutations identified in JN.1 were T19I, R21T, ΔL24/P25/P26, A27S, S50L, ΔH69/V70, V127F, G142D, ΔY144, F157S, R158G, ΔN211, L212I, V213G, L216F, H245N, A264D, I332V, G339H, K356T, S371F, S373P, S375F, T376A, R403K, D405N, R408S, K417N, N440K, V445H, G446S, N450D, L452W, L455S, N460K, S477N, T478K, N481K, ΔV483, E484K, F486P, Q498R, N501Y, Y505H, E554K, A570V, D614G, P621S, H655Y, N679K, P681R, N764K, D796Y, S939F, Q954H, N969K, and P1143L. All three of these lineages had the same S protein mutations; however, the insertion ins16MPLF could also be identified (Tables S1 and S2) [52,53]. This group of lineages also exhibited recurring mutations, including but not limited to, ΔH69/V70, G142D, and ΔY144, which have been previously identified in some variants such as Alpha, Delta, and Omicron 5 [51,54,55] (Figure 3 and Table S1).

**Figure 4:**
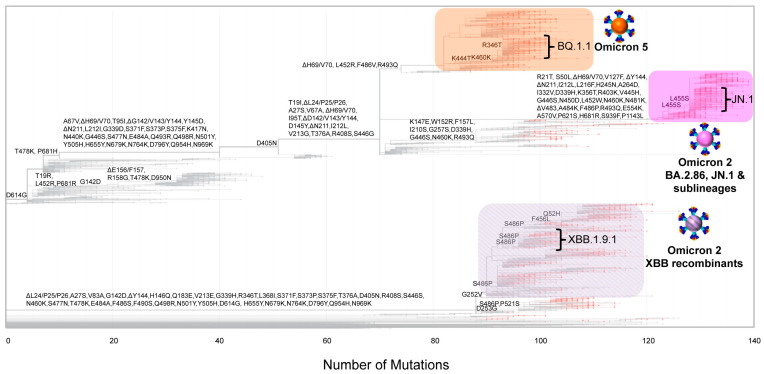
Illustration of the evolutionary relationships and mutation patterns within key SARS-CoV-2 lineages, offering valuable insights into the viral dynamics in Cyprus from November 2022 to February 2024. The maximum likelihood tree was created using Nextclade (https://clades.nextstrain.org, accessed on 14 July 2024 [30]) to depict the evolution of mutations. The figure covers a wide array of SARS-CoV-2 lineages (see Table 1) and focuses on the most prevalent lineages of Omicron 5 (BQ.1.1), Omicron 2 XBB recombinants (XBB.1.9.1), and Omicron 2 BA.2.86, JN.1 and sublineages (JN.1), indicated within the brackets. These sequences are highlighted in orange, lilac-gray stripes, and magenta rectangles, respectively. Red dots on the tree indicate the sequences used in this study.

**Figure 5:**
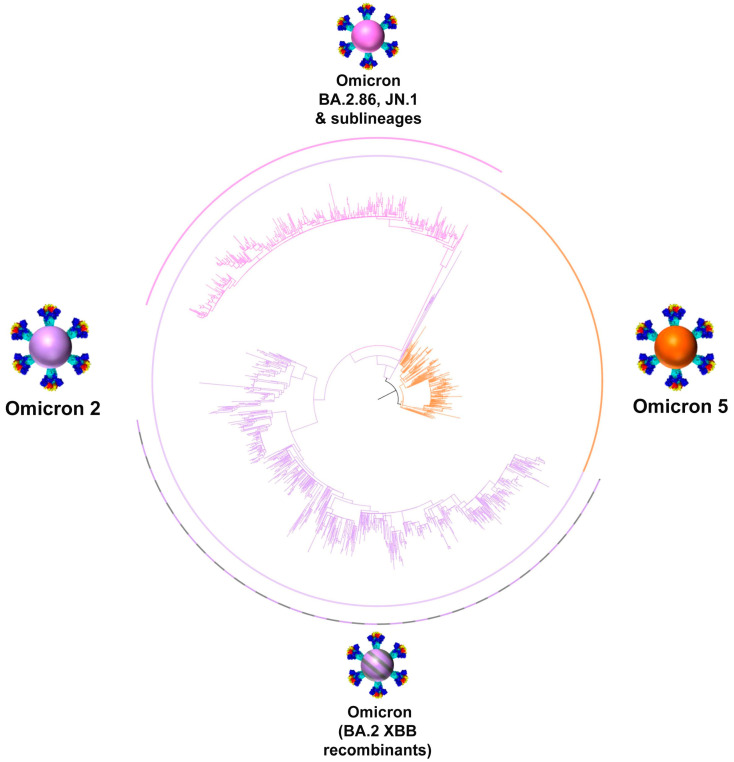
Maximum likelihood phylogenetic tree of Cypriot SARS-CoV-2 genomes from November 2022 to February 2024. The sequences classified as Omicron 5 are highlighted with an orange arc, corresponding to the relevant branches. Sequences with Omicron 2 are shown with a lilac arc. Within Omicron 2, the BA.2.86 and JN.1 group of sequences are marked in magenta, and BA.2 XBB recombinants are shown with lilac-gray.

The SARS-CoV-2 infection has entered an endemic phase, characterized by a diverse array of SARS-CoV-2 lineages, a variant soup, with varying and recurring mutations [13]. Figure 4 and Figure 5 illustrate the intra- and inter-genetic variability among the different groups in this study. Specifically, they show how variants cluster together and are delineated into distinct clades, characterized by mutations that commonly appear across different groups. The most variable group was XBB*, with 136 different lineages, followed by Omicron 5 with 61 lineages and BA.2.86* (including JN.1 and sublineages) with 23 lineages (Table 1 and Figure 4 and Figure 5). The last group to dominate in this study, BA.2.86*, not only encompassed close to twice the number of mutations but it also contained mutations from both other clades, including ΔH69/V70, which was not commonly found in Omicron 2 sublineages (Table S1 and Figure 3 and Figure 4) [51,54,55]. Additionally, Figure 3 and Figure 4 demonstrate that, in general, the S protein accumulated more mutations as the pandemic progressed, and this phenomenon can be observed between the variants found in this study and in comparison to previous variants such as Alpha and Delta [8]. The majority of these mutations has been located within the S1 subunit, particularly in the receptor-binding domain (RBD) and the N-terminal domain (NTD), which is in agreement with previous findings [8,27,56]. Thus, throughout this study, Cyprus has experienced the dying embers of Omicron 5, followed by the long reign of XBB*s and, ultimately, the usurping of BA.2.86 and JN.1 sublineages that caused a small surge of new infections (Figure 2). As such, during this timeframe, the genetic/phylogenetic makeup of the majority of the lineages found in Cyprus fall within Omicron 2, which includes both Omicron 2 sublineages and recombinants, encompassing high-risk mutations that have also appeared in previously prevalent variants (Table S1 and Figure 3, Figure 4 and Figure 5) [55,57].

## 4. Discussion

In this investigation, we analyzed 1627 SARS-CoV-2 whole-genome sequences obtained in Cyprus from November 2022 to February 2024. Our analysis revealed significant diversity, identifying 251 different lineages/sublineages. This indicates a highly variable period compared to previous study periods: from October 2021 to October 2022, we identified 167 different lineages from 4700 sequences; from November 2020 to October 2021, 61 distinct lineages from 2352 sequences; and from April 2020 to January 2021, 34 different lineages from 596 sequences [25,26,27]. This emphasizes the ongoing evolution of SARS-CoV-2 and the emergence of diverse lineages, showing the virus’s dynamic nature and the persistence of certain variants.

These lineages, which were classified into different groups, have alternated in dominance since the beginning of this study period. The first group that was the most prevalent in this study was Omicron 5, which continued to dominate even after the sixth wave and included various sublineages, with BQ.1.1 being the most prevalent. Moreover, during the period of Omicron 5’s dominance from November 2022 to February 2023, SARS-CoV-2 cases were significantly lower compared to the peak of the sixth wave (Figure 1 and Figure 2). This decline in SARS-CoV-2 cases occurred despite Omicron 5 progeny, such as BQ.*, displacing the dominant lineages BA.5.1 and BA.5.2, which had driven the sixth wave (Figure 1 and Figure 2). Similar trends were observed in Europe and around the world, where these subvariants were reported to be more infectious and had higher immune evasion capacities due to mutations like K444T, N460K, and R346T [19,58,59,60]. However, these subvariants were still not sufficient to drive a major epidemiological wave, as with the initial Omicron variants, likely due to hybrid immunity resulting from both natural infections and vaccinations [58,61,62].

Thus, the measures taken by governmental entities, influenced by social, medical, and epidemiological studies, have allowed for the relaxation of measures and travel restrictions in an effort to return to a life of relative normalcy. The progression of the pandemic and ensuing waves of infection have underscored the importance of focusing on community well-being and reducing the fatigue caused by the constant state of alert. In return, these actions rely on empowering individuals with knowledge and strategies to protect themselves against the virus. Consequently, these steps were crucial for returning and maintaining normalcy and were continued at the start of this study period when Omicron 5 subvariants were prominent and as the next variants, XBB*, were emerging (Ministry announcements 31 August and 2 November 2022) [63,64,65].

Indeed, the scale of the infections did not rise to the level of previous Omicron waves following the change of dominance from Omicron 5 to the XBB* recombinants, a group of variants distinguished as an amalgamation of two Omicron 2 variants, BJ.1 and BM.1.1.1 (Figure 1 and Figure 2) [29]. Despite BQ’s high transmissibility, it was reported to be globally outcompeted by the even more infectious XBBs [66,67,68]. However, both of these Omicron 5 and Omicron 2 derivatives exhibit a vastly different antigenic profile and infectivity when compared to earlier isolates, such as Alpha and Delta [69]. XBB variants dominated the majority of the study period (March to November 2023) in Cyprus. However, during this time, the number of infections remained consistently low (Figure 1 and Figure 2). This outcome was likely attributed to various factors, including the coordinated efforts of policymakers and researchers who closely monitored both local and global developments. In Cyprus, as early as January 2023 and continuing through May 2023, preventive measures were slightly increased in anticipation of the XBB variant’s global migrations. These included requiring negative PCR tests from regions heavily affected by the variant. Additionally, mask mandates were reinforced in crowded areas such as airports and were obligatory for workers and visitors in settings with vulnerable groups of individuals. Although no longer mandatory, recommendations remain for individuals testing positive to isolate for at least five days and to wear a mask for up to 10 days when around others (11 11 January 2023 and 9 May 2023) [65]. These guidelines, along with the established precedents throughout the pandemic and hybrid immunity, helped keep the highly variable and diverse XBB variants (XBB.1.9.1, GE.1, and XBB.1.5) at relatively low levels for nearly a year [58,61,62,65,70,71]. This period of dominance finally gave way to the rise of the BA.2.86 and JN.1 variants.

This group of variants and, specifically, JN.1, characterized by a high number of S protein mutations (60 mutations), rapidly displaced XBB, leading to the first notable increase in infections since the Omicron BA.5 wave (Table 1 and Table S1 and Figure 1, Figure 2 and Figure 3). However, this surge resulted in a smaller wavelet, with cases rising in December 2023, peaking in January 2024, and declining by February 2024 (Figure 1 and Figure 2). Even in the global scene, this variant became the dominant strain by the end of 2023 and early 2024, driving localized surges of infection [72]. JN.1 not only contains nearly twice as many mutations as the previous XBB and Omicron BA.5 variants but also includes critical ones such as the L455S mutation in the receptor-binding motif, which has been associated with increased transmissibility and enhanced immune evasion [72,73,74]. This enriched antigenic profile was the driving force behind the replacement of previous Omicron variants and the continued worldwide spread [72,73,74].

However, despite this difference of JN.1 in antigenic profiles compared to earlier Omicron variants, the impact was not as significant as the Alpha, Beta, Gamma, and Delta VOCs when compared to Omicron. JN.1 was even considered as low risk, with predictions that it would be quickly outcompeted by its progeny [75,76]. Nonetheless, caution must always be exercised, as hybrid immunity from vaccines and natural infections may not always keep up with the emergence of resistant variants, necessitating a constant monitoring and updating of policies and technologies [72,77,78,79]. In Cyprus, this sudden rise in cases was swiftly managed by applying lessons from the past. This included the requirement of negative PCR tests and masks for visitation and work in crowded and vulnerable spaces, such as nursing homes and hospitals. Furthermore, vaccinations for employees and residents of nursing homes and other care facilities for the elderly and vulnerable groups were provided through mobile vaccination units of the Ministry of Health (3 January 2024 announcement) [80].

In conclusion, the ongoing evolution of SARS-CoV-2 continues to follow a dynamic pattern, with new variants periodically driving surges of new infections, regardless of seasonality. While Omicron 5 sublineages and XBB variants did not result in major waves, the virus’s antigenic diversity has remained a key factor in its ability to evade immunity and spread globally. In contrast, the JN.1 variant did trigger a small wave of infections, though it was swiftly and effectively managed through timely interventions. Lessons learned from earlier waves have equipped governments, researchers, and public health officials to better monitor and respond to these developments. Through vigilance, the adaptation of preventive measures, testing, and vaccinations, societies can continue to manage the emergence of new variants. Going forward, such strategies and mindsets will be necessary to address new variants as they emerge, ensuring that societies can adapt while maintaining a balance between public health and normalcy.

## Figures and Tables

**Table 1:** SARS-CoV-2 lineages identified from 1627 sequences in Cyprus from November 2022 to February 2024.

Time Period	Nov 2022–Feb 2023	Mar–Jun 2023	Jul–Oct 2023	Nov 2023–Feb 2024	Total
Lineage	Νumber of Sequences per Lineage (%)	Νumber of Sequences per Lineage (%)	Νumber of Sequences per Lineage (%)	Νumber of Sequences per Lineage (%)	Νumber of Sequences per Lineage (%)
BS.1.1	3 (0.61)	-	-	-	3 (0.18)
BA.2.3.20	3 (0.61)	-	-	-	3 (0.18)
CM.10	1 (0.2)	-	-	-	1 (0.06)
BA.2.75.2	1 (0.2)	-	-	-	1 (0.06)
CV.1	1 (0.2)	-	-	-	1 (0.06)
CH.1.1	15 (3.05)	1 (0.35)	-	-	16 (0.98)
CH.1.1.1	2 (0.41)	-	1 (0.32)	-	3 (0.18)
DV.6	2 (0.41)	3 (1.05)	-	-	5 (0.31)
DV.7.1.1	-	-	1 (0.32)	-	1 (0.06)
DV.7.1.3	-	-	2 (0.64)	-	2 (0.12)
DV.7.1.4	-	-	6 (1.92)	2 (0.37)	8 (0.49)
DV.7.1.5	-	-	1 (0.32)	-	1 (0.06)
CH.1.1.2	2 (0.41)	-	-	-	2 (0.12)
GP.1	-	1 (0.35)	-	-	1 (0.06)
BN.1	4 (0.81)	-	-	-	4 (0.25)
BN.1.1.1	3 (0.61)	-	-	-	3 (0.18)
FR.1	-	2 (0.7)	-	-	2 (0.12)
BN.1.3	19 (3.86)	10 (3.5)	-	-	29 (1.78)
BN.1.3.1	5 (1.02)	4 (1.4)	-	-	9 (0.55)
BN.1.5	5 (1.02)	-	-	-	5 (0.31)
BN.3.1	4 (0.81)	-	-	-	4 (0.25)
BN.1.10	1 (0.2)	-	-	-	1 (0.06)
BR.2.1	-	2 (0.7)	-	-	2 (0.12)
BR.3	17 (3.46)	-	-	-	17 (1.04)
BA.2.86	-	-	1 (0.32)		1 (0.06)
BA.2.86.1	-	-	-	26 (4.84)	26 (1.6)
JN.1	-	-	-	188 (35.01)	188 (11.56)
JN.1.1	-	-	-	66 (12.29)	66 (4.06)
JN.1.1.1	-	-	-	2 (0.37)	2 (0.12)
JN.1.2	-	-	1 (0.32)	5 (0.93)	6 (0.37)
JN.1.4	-	-	-	60 (11.17)	60 (3.69)
JN.1.5	-	-	-	1 (0.19)	1 (0.06)
JN.1.7	-	-	-	1 (0.19)	1 (0.06)
JN.1.8	-	-	-	1 (0.19)	1 (0.06)
JN.1.9	-	-	-	1 (0.19)	1 (0.06)
JN.1.16	-	-	-	3 (0.56)	3 (0.18)
JN.1.18	-	-	-	1 (0.19)	1 (0.06)
JN.1.22	-	-	-	1 (0.19)	1 (0.06)
JN.2	-	-	2 (0.64)	8 (1.49)	10 (0.61)
JN.3	-	-	-	6 (1.12)	6 (0.37)
JN.5	-	-	-	2 (0.37)	2 (0.12)
JN.6	-	-	-	4 (0.74)	4 (0.25)
JN.9	-	-	-	8 (1.49)	8 (0.49)
JN.10	-	-	-	28 (5.21)	28 (1.72)
BA.4.6	1 (0.2)	-	-	-	1 (0.06)
BA.5.1	4 (0.81)	-	-	-	4 (0.25)
BA.5.1.5	2 (0.41)	-	-	-	2 (0.12)
BA.5.1.10	4 (0.81)	-	-	-	4 (0.25)
BT.2	16 (3.25)	-	-	-	16 (0.98)
BA.5.2	13 (2.64)	-	-	-	13 (0.8)
BA.5.2.1	20 (4.07)	-	-	-	20 (1.23)
BF.1	1 (0.2)	-	-	-	1 (0.06)
BF.5	13 (2.64)	-	-	-	13 (0.8)
BF.7	16 (3.25)	-	-	-	16 (0.98)
BF.7.1	9 (1.83)	-	-	-	9 (0.55)
BF.7.4.1	6 (1.22)	-	-	-	6 (0.37)
BF.7.6	1 (0.2)	-	-	-	1 (0.06)
BF.7.7	2 (0.41)	-	-	-	2 (0.12)
BF.7.8	6 (1.22)	-	-	-	6 (0.37)
BF.7.13.2	1 (0.2)	-	-	-	1 (0.06)
BF.7.20	-	1 (0.35)	-	-	1 (0.06)
BF.7.22	1 (0.2)	-	-	-	1 (0.06)
BF.7.23	2 (0.41)	-	-	-	2 (0.12)
BF.7.24	1 (0.2)	-	-	-	1 (0.06)
BF.10	1 (0.2)	-	-	-	1 (0.06)
BF.11	17 (3.46)	-	-	-	17 (1.04)
BF.11.2	14 (2.85)	-	-	-	14 (0.86)
BF.14	5 (1.02)	-	-	-	5 (0.31)
BF.40	11 (2.24)	-	-	-	11 (0.68)
BA.5.2.6	5 (1.02)	-	-	-	5 (0.31)
CR.1.3	1 (0.2)	-	-	-	1 (0.06)
BA.5.2.20	5 (1.02)	-	-	-	5 (0.31)
CK.2	1 (0.2)	-	-	-	1 (0.06)
BA.5.2.26	2 (0.41)	-	-	-	2 (0.12)
BA.5.2.34	15 (3.05)	-	-	-	15 (0.92)
BA.5.2.36	2 (0.41)	-	-	-	2 (0.12)
DQ.1	1 (0.2)	-	-	-	1 (0.06)
BA.5.2.60	1 (0.2)	-	-	-	1 (0.06)
BE.1.1	1 (0.2)	-	-	-	1 (0.06)
BQ.1	3 (0.61)	-	-	-	3 (0.18)
BQ.1.1	94 (19.11)	1 (0.35)	-	-	95 (5.84)
BQ.1.1.1	3 (0.61)	-	-	-	3 (0.18)
BQ.1.1.3	2 (0.41)	-	-	-	2 (0.12)
BQ.1.1.4	1 (0.2)	-	-	-	1 (0.06)
BQ.1.1.7	1 (0.2)	-	-	-	1 (0.06)
BQ.1.10	1 (0.2)	-	-	-	1 (0.06)
BQ.1.10.2	11 (2.24)	-	-	-	11 (0.68)
BQ.1.13.1	2 (0.41)	1 (0.35)	-	-	3 (0.18)
BQ.1.1.15	2 (0.41)	-	-	-	2 (0.12)
BQ.1.18	-	2 (0.7)	-	-	2 (0.12)
BQ.1.1.20	1 (0.2)	-	-	-	1 (0.06)
BQ.1.1.22	1 (0.2)	-	-	-	1 (0.06)
BQ.1.1.44	1 (0.2)	-	-	-	1 (0.06)
BQ.1.1.45	-	1 (0.35)	-	-	1 (0.06)
BQ.1.1.47	1 (0.2)	-	-	-	1 (0.06)
BQ.1.1.55	1 (0.2)	-	-	-	1 (0.06)
BQ.1.1.56	1 (0.2)	-	-	-	1 (0.06)
BQ.1.1.66	1 (0.2)	-	-	-	1 (0.06)
BQ.1.1.79	9 (1.83)	4 (1.4)	-	-	13 (0.8)
DN.1	1 (0.2)	-	-	-	1 (0.06)
EF.1.1	2 (0.41)	-	-	-	2 (0.12)
ET.1	-	1 (0.35)	-	-	1 (0.06)
BQ.1.2	5 (1.02)	-	-	-	5 (0.31)
BQ.1.5	5 (1.02)	-	-	-	5 (0.31)
BQ.1.8	1 (0.2)	-	-	-	1 (0.06)
BA.5.9	1 (0.2)	-	-	-	1 (0.06)
XBB.1	6 (1.22)	-	-	-	6 (0.37)
XBB.1.5	12 (2.44)	39 (13.64)	1 (0.32)	-	52 (3.2)
XBB.1.5.1	-	1 (0.35)	-	-	1 (0.06)
EM.1	-	1 (0.35)	-	-	1 (0.06)
XBB.1.5.11	-	1 (0.35)	-	-	1 (0.06)
XBB.1.5.12	1 (0.2)	3 (1.05)	-	-	4 (0.25)
XBB.1.5.13	-	1 (0.35)	-	-	1 (0.06)
XBB.1.5.14	-	1 (0.35)	-	-	1 (0.06)
XBB.1.5.15	-	1 (0.35)	-	-	1 (0.06)
FD.1	-	1 (0.35)	-	-	1 (0.06)
FD.3	-	1 (0.35)	-	-	1 (0.06)
XBB.1.5.18	1 (0.2)	3 (1.05)	-	-	4 (0.25)
XBB.1.5.24	1 (0.2)	1 (0.35)	-	-	2 (0.12)
EU.1.1	-	10 (3.5)	1 (0.32)	-	11 (0.68)
XBB.1.5.28	-	4 (1.4)	2 (0.64)	-	6 (0.37)
XBB.1.5.33	-	2 (0.7)	-	-	2 (0.12)
XBB.1.5.35	-	1 (0.35)	-	-	1 (0.06)
XBB.1.5.37	-	2 (0.7)	-	-	2 (0.12)
XBB.1.5.38	-	1 (0.35)	-	-	1 (0.06)
XBB.1.5.46	-	1 (0.35)	-	-	1 (0.06)
GV.1	3 (0.61)	1 (0.35)	-	-	4 (0.25)
XBB.1.5.49	-	2 (0.7)	1 (0.32)	-	3 (0.18)
XBB.1.5.52	6 (1.22)	6 (2.1)	-	-	12 (0.74)
XBB.1.5.55	-	5 (1.75)	-	-	5 (0.31)
XBB.1.5.62	1 (0.2)	-	-	-	1 (0.06)
XBB.1.5.63	2 (0.41)	-	-	-	2 (0.12)
XBB.1.5.69	-	1 (0.35)	-	-	1 (0.06)
GK.1.1	-	-	3 (0.96)	-	3 (0.18)
GK.1.1.1	-	-	1 (0.32)	-	1 (0.06)
GK.8.1	-	-	-	2 (0.37)	2 (0.12)
XBB.1.5.90	-	4 (1.4)	-	-	4 (0.25)
JD.1.1	-	-	2 (0.64)	4 (0.74)	6 (0.37)
JD.1.1.1	-	-	-	1 (0.19)	1 (0.06)
JD.1.1.5	-	-	-	1 (0.19)	1 (0.06)
JD.1.2	-	-	1 (0.32)	-	1 (0.06)
JD.2	-	1 (0.35)	-	-	1 (0.06)
XBB.1.9.1	1 (0.2)	56 (19.58)	6 (1.92)	-	63 (3.87)
FL.1.5.1	-	-	12 (3.85)	5 (0.93)	17 (1.04)
HN.4	-	-	1 (0.32)	-	1 (0.06)
HN.4.2	-	-	1 (0.32)	-	1 (0.06)
HN.5	-	-	2 (0.64)	4 (0.74)	6 (0.37)
KC.1	-	-	-	4 (0.74)	4 (0.25)
FL.2	-	1 (0.35)	-	-	1 (0.06)
FL.3	-	2 (0.7)	-	-	2 (0.12)
FL.3.1	2 (0.41)	17 (5.94)	-	-	19 (1.17)
FL.5	-	2 (0.7)	-	-	2 (0.12)
FL.10	-	3 (1.05)	-	-	3 (0.18)
FL.13	1 (0.2)	-	1 (0.32)	-	2 (0.12)
FL.15	-	-	6 (1.92)	-	6 (0.37)
FL.15.1.1	-	-	1 (0.32)	3 (0.56)	4 (0.25)
FL.17	1 (0.2)	-	-	-	1 (0.06)
FL.18	-	2 (0.7)	1 (0.32)	-	3 (0.18)
FL.20	-	-	1 (0.32)	-	1 (0.06)
FL.25	-	-	1 (0.32)	-	1 (0.06)
FL.26	-	1 (0.35)	-	-	1 (0.06)
FL.40	-	1 (0.35)	-	-	1 (0.06)
XBB.1.9.2	-	1 (0.35)	-	-	1 (0.06)
EG.1	-	26 (9.09)	-	-	26 (1.6)
EG.1.2	-	2 (0.7)	-	-	2 (0.12)
EG.1.3	-	1 (0.35)	-	-	1 (0.06)
EG.1.6	-	3 (1.05)	-	-	3 (0.18)
EG.5	-	-	1 (0.32)	-	1 (0.06)
EG.5.1	-	-	3 (0.96)	-	3 (0.18)
EG.5.1.1	-	-	10 (3.21)	7 (1.3)	17 (1.04)
HK.1.2	-	-	-	2 (0.37)	2 (0.12)
HK.2	-	-	1 (0.32)	-	1 (0.06)
HK.3	-	-	6 (1.92)	18 (3.35)	24 (1.48)
HK.3.1	-	-	-	2 (0.37)	2 (0.12)
HK.3.2	-	-	-	2 (0.37)	2 (0.12)
HK.3.9	-	-	-	1 (0.19)	1 (0.06)
HK.6	-	-	13 (4.17)	1 (0.19)	14 (0.86)
HK.11	-	-	1 (0.32)	-	1 (0.06)
HK.15	-	-	15 (4.81)	3 (0.56)	18 (1.11)
HK.26	-	-	1 (0.32)	3 (0.56)	4 (0.25)
EG.5.1.3	-	-	9 (2.88)	2 (0.37)	11 (0.68)
JG.2	-	-	1 (0.32)	-	1 (0.06)
JG.3	-	-	3 (0.96)	11 (2.05)	14 (0.86)
EG.5.1.4	-	-	-	1 (0.19)	1 (0.06)
EG.5.1.6	-	-	11 (3.53)	1 (0.19)	12 (0.74)
HV.1	-	-	6 (1.92)	13 (2.42)	19 (1.17)
HV.1.1	-	-	-	2 (0.37)	2 (0.12)
EG.5.1.7	-	-	1 (0.32)	-	1 (0.06)
JR.1.1	-	-	1 (0.32)	-	1 (0.06)
EG.6.1	-	1 (0.35)	19 (6.09)	-	20 (1.23)
EG.10.1.1	-	-	2 (0.64)	-	2 (0.12)
EG.13	-	1 (0.35)	-	-	1 (0.06)
EG.14	-	1 (0.35)	-	-	1 (0.06)
XBB.1.16	-	5 (1.75)	2 (0.64)	-	7 (0.43)
XBB.1.16.1	-	1 (0.35)	10 (3.21)	-	11 (0.68)
FU.1	-	1 (0.35)	-	-	1 (0.06)
FU.2.1	-	-	3 (0.96)	-	3 (0.18)
FU.5	-	-	1 (0.32)	-	1 (0.06)
XBB.1.16.2	-	3 (1.05)	-	-	3 (0.18)
GY.1	-	-	3 (0.96)	-	3 (0.18)
XBB.1.16.6	-	-	2 (0.64)	1 (0.19)	3 (0.18)
JF.1.1	-	-	3 (0.96)	-	3 (0.18)
JF.4	-	-	1 (0.32)	-	1 (0.06)
XBB.1.16.10	-	1 (0.35)	-	-	1 (0.06)
XBB.1.16.11	-	-	10 (3.21)	4 (0.74)	14 (0.86)
XBB.1.16.13	-	-	9 (2.88)	-	9 (0.55)
HF.1	-	-	3 (0.96)	1 (0.19)	4 (0.25)
XBB.1.16.15	-	-	2 (0.64)	-	2 (0.12)
XBB.1.16.18	-	1 (0.35)	-	-	1 (0.06)
XBB.1.16.21	-	-	2 (0.64)	-	2 (0.12)
XBB.1.16.27	-	-	1 (0.32)	-	1 (0.06)
FE.1.1	-	2 (0.7)	1 (0.32)	-	3 (0.18)
FE.1.1.5	-	-	2 (0.64)	-	2 (0.12)
GW.4	-	1 (0.35)	-	-	1 (0.06)
XBB.1.22.1	-	1 (0.35)	-	-	1 (0.06)
FY.5.1.1	-	-	-	1 (0.19)	1 (0.06)
FY.5.4	-	-	1 (0.32)	-	1 (0.06)
XBB.1.24.1	-	1 (0.35)	-	-	1 (0.06)
XBB.1.16.28	-	-	-	1 (0.19)	1 (0.06)
FW.1.1	-	-	2 (0.64)	-	2 (0.12)
XBB.1.16.31	-	-	-	1 (0.19)	1 (0.06)
JC.2	-	-	2 (0.64)	-	2 (0.12)
XBB.1.41.2	-	-	1 (0.32)	-	1 (0.06)
XBB.1.42	-	9 (3.15)	2 (0.64)	-	11 (0.68)
XBB.2	1 (0.2)	-	-	-	1 (0.06)
XBB.2.3.2	-	-	1 (0.32)	-	1 (0.06)
HH.2.1	-	-	1 (0.32)	-	1 (0.06)
XBB.2.3.3	-	3 (1.05)	-	1 (0.19)	4 (0.25)
GJ.1.2	-	-	-	1 (0.19)	1 (0.06)
GJ.4	-	-	2 (0.64)	-	2 (0.12)
GJ.5.1	-	-	1 (0.32)	-	1 (0.06)
GZ.1	-	-	7 (2.24)	-	7 (0.43)
GE.1	-	-	57 (18.27)	1 (0.19)	58 (3.56)
GE.1.3	-	-	1 (0.32)	-	1 (0.06)
GE.1.5	-	-	3 (0.96)	3 (0.56)	6 (0.37)
GE.1.6	-	-	1 (0.32)	1 (0.19)	2 (0.12)
GS.1	-	-	1 (0.32)	-	1 (0.06)
GS.4.1	-	-	3 (0.96)	10 (1.86)	13 (0.8)
GS.4.1.1	-	-	2 (0.64)	-	2 (0.12)
XBB.3.2	2 (0.41)	-	-	-	2 (0.12)
XAY.1.1.2	-	1 (0.35)	-	-	1 (0.06)
XBF	1 (0.2)	-	-	-	1 (0.06)
XBF.7	-	4 (1.4)	-	-	4 (0.25)
XBF.7.1	2 (0.41)	1 (0.35)	-	-	3 (0.18)
XBU	7 (1.42)	1 (0.35)	-	-	8 (0.49)
XCA	1 (0.2)	-	-	-	1 (0.06)
XCH.1	-	-	1 (0.32)	-	1 (0.06)
XDA.1	-	-	4 (1.28)	-	4 (0.25)
XDD	-	-	-	2 (0.37)	2 (0.12)
XDD.1	-	-	-	1 (0.19)	1 (0.06)
XDK	-	-	-	1 (0.19)	1 (0.06)
Total	492	286	312	537	1627 (100)

## Data Availability

Following the publication of this paper, the analyzed SARS-CoV-2 sequences will be made available within the GISAID database [31]. Only the sequences deemed “good quality” under the “qc.overallStatus” parameter will be provided to GISAID. This opted to circumvent potential misinterpretations of mutations, which may emerge during sequencing and assembly [5,30]. The dissemination of high-quality sequences allows the utilization of dependable and accurate genetic data related to SARS-CoV-2, thereby enhancing further investigative endeavors and knowledge concerning the virus.

## Supplementary Materials

The following supporting information can be downloaded at https://www.mdpi.com/article/10.3390/v16111686/s1: Table S1: Common S-protein Mutations of Omicron Variants that were Identified in this Dataset; Table S2: Uncommon S-protein Mutations of Omicron Variants that were Identified in this Dataset. Reference [81] is cited in supplementary file.
