# Supplementary material for: Into the Cauldron of the Variant Soup: Insights into the Molecular Epidemiology and Transition to Endemicity of SARS-CoV-2 in Cyprus (November 2022–February 2024)
Source: Viruses. 2024 Oct 29;16(11):1686. doi: 10.3390/v16111686 (PMC11599100; doi:10.3390/v16111686)
Supplement: Supplementary file 1 [file viruses-16-01686-s001.zip › viruses-3250868-Supplementary.pdf]

Table S1. Common S-protein Mutations of Omicron Variants that were Identified in this Dataset[a].

Total Sequences[b]	Lineage[c]	Mutations and Number of Sequences Identified for Each Mutation[d]										
3	BS.1.1	T19I	L24-	P25-	P26-	A27S	G142D	Y144-	ins212:NGEc	V213G	G257V	G339D
		3	3	3	3	3	3	3	3	3	3	3
		R346T	K356T	S371F	S373P	S375F	T376A	D405N	R408S	K417N	N440K	L452R
		3	3	3	3	3	3	3	3	3	3	3
		N460K	G476S	S477N	T478K	E484A	Q498R	N501Y	Y505H	D614G	S640F	H655Y
		3	3	3	3	3	3	3	3	3	3	3
		N679K	P681H	N764K	D796Y	Q954H	N969K					
		3	3	3	3	3	3					
3	BA.2.3.20	T19I	L24-	P25-	P26-	A27S	G142D	M153T	N164K	V213G	H245N	G257D
		3	3	3	3	3	3	3	3	3	3	3
		G339D	S371F	S373P	S375F	T376A	D405N	R408S	K417N	N440K	K444R	N450D
		3	3	3	3	3	3	3	3	3	3	3
		L452M	N460K	S477N	T478K	E484R	Q498R	N501Y	Y505H	D614G	H655Y	N679K
		3	3	3	3	3	3	3	3	3	3	3
		P681H	N764K	D796Y	Q954H	N969K						
		3	3	3	3	3						
1	CM.10	T19I	L24-	P25-	P26-	A27S	G142D	M153T	N164K	V213G	H245N	G257D
		1	1	1	1	1	1	1	1	1	1	1
		G339D	S371F	S373P	S375F	T376A	D405N	R408S	K417N	N440K	K444R	V445A
		1	1	1	1	1	1	1	1	1	1	1
		N450D	L452M	N460K	S477N	E484R	Q498R	N501Y	Y505H	D614G	H655Y	N679K
		1	1	1	1	1	1	1	1	1	1	1
		P681H	N764K	D796Y	Q954H	N969K						
		1	1	1	1	1						

1	BA.2.75.2	T19I	L24-	P25-	P26-	A27S	G142D	K147E	W152R	F157L	I210V	V213G
		1	1	1	1	1	1	1	1	1	1	1
		G257S	G339H	R346T	S371F	S373P	S375F	T376A	D405N	R408S	K417N	N440K
		1	1	1	1	1	1	1	1	1	1	1
		G446S	N460K	S477N	T478K	E484A	F486S	Q498R	N501Y	Y505H	D614G	H655Y
		1	1	1	1	1	1	1	1	1	1	1
		N679K	P681H	N764K	D796Y	Q954H	N969K	D1199N				
		1	1	1	1	1	1	1				
1	CV.1	T19I	L24-	P25-	P26-	A27S	G142D	K147E	W152R	F157L	I210V	V213G
		1	1	1	1	1	1	1	1	1	1	1
		G257S	G339H	R346T	S371F	S373P	S375F	T376A	D405N	R408S	K417N	N440K
		1	1	1	1	1	1	1	1	1	1	1
		G446S	L452R	N460K	S477N	T478K	E484A	F486S	Q498R	N501Y	Y505H	D614G
		1	1	1	1	1	1	1	1	1	1	1
		H655Y	N679K	P681H	N764K	D796Y	Q954H	N969K				
		1	1	1	1	1	1	1				
16	CH.1.1	T19I	L24-	P25-	P26-	A27S	G142D	K147E	W152R	F157L	I210V	V213G
		16	16	16	16	16	16	16	16	16	16	16
		G257S	G339H	R346T	S371F	S373P	S375F	T376A	D405N	R408S	K417N	N440K
		16	16	16	16	16	16	16	16	16	16	16
		K444T	G446S	L452R	N460K	S477N	T478K	E484A	F486S	Q498R	N501Y	Y505H
		16	16	16	16	16	16	16	16	16	16	16
		D614G	H655Y	N679K	P681H	N764K	D796Y	Q954H	N969K			
		16	16	16	16	16	15	16	16			
3	CH.1.1.1	T19I	L24-	P25-	P26-	A27S	G142D	K147E	W152R	F157L	N185D	I210V
		3	3	3	3	3	3	3	3	3	3	3
		V213G	G257S	G339H	R346T	S371F	S373P	S375F	T376A	D405N	R408S	K417N
		3	3	3	3	3	3	3	3	3	3	3
		N440K	K444T	G446S	L452R	N460K	S477N	T478K	E484A	F486S	Q498R	N501Y
		3	3	3	3	3	3	2	3	3	3	3
		Y505H	D614G	H655Y	N679K	P681H	N764K	D796Y	Q954H	N969K		

		3	3	3	3	3	3	3	3	3		
5	DV.6	T19I	L24-	P25-	P26-	A27S	G142D	K147E	W152R	F157L	N185D	I210V
		5	5	5	5	5	5	5	5	5	5	5
		V213G	G257S	G339H	R346T	S371F	S373P	S375F	T376A	D405N	R408S	K417N
		5	5	4	5	5	5	5	5	5	5	5
		N440K	K444T	G446S	L452R	N460K	S477N	T478K	E484A	F486S	Q498R	N501Y
		5	5	5	5	5	5	5	5	5	5	5
		Y505H	D614G	H655Y	N679K	P681H	N764K	D796Y	Q954H	N969K		
		5	5	5	5	5	5	5	5	5		
1	DV.7.1.1	T19I	L24-	P25-	P26-	A27S	G142D	K147E	W152R	F157L	N185D	I210V
		1	1	1	1	1	1	1	1	1	1	1
		V213G	G257S	G339H	R346T	S371F	S373P	S375F	T376A	D405N	R408S	K417N
		1	1	1	1	1	1	1	1	1	1	1
		N440K	K444T	G446S	L452R	L455F	F456L	N460K	S477N	T478K	E484A	F486S
		1	1	1	1	1	1	1	1	1	1	1
		Q498R	N501Y	Y505H	D614G	H655Y	N679R	P681H	N764K	D796Y	L858I	Q954H
		1	1	1	1	1	1	1	1	1	1	1
		N969K										
		1										
2	DV.7.1.3	T19I	L24-	P25-	P26-	A27S	G142D	K147E	W152R	F157L	N185D	I210V
		2	2	2	2	2	2	2	2	2	2	2
		V213G	G257S	A262S	G339H	R346T	S371F	S373P	S375F	T376A	D405N	R408S
		2	2	2	2	2	2	2	2	2	2	2
		K417N	N440K	K444T	G446S	L452R	L455F	F456L	N460K	S477N	T478K	E484A
		2	2	2	2	2	2	2	2	2	2	2
		F486S	Q498R	N501Y	Y505H	D614G	H655Y	N679K	P681H	N764K	D796Y	L858I
		2	2	2	2	2	2	2	2	2	2	2
		Q954H	N969K									
		2	2									
8	DV.7.1.4	T19I	L24-	P25-	P26-	A27S	G142D	K147E	W152R	F157L	N185D	I210V
		8	8	8	8	8	8	8	8	8	8	8

		V213G	G257S	G339H	R346T	S371F	S373P	S375F	T376A	D405N	R408S	K417N
		8	8	8	8	7	7	7	7	8	8	8
		N440K	K444T	G446S	L452R	L455F	F456L	N460K	S477N	T478K	E484A	F486S
		8	8	8	8	8	8	8	8	8	8	8
		Q498R	N501Y	Y505H	D614G	H655Y	N679K	P681H	N764K	D796Y	L858I	Q954H
		8	8	8	8	7	8	8	8	8	8	8
		N969K										
		8										
1	DV.7.1.5	T19I	L24-	P25-	P26-	A27S	G142D	K147E	W152R	F157L	N185D	I210V
		1	1	1	1	1	1	1	1	1	1	1
		V213G	G257S	G339H	R346T	S371F	S373P	S375F	T376A	D405N	R408S	K417N
		1	1	1	1	1	1	1	1	1	1	1
		N440K	K444T	G446S	L452R	L455F	F456L	N460K	S477N	T478K	E484A	F486S
		1	1	1	1	1	1	1	1	1	1	1
		Q498R	N501Y	Y505H	D614G	H655Y	N679K	P681H	N764K	D796Y	L858I	Q954H
		1	1	1	1	1	1	1	1	1	1	1
		N969K										
		1										
2	CH.1.1.2	T19I	L24-	P25-	P26-	A27S	G142D	K147E	W152R	F157L	I210V	V213G
		2	2	2	2	2	2	2	2	2	2	2
		G257S	G339H	R346T	S371F	S373P	S375F	T376A	D405N	R408S	K417N	N440K
		2	2	2	2	2	2	2	2	2	2	2
		K444T	G446S	L452R	N460K	S477N	T478K	E484A	F486S	Q498R	N501Y	Y505H
		2	2	2	2	2	2	2	2	2	2	2
		D614G	H655Y	N679K	P681H	N764K	D796Y	T883I	Q954H	N969K		
		2	2	2	2	2	2	2	2	2		
1	GP.1	T19I	L24-	P25-	P26-	A27S	G142D	K147E	W152R	F157L	I210V	V213G
		1	1	1	1	1	1	1	1	1	1	1
		G257S	G339H	R346T	S371F	S373P	S375F	T376A	D405N	R408S	K417N	N440K
		1	1	1	1	1	1	1	1	1	1	1
		K444T	G446S	L452R	N460K	S477N	T478K	E484A	F486S	Q498R	N501Y	Y505H

		1	1	1	1	1	1	1	1	1	1	1
		D614G	H655Y	N679K	P681H	N764K	D796Y	Q954H	N969K			
		1	1	1	1	1	1	1	1			
4	BN.1	T19I	L24-	P25-	P26-	A27S	G142D	K147E	W152R	F157L	I210V	V213G
		4	4	4	4	4	4	4	4	4	4	4
		G257S	G339H	R346T	K356T	S371F	S373P	S375F	T376A	D405N	R408S	K417N
		4	4	4	4	4	4	4	4	4	4	4
		N440K	G446S	N460K	S477N	T478K	E484A	F490S	Q498R	N501Y	Y505H	D614G
		4	4	4	4	4	4	4	4	4	4	4
		H655Y	N679K	P681H	N764K	D796Y	Q954H	N969K				
		4	4	4	4	4	4	4				
3	BN.1.1.1	T19I	L24-	P25-	P26-	A27S	G142D	K147E	W152R	F157L	N185D	I210V
		3	3	3	3	3	3	3	3	3	3	3
		V213G	G257S	G339H	R346T	K356T	S371F	S373P	S375F	T376A	D405N	R408S
		3	3	3	3	3	3	3	3	3	3	3
		K417N	N440K	G446S	N460K	S477N	T478K	E484A	F490S	S494P	Q498R	N501Y
		3	3	3	3	3	3	3	3	3	3	3
		Y505H	D614G	H655Y	N679K	P681H	N764K	D796Y	Q954H	N969K		
		3	3	3	3	3	3	3	3	3		
2	FR.1	T19I	L24-	P25-	P26-	A27S	G142D	K147E	W152R	F157L	K187E	I210V
		2	2	2	2	2	2	2	2	2	2	2
		V213G	G257S	G339H	R346T	K356T	S371F	S373P	S375F	T376A	D405N	R408S
		2	2	2	2	2	2	2	2	2	2	2
		K417N	N440K	G446S	N460K	S477N	T478K	E484A	F490S	Q498R	N501Y	Y505H
		2	2	2	2	2	2	2	2	2	2	2
		D614G	H655Y	N679K	P681H	N764K	D796Y	Q954H	N969K			
		2	2	2	2	2	2	2	2			
29	BN.1.3	T19I	L24-	P25-	P26-	A27S	G142D	K147E	W152R	F157L	I210V	V213G
		29	29	29	29	29	29	29	29	29	29	29
		G257S	G339H	R346T	K356T	S371F	S373P	S375F	T376A	D405N	R408S	K417N
		29	29	29	29	29	29	29	29	29	29	29

		N440K	G446S	N460K	S477N	T478K	E484A	F490S	Q498R	N501Y	Y505H	D614G
		29	29	29	29	29	29	29	29	29	29	29
		H655Y	N679K	P681H	N764K	D796Y	Q954H	N969K				
		29	29	29	29	29	29	29				
9	BN.1.3.1	T19I	L24-	P25-	P26-	A27S	G142D	K147E	W152R	F157L	I210V	V213G
		9	9	9	9	9	9	9	9	9	9	9
		G257S	G339H	R346T	K356T	S371F	S373P	S375F	T376A	D405N	R408S	K417N
		9	9	9	9	9	9	9	9	9	9	9
		N440K	G446S	N460K	S477N	T478K	E484A	F490S	Q498R	N501Y	Y505H	D614G
		9	9	9	9	9	9	9	9	9	9	9
		H655Y	N679K	P681H	N764K	D796Y	Q954H	N969K				
		9	9	9	9	9	9	9				
5	BN.1.5	T19I	L24-	P25-	P26-	A27S	G142D	K147E	W152R	F157L	I210V	V213G
		5	5	5	5	5	5	5	5	5	5	5
		G257S	G339H	R346T	K356T	S371F	S373P	S375F	T376A	D405N	R408S	K417N
		5	5	5	5	5	5	5	5	5	5	5
		N440K	G446S	N460K	S477N	T478K	E484A	F490S	Q498R	N501Y	Y505H	D614G
		5	5	5	5	5	5	5	5	5	5	5
		H655Y	N679K	P681H	N764K	D796Y	Q954H	N969K				
		5	5	5	5	5	5	5				
4	BN.3.1	T19I	L24-	P25-	P26-	A27S	G142D	K147E	W152R	F157L	I210V	V213G
		4	4	4	4	4	4	4	4	4	4	4
		G257S	G339H	K356T	S371F	S373P	S375F	T376A	D405N	R408S	K417N	N440K
		4	4	4	4	4	4	4	4	4	4	4
		G446S	N450D	N460K	S477N	T478K	E484A	F490S	Q498R	N501Y	Y505H	D614G
		4	4	4	4	4	4	4	4	4	4	4
		H655Y	N679K	P681H	N764K	D796Y	Q954H	N969K				
		4	4	4	4	4	4	4				
1	BN.1.10	T19I	L24-	P25-	P26-	A27S	G142D	Y145H	K147E	W152R	F157L	I210V
		1	1	1	1	1	1	1	1	1	1	1
		V213G	G257S	G339H	R346T	K356T	S371F	S373P	S375F	T376A	D405N	R408S

		1	1	1	1	1	1	1	1	1	1	1
		K417N	N440K	G446S	N460K	S477N	T478K	E484A	F490S	Q498R	N501Y	Y505H
		1	1	1	1	1	1	1	1	1	1	1
		D614G	H655Y	N679K	P681H	N764K	D796Y	Q954H	N969K			
		1	1	1	1	1	1	1	1			
2	BR.2.1	T19I	L24-	P25-	P26-	A27S	G142D	K147E	W152R	F157L	I210V	V213G
		2	2	2	2	2	2	2	2	2	2	2
		G257S	G339H	R346T	S371F	S373P	S375F	T376A	D405N	R408S	K417N	N440K
		2	2	2	2	2	2	2	2	2	2	2
		G446S	L452R	N460K	S477N	T478K	E484A	F486I	Q498R	N501Y	Y505H	D614G
		2	2	2	2	2	2	2	2	2	2	2
		H655Y	N679K	P681H	N764K	D796Y	Q954H	N969K				
		2	2	2	2	2	2	2				
17	BR.3	T19I	L24-	P25-	P26-	A27S	G142D	K147E	W152R	F157L	I210V	V213G
		2	2	2	2	2	2	2	2	2	2	2
		G257S	G339H	R346T	S371F	S373P	S375F	T376A	D405N	R408S	K417N	N440K
		2	2	2	2	2	2	2	2	2	2	2
		G446S	L452R	N460K	S477N	T478K	E484A	Q498R	N501Y	Y505H	D614G	H655Y
		2	2	2	2	2	2	2	2	2	2	2
		N679K	P681H	N764K	D796Y	Q954H	N969K					
		2	2	2	2	2	2					
1	BA.2.86	T19I	R21T	L24-	P25-	P26-	A27S	S50L	H69-	V70-	V127F	G142D
		1	1	1	1	1	1	1	1	1	1	1
		Y144-	F157S	R158G	N211-	L212I	V213G	L216F	H245N	A264D	I332V	G339H
		1	1	1	1	1	1	1	1	1	1	1
		K356T	S371F	S373P	S375F	T376A	R403K	D405N	R408S	K417N	N440K	V445H
		1	1	1	1	1	1	1	1	1	1	1
		G446S	N450D	L452W	N460K	S477N	T478K	N481K	V483-	E484K	F486P	Q498R
		1	1	1	1	1	1	1	1	1	1	1
		N501Y	Y505H	E554K	A570V	D614G	P621S	H655Y	N679K	P681R	N764K	D796Y

		1	1	1	1	1	1	1	1	1	1	1
		S939F	Q954H	N969K	P1143L							
		1	1	1	1							
26	BA.2.86.1	T19I	R21T	L24-	P25-	P26-	A27S	S50L	H69-	V70-	V127F	G142D
		19	19	19	19	19	19	26	26	26	26	26
		Y144-	F157S	R158G	N211-	L212I	V213G	L216F	H245N	A264D	I332V	G339H
		26	26	26	26	26	26	26	26	26	26	26
		K356T	S371F	S373P	S375F	T376A	R403K	D405N	R408S	K417N	N440K	V445H
		26	26	26	26	26	26	26	26	26	26	26
		G446S	N450D	L452W	N460K	S477N	T478K	N481K	V483-	E484K	F486P	Q498R
		26	26	26	26	26	26	26	26	26	26	26
		N501Y	Y505H	E554K	A570V	D614G	P621S	H655Y	N679K	P681R	N764K	D796Y
		26	26	26	26	26	25	26	26	25	26	25
		S939F	Q954H	N969K	P1143L							
		26	26	25	26							
188	JN.1	T19I	R21T	L24-	P25-	P26-	A27S	S50L	H69-	V70-	V127F	G142D
		138	132	138	138	138	141	176	181	181	188	188
		Y144-	F157S	R158G	N211-	L212I	V213G	L216F	H245N	A264D	I332V	G339H
		188	188	188	188	188	188	188	188	188	187	187
		K356T	S371F	S373P	S375F	T376A	R403K	D405N	R408S	K417N	N440K	V445H
		187	188	188	187	187	185	188	188	188	185	185
		G446S	N450D	L452W	L455S	N460K	S477N	T478K	N481K	V483-	E484K	F486P
		185	185	185	185	185	185	185	183	182	182	184
		Q498R	N501Y	Y505H	E554K	A570V	D614G	P621S	H655Y	N679K	P681R	N764K
		185	185	185	188	188	188	188	185	188	188	188
		D796Y	S939F	Q954H	N969K	P1143L						
		188	187	187	188	187						
66	JN.1.1	ins16MPLF	T19I	R21T	L24-	P25-	P26-	A27S	S50L	H69-	V70-	V127F
		5	48	46	48	48	48	50	56	65	65	66
		G142D	Y144-	F157S	R158G	N211-	L212I	V213G	L216F	H245N	A264D	I332V
		66	66	66	66	66	66	66	66	66	66	66

		G339H	K356T	S371F	S373P	S375F	T376A	R403K	D405N	R408S	K417N	N440K
		66	66	66	66	66	66	64	66	66	66	66
		V445H	G446S	N450D	L452W	L455S	N460K	S477N	T478K	N481K	V483-	E484K
		66	66	66	66	66	66	66	66	58	58	66
		F486P	Q498R	N501Y	Y505H	E554K	A570V	D614G	P621S	H655Y	N679K	P681R
		66	66	66	66	66	66	65	66	65	66	66
		N764K	D796Y	S939F	Q954H	N969K	P1143L					
		65	66	66	66	66	66					
2	JN.1.1.1	T19I	R21T	L24-	P25-	P26-	A27S	S50L	H69-	V70-	V127F	G142D
		1	1	1	1	1	1	2	2	2	2	2
		Y144-	F157S	R158G	N211-	L212I	V213G	L216F	H245N	A264D	I332V	G339H
		2	2	2	2	2	2	2	2	2	2	2
		K356T	S371F	S373P	S375F	T376A	R403K	D405N	R408S	K417N	N440K	V445H
		2	2	2	2	2	2	2	2	2	2	2
		G446S	N450D	L452W	L455S	N460K	S477N	T478K	N481K	V483-	E484K	F486P
		2	2	2	2	2	2	2	2	2	2	2
		Q498R	N501Y	Y505H	E554K	A570V	T572I	D614G	P621S	H655Y	N679K	P681R
		2	2	2	2	2	2	2	2	2	2	2
		N764K	D796Y	S939F	Q954H	N969K	P1143L					
		2	2	2	2	2	2					
6	JN.1.2	T19I	R21T	L24-	P25-	P26-	A27S	S50L	H69-	V70-	V127F	G142D
		6	6	6	6	6	6	6	6	6	6	6
		Y144-	F157S	R158G	N211-	L212I	V213G	L216F	H245N	A264D	I332V	G339H
		6	6	6	6	6	6	6	6	6	6	6
		K356T	S371F	S373P	S375F	T376A	R403K	D405N	R408S	K417N	N440K	V445H
		6	6	6	6	6	6	6	6	6	6	6
		G446S	N450D	L452W	L455S	N460K	S477N	T478K	N481K	V483-	E484K	F486P
		6	6	6	6	6	6	6	6	6	6	6
		Q498R	N501Y	Y505H	E554K	A570V	D614G	P621S	H655Y	N679K	P681R	N764K
		6	6	6	6	6	6	6	5	6	6	6
		D796Y	S939F	Q954H	N969K	P1143L	M1229I					

		6	6	6	6	6	6					
60	JN.1.4	T19I	R21T	L24-	P25-	P26-	A27S	S50L	H69-	V70-	V127F	G142D
		39	39	39	39	39	39	57	59	59	60	60
		Y144-	F157S	R158G	N211-	L212I	V213G	L216F	H245N	A264D	I332V	G339H
		60	60	60	60	60	60	60	60	60	60	60
		K356T	S371F	S373P	S375F	T376A	R403K	D405N	R408S	K417N	N440K	V445H
		60	60	60	60	59	59	60	60	60	60	60
		G446S	N450D	L452W	L455S	N460K	S477N	T478K	N481K	V483-	E484K	F486P
		60	60	60	60	60	60	60	59	59	59	59
		Q498R	N501Y	Y505H	E554K	A570V	D614G	P621S	H655Y	N679K	P681R	N764K
		60	60	60	60	60	60	60	60	60	60	60
		D796Y	S939F	Q954H	N969K	P1143L						
		60	59	59	60	59						
1	JN.1.5	T19I	R21T	L24-	P25-	P26-	A27S	S50L	H69-	V70-	V127F	G142D
		1	1	1	1	1	1	1	1	1	1	1
		Y144-	F157S	R158G	N211-	L212I	V213G	L216F	H245N	A264D	I332V	G339H
		1	1	1	1	1	1	1	1	1	1	1
		K356T	S371F	S373P	S375F	T376A	R403K	D405N	R408S	K417N	N440K	V445H
		1	1	1	1	1	1	1	1	1	1	1
		G446S	N450D	L452W	L455S	N460K	S477N	T478K	N481K	F486P	Q498R	N501Y
		1	1	1	1	1	1	1	1	1	1	1
		Y505H	E554K	A570V	D614G	P621S	H655Y	N679K	P681R	N764K	D796Y	S939F
		1	1	1	1	1	1	1	1	1	1	1
		Q954H	N969K	P1143L								
		1	1	1								
1	JN.1.7	T19I	L24-	P25-	P26-	A27S	S50L	H69-	V70-	V127F	G142D	Y144-
		1	1	1	1	1	1	1	1	1	1	1
		F157S	R158G	N211-	L212I	V213G	L216F	H245N	A264D	I332V	G339H	K356T
		1	1	1	1	1	1	1	1	1	1	1
		S371F	S373P	S375F	T376A	R403K	D405N	R408S	K417N	N440K	V445H	G446S
		1	1	1	1	1	1	1	1	1	1	1

		N450D	L452W	L455S	N460K	S477N	T478K	N481K	V483-	E484K	F486P	Q498R
		1	1	1	1	1	1	1	1	1	1	1
		N501Y	Y505H	E554K	A570V	T572I	D614G	P621S	H655Y	N679K	P681R	N764K
		1	1	1	1	1	1	1	1	1	1	1
		D796Y	S939F	Q954H	N969K	P1143L	E1150D					
		1	1	1	1	1	1					
		T19I	R21T	L24-	P25-	P26-	A27S	S50L	H69-	V70-	V127F	G142D
		1	1	1	1	1	1	1	1	1	1	1
		Y144-	F157S	R158G	N211-	L212I	V213G	L216F	H245N	A264D	I332V	G339H
		1	1	1	1	1	1	1	1	1	1	1
		K356T	S371F	S373P	S375F	T376A	R403K	D405N	R408S	K417N	N440K	V445H
		1	1	1	1	1	1	1	1	1	1	1
1	JN.1.8	G446S	N450D	L452W	L455S	N460K	S477N	T478K	N481K	V483-	E484K	F486P
		1	1	1	1	1	1	1	1	1	1	1
		Q498R	N501Y	Y505H	E554K	A570V	D614G	P621S	H655Y	N679K	P681R	N764K
		1	1	1	1	1	1	1	1	1	1	1
		D796Y	S939F	Q954H	N969K	P1143L						
		1	1	1	1	1						
		S50L	H69-	V70-	V127F	G142D	Y144-	F157S	R158G	Q183H	N211-	L212I
		1	1	1	1	1	1	1	1	1	1	1
		V213G	L216F	H245N	A264D	I332V	G339H	K356T	S371F	S373P	S375F	T376A
		1	1	1	1	1	1	1	1	1	1	1
1	JN.1.9	R403K	D405N	R408S	K417N	N440K	V445H	G446S	N450D	L452W	L455S	N460K
		1	1	1	1	1	1	1	1	1	1	1
		S477N	T478K	N481K	V483-	E484K	F486P	Q498R	N501Y	Y505H	E554K	A570V
		1	1	1	1	1	1	1	1	1	1	1
		D614G	P621S	H655Y	N679K	P681R	N764K	D796Y	S939F	Q954H	N969K	P1143L
		1	1	1	1	1	1	1	1	1	1	1
		T19I	R21T	L24-	P25-	P26-	A27S	S50L	H69-	V70-	V127F	G142D
3	JN.1.16	2	1	2	2	2	2	3	3	3	3	3
		Y144-	F157S	R158G	N211-	L212I	V213G	L216F	H245N	A264D	I332V	G339H

		3	3	3	3	3	3	3	3	3	3	3
		K356T	S371F	S373P	S375F	T376A	R403K	D405N	R408S	K417N	N440K	V445H
		3	3	3	3	3	3	3	3	3	3	3
		G446S	N450D	L452W	L455S	F456L	N460K	S477N	T478K	N481K	V483-	E484K
		3	3	3	3	3	3	3	3	3	3	3
		F486P	Q498R	N501Y	Y505H	E554K	A570V	D614G	P621S	H655Y	N679K	P681R
		3	3	3	3	3	3	3	3	3	3	3
		N764K	D796Y	S939F	Q954H	N969K	P1143L					
		3	3	3	3	3	3					
1	JN.1.18	S50L	H69-	V70-	V127F	G142D	Y144-	F157S	R158G	N211-	L212I	V213G
		1	1	1	1	1	1	1	1	1	1	1
		L216F	H245N	A264D	I332V	G339H	R346T	K356T	S371F	S373P	S375F	T376A
		1	1	1	1	1	1	1	1	1	1	1
		R403K	D405N	R408S	K417N	N440K	V445H	G446S	N450D	L452W	L455S	N460K
		1	1	1	1	1	1	1	1	1	1	1
		S477N	T478K	N481K	V483-	E484K	F486P	Q498R	N501Y	Y505H	E554K	A570V
		1	1	1	1	1	1	1	1	1	1	1
		D614G	P621S	H655Y	N679K	P681R	N764K	D796Y	S939F	Q954H	N969K	P1143L
		1	1	1	1	1	1	1	1	1	1	1
1	JN.1.22	S50L	H69-	V70-	V127F	G142D	Y144-	F157S	R158G	N211-	L212I	V213G
		1	1	1	1	1	1	1	1	1	1	1
		L216F	H245N	A264D	I332V	G339H	K356T	S371F	S373P	S375F	T376A	R403K
		1	1	1	1	1	1	1	1	1	1	1
		D405N	R408S	K417N	N440K	V445H	G446S	N450D	L452W	L455S	N460K	S477N
		1	1	1	1	1	1	1	1	1	1	1
		T478K	N481K	V483-	E484K	F486P	Q498R	N501Y	Y505H	E554K	A570V	D614G
		1	1	1	1	1	1	1	1	1	1	1
		P621S	H655Y	N679K	P681R	N764K	D796Y	S939F	Q954H	N969K	P1143L	
		1	1	1	1	1	1	1	1	1	1	
10	JN.2	T19I	R21T	L24-	P25-	P26-	A27S	S50L	H69-	V70-	V127F	G142D
		10	10	10	10	10	10	9	10	10	10	10

		Y144-	F157S	R158G	N211-	L212I	V213G	L216F	H245N	A264D	I332V	G339H
		10	10	10	10	10	10	10	10	10	10	10
		K356T	S371F	S373P	S375F	T376A	R403K	D405N	R408S	K417N	N440K	V445H
		10	10	10	10	10	10	10	10	10	10	10
		G446S	N450D	L452W	N460K	S477N	T478K	N481K	V483-	E484K	F486P	Q498R
		10	10	10	10	10	10	9	10	10	10	10
		N501Y	Y505H	E554K	A570V	D614G	P621S	H655Y	N679K	P681R	N764K	D796Y
		10	10	10	10	10	10	10	10	10	10	10
		S939F	Q954H	N969K	P1143L							
		10	10	10	10							
6	JN.3	T19I	R21T	L24-	P25-	P26-	A27S	S50L	H69-	V70-	V127F	G142D
		6	6	6	6	6	6	5	5	5	6	6
		Y144-	F157S	R158G	N211-	L212I	V213G	L216F	H245N	A264D	I332V	G339H
		6	6	6	6	6	6	6	6	6	6	6
		K356T	S371F	S373P	S375F	T376A	R403K	D405N	R408S	K417N	N440K	V445H
		6	6	6	6	6	6	6	6	6	6	6
		G446S	N450D	L452W	N460K	S477N	T478K	N481K	V483-	E484K	F486P	Q498R
		6	6	6	6	6	6	6	6	6	6	6
		N501Y	Y505H	E554K	A570V	D614G	P621S	H655Y	N679K	P681R	N764K	D796Y
		6	6	6	6	6	6	6	6	6	6	6
		S939F	Q954H	N969K	P1143L							
		6	6	6	6							
2	JN.5	T19I	R21T	L24-	P25-	P26-	A27S	S50L	H69-	V70-	V127F	G142D
		2	2	2	2	2	2	2	2	2	2	2
		Y144-	F157S	R158G	N211-	L212I	V213G	L216F	H245N	A264D	I332V	G339H
		2	2	2	2	2	2	2	2	2	2	2
		K356T	S371F	S373P	S375F	T376A	R403K	D405N	R408S	K417N	N440K	V445H
		2	2	2	2	2	2	2	2	2	2	2
		G446S	N450D	L452W	N460K	S477N	T478K	N481K	V483-	E484K	F486P	Q498R
		2	2	2	2	2	2	2	2	2	2	2
		N501Y	Y505H	E554K	A570V	D614G	P621S	H655Y	N679K	P681R	N764K	D796Y

		2	2	2	2	2	2	2	2	2	2	2
		S939F	Q954H	N969K	P1143L							
		2	2	2	2							
4	JN.6	T19I	R21T	L24-	P25-	P26-	A27S	S50L	H69-	V70-	V127F	G142D
		4	4	4	4	4	4	4	4	4	4	4
		Y144-	F157S	R158G	V193L	N211-	L212I	V213G	L216F	H245N	A264D	I332V
		4	4	4	4	4	4	4	4	4	4	4
		G339H	K356T	S371F	S373P	S375F	T376A	R403K	D405N	R408S	K417N	N440K
		4	4	4	4	4	4	4	4	4	4	4
		V445H	G446S	N450D	L452W	N460K	S477N	T478K	N481K	V483-	E484K	F486P
		4	4	4	4	4	4	4	4	4	4	4
		Q498R	N501Y	Y505H	E554K	A570V	D614G	P621S	H655Y	N679K	P681R	N764K
		4	4	4	4	4	4	4	4	4	4	4
		D796Y	S939F	Q954H	N969K	P1143L						
		4	4	4	4	4						
8	JN.9	ins16MPLF	T19I	R21T	L24-	P25-	P26-	A27S	S50L	H69-	V70-	V127F
		1	8	7	8	8	8	8	8	8	8	8
		G142D	Y144-	F157S	R158G	N211-	L212I	V213G	L216F	H245N	A264D	I332V
		8	8	8	8	8	8	8	8	8	8	8
		G339H	K356T	S371F	S373P	S375F	T376A	R403K	D405N	R408S	K417N	N440K
		8	8	8	8	8	8	8	8	8	8	8
		V445H	G446S	N450D	L452W	N460K	S477N	T478K	N481K	V483-	E484K	F486P
		8	8	8	8	8	8	8	8	8	8	8
		Q498R	N501Y	Y505H	E554K	A570V	D614G	P621S	H655Y	N679K	P681R	N764K
		8	8	8	8	8	8	8	8	8	8	8
		D796Y	S939F	Q954H	N969K	P1143L	M1229I					
		8	8	8	8	8	8					
28	JN.10	T19I	R21T	L24-	P25-	P26-	A27S	S50L	H69-	V70-	V127F	G142D
		26	25	26	26	26	26	27	28	28	28	28
		Y144-	F157S	R158G	N211-	L212I	V213G	L216F	H245N	A264D	I332V	G339H
		28	28	28	28	28	28	28	28	28	28	28

		K356T	S371F	S373P	S375F	T376A	R403K	D405N	R408S	K417N	N440K	V445H
		28	28	28	28	28	24	28	28	28	28	28
		G446S	N450D	L452W	N460K	A475V	S477N	T478K	N481K	V483-	E484K	F486P
		28	28	28	28	28	28	28	28	28	28	28
		Q498R	N501Y	Y505H	E554K	A570V	D614G	P621S	H655Y	N679K	P681R	N764K
		28	28	28	28	28	28	28	27	28	28	28
		D796Y	S939F	Q954H	N969K	P1143L						
		28	28	27	27	28						
1	BA.4.6	T19I	L24-	P25-	P26-	A27S	H69-	V70-	G142D	V213G	G339D	R346T
		1	1	1	1	1	1	1	1	1	1	1
		S371F	S373P	S375F	T376A	D405N	R408S	K417N	N440K	L452R	S477N	T478K
		1	1	1	1	1	1	1	1	1	1	1
		E484A	F486V	Q498R	N501Y	Y505H	D614G	H655Y	N658S	N679K	P681H	N764K
		1	1	1	1	1	1	1	1	1	1	1
		D796Y	Q954H	N969K								
		1	1	1								
4	BA.5.1	T19I	L24-	P25-	P26-	A27S	H69-	V70-	G142D	V213G	G339D	S371F
		4	4	4	4	4	4	4	4	4	4	4
		S373P	S375F	T376A	D405N	R408S	K417N	N440K	L452R	S477N	T478K	E484A
		4	4	4	4	4	4	4	4	4	4	4
		F486V	Q498R	N501Y	Y505H	D614G	H655Y	N679K	P681H	N764K	D796Y	Q954H
		4	4	4	4	4	4	4	4	4	4	4
		N969K										
		4										
2	BA.5.1.5	T19I	L24-	P25-	P26-	A27S	H69-	V70-	G142D	V213G	G339D	S371F
		2	2	2	2	2	2	2	2	2	2	2
		S373P	S375F	T376A	D405N	R408S	K417N	N440K	L452R	S477N	T478K	E484A
		2	2	2	2	2	2	2	2	2	2	2
		F486V	Q498R	N501Y	Y505H	D614G	H655Y	N679K	P681H	N764K	D796Y	Q954H
		2	2	2	2	2	2	2	2	2	2	2
		N969K	P1263Q									

		2	2									
4	BA.5.1.10	T19I	L24-	P25-	P26-	A27S	H69-	V70-	G142D	V213G	G339D	S371F
		4	4	4	4	4	4	4	4	4	4	4
		S373P	S375F	T376A	D405N	R408S	K417N	N440K	L452R	S477N	T478K	E484A
		4	4	4	4	4	4	4	4	4	4	4
		F486V	Q498R	N501Y	Y505H	D614G	H655Y	N679K	P681H	N764K	D796Y	Q954H
		4	4	4	4	4	4	4	4	4	4	4
		N969K										
		4										
16	BT.2	T19I	L24-	P25-	P26-	A27S	H69-	V70-	G142D	V213G	G339D	S371F
		16	16	16	16	16	16	16	16	16	16	16
		S373P	S375F	T376A	D405N	R408S	K417N	N440K	L452R	S477N	T478K	E484A
		16	16	16	16	16	16	16	16	16	16	16
		F486V	Q498R	N501Y	Y505H	D614G	H655Y	N679K	P681H	N764K	D796Y	Q954H
		16	16	16	16	16	16	16	16	16	16	16
		N969K										
		16										
13	BA.5.2	T19I	L24-	P25-	P26-	A27S	H69-	V70-	G142D	V213G	G339D	S371F
		13	13	13	13	13	13	13	13	13	13	13
		S373P	S375F	T376A	D405N	R408S	K417N	N440K	L452R	S477N	T478K	E484A
		13	13	13	13	13	13	13	13	13	13	13
		F486V	Q498R	N501Y	Y505H	D614G	H655Y	N679K	P681H	N764K	D796Y	Q954H
		13	13	13	13	13	13	13	13	13	13	13
		N969K										
		13										
20	BA.5.2.1	T19I	L24-	P25-	P26-	A27S	H69-	V70-	G142D	V213G	G339D	S371F
		20	20	20	20	20	19	19	20	20	20	20
		S373P	S375F	T376A	D405N	R408S	K417N	N440K	L452R	S477N	T478K	E484A
		20	20	20	20	20	20	20	20	20	20	20
		F486V	Q498R	N501Y	Y505H	D614G	H655Y	N679K	P681H	N764K	D796Y	Q954H
		20	20	20	20	20	20	20	20	20	20	20

		N969K										
		20										
1	BF.1	T19I	L24-	P25-	P26-	A27S	H69-	V70-	G142D	V213G	G339D	S371F
		1	1	1	1	1	1	1	1	1	1	1
		S373P	S375F	T376A	D405N	R408S	K417N	N440K	L452R	S477N	T478K	E484A
		1	1	1	1	1	1	1	1	1	1	1
		F486V	Q498R	N501Y	Y505H	D614G	H655Y	N679K	P681H	N764K	D796Y	Q954H
		1	1	1	1	1	1	1	1	1	1	1
		N969K										
		1										
13	BF.5	T19I	L24-	P25-	P26-	A27S	H69-	V70-	G142D	V213G	G339D	S371F
		13	13	13	13	13	13	13	13	13	13	13
		S373P	S375F	T376A	D405N	R408S	K417N	N440K	L452R	S477N	T478K	E484A
		13	13	13	13	13	13	13	13	13	13	13
		F486V	Q498R	N501Y	Y505H	D614G	H655Y	N679K	P681H	N764K	D796Y	Q954H
		13	13	13	13	13	13	13	13	13	13	13
		N969K	A1020S									
		13	13									
16	BF.7	T19I	L24-	P25-	P26-	A27S	H69-	V70-	G142D	V213G	G339D	R346T
		16	16	16	16	16	15	15	16	16	16	16
		S371F	S373P	S375F	T376A	D405N	R408S	K417N	N440K	L452R	S477N	T478K
		16	16	16	16	16	16	16	16	16	16	16
		E484A	F486V	Q498R	N501Y	Y505H	D614G	H655Y	N679K	P681H	N764K	D796Y
		16	16	16	16	16	16	16	16	16	16	16
		Q954H	N969K									
		16	16									
9	BF.7.1	T19I	L24-	P25-	P26-	A27S	H69-	V70-	G142D	V213G	G261V	G339D
		9	9	9	9	9	9	9	9	9	9	9
		R346T	S371F	S373P	S375F	T376A	D405N	R408S	K417N	N440K	L452R	S477N
		9	9	9	9	9	9	9	9	9	9	9
		T478K	E484A	F486V	Q498R	N501Y	Y505H	D614G	H655Y	N679K	P681H	N764K

		9	9	9	9	9	9	9	9	9	9	9
		D796Y	Q954H	N969K								
		9	9	9								
6	BF.7.4.1	T19I	L24-	P25-	P26-	A27S	H69-	V70-	G142D	V213G	G339D	R346T
		6	6	6	6	6	6	6	6	6	6	6
		S371F	S373P	S375F	T376A	D405N	R408S	K417N	N440K	L452R	S477N	T478K
		6	6	6	6	6	6	6	6	6	6	6
		E484A	F486V	Q498R	N501Y	Y505H	D614G	H655Y	N679K	P681H	N764K	D796Y
		6	6	6	6	6	6	6	6	6	6	6
		Q954H	N969K									
		6	6									
1	BF.7.6	T19I	L24-	P25-	P26-	A27S	H69-	V70-	G142D	V213G	G339D	R346T
		1	1	1	1	1	1	1	1	1	1	1
		S371F	S373P	S375F	T376A	D405N	R408S	K417N	N440K	L452R	S477N	T478K
		1	1	1	1	1	1	1	1	1	1	1
		E484A	F486V	Q498R	N501Y	Y505H	D614G	H655Y	N679K	P681H	N764K	D796Y
		1	1	1	1	1	1	1	1	1	1	1
		Q954H	N969K									
		1	1									
2	BF.7.7	T19I	L24-	P25-	P26-	A27S	H69-	V70-	D80G	G142D	V213G	G339D
		2	2	2	2	2	2	2	2	2	2	2
		R346T	S371F	S373P	S375F	T376A	D405N	R408S	K417N	N440K	L452R	S477N
		2	2	2	2	2	2	2	2	2	2	2
		T478K	E484A	F486V	Q498R	N501Y	Y505H	D614G	H655Y	N679K	P681H	N764K
		2	2	2	2	2	2	2	2	2	2	2
		D796Y	Q954H	N969K								
		2	2	2								
6	BF.7.8	T19I	L24-	P25-	P26-	A27S	H69-	V70-	G142D	V213G	G339D	R346T
		6	6	6	6	6	6	6	6	6	6	6
		S371F	S373P	S375F	T376A	D405N	R408S	K417N	N440K	L452R	S477N	T478K
		6	6	6	6	6	6	6	6	6	6	6

		E484A	F486V	Q498R	N501Y	Y505H	D614G	H655Y	N679K	P681H	N764K	D796Y
		6	6	6	6	6	6	6	6	6	6	6
		T883I	Q954H	N969K								
		6	6	6								
1	BF.7.13.2	T19I	L24-	P25-	P26-	A27S	H69-	V70-	G142D	V213G	G339D	R346T
		1	1	1	1	1	1	1	1	1	1	1
		S371F	S373P	S375F	T376A	D405N	R408S	K417N	N440K	L452R	S477N	T478K
		1	1	1	1	1	1	1	1	1	1	1
		E484A	F486V	Q498R	N501Y	Y505H	D614G	A653V	H655Y	N679K	P681H	N764K
		1	1	1	1	1	1	1	1	1	1	1
		D796Y	Q954H	N969K								
		1	1	1								
1	BF.7.20	T19I	L24-	P25-	P26-	A27S	H69-	G142D	V213G	G339D	R346T	S371F
		1	1	1	1	1	1	1	1	1	1	1
		S373P	S375F	T376A	D405N	R408S	K417N	N440K	L452R	S477N	T478K	E484A
		1	1	1	1	1	1	1	1	1	1	1
		F486V	Q498R	N501Y	Y505H	D614G	H655Y	N679K	P681H	N764K	D796Y	Q954H
		1	1	1	1	1	1	1	1	1	1	1
		N969K										
		1										
1	BF.7.22	T19I	L24-	P25-	P26-	A27S	H69-	V70-	G142D	V213G	G339D	R346T
		1	1	1	1	1	1	1	1	1	1	1
		S371F	S373P	S375F	T376A	D405N	R408S	K417N	N440K	L452R	S477N	T478K
		1	1	1	1	1	1	1	1	1	1	1
		E484A	F486V	Q498R	N501Y	Y505H	D614G	H655Y	N679K	P681H	N764K	D796Y
		1	1	1	1	1	1	1	1	1	1	1
		Q954H	N969K									
		1	1									
2	BF.7.23	T19I	L24-	P25-	P26-	A27S	H69-	V70-	G142D	V213G	G339D	R346T
		2	2	2	2	2	2	2	2	2	2	2
		S371F	S373P	S375F	T376A	D405N	R408S	K417N	N440K	L452R	S477N	T478K

		2	2	2	2	2	2	2	2	2	2	2
		E484A	F486V	Q498R	N501Y	Y505H	D614G	H655Y	N679K	P681H	N764K	D796Y
		2	2	2	2	2	2	2	2	2	2	2
		Q954H	N969K									
		2	2									
1	BF.7.24	T19I	L24-	P25-	P26-	A27S	H69-	V70-	G142D	V213G	G339D	R346T
		1	1	1	1	1	1	1	1	1	1	1
		S371F	S373P	S375F	T376A	D405N	R408S	K417N	N440K	L452R	S477N	T478K
		1	1	1	1	1	1	1	1	1	1	1
		E484A	F486V	Q498R	N501Y	Y505H	D614G	H655Y	N679K	P681H	N764K	D796Y
		1	1	1	1	1	1	1	1	1	1	1
		Q954H	N969K									
		1	1									
1	BF.10	T19I	L24-	P25-	P26-	A27S	H69-	V70-	G142D	V213G	G339D	S371F
		1	1	1	1	1	1	1	1	1	1	1
		S373P	S375F	T376A	D405N	R408S	K417N	N440K	L452R	S477N	T478K	E484A
		1	1	1	1	1	1	1	1	1	1	1
		F486V	Q498R	N501Y	Y505H	D614G	H655Y	N679K	P681H	N764K	D796Y	Q954H
		1	1	1	1	1	1	1	1	1	1	1
		N969K										
		1										
17	BF.11	T19I	L24-	P25-	P26-	A27S	H69-	V70-	G142D	V213G	G339D	R346T
		17	17	17	17	17	16	16	17	17	17	17
		S371F	S373P	S375F	T376A	D405N	R408S	K417N	N440K	L452R	S477N	T478K
		17	17	17	17	17	17	17	17	17	17	17
		E484A	F486V	Q498R	N501Y	Y505H	D614G	H655Y	N679K	P681H	N764K	D796Y
		17	17	17	17	17	17	17	17	17	17	17
		Q954H	N969K									
		17	17									
14	BF.11.2	T19I	L24-	P25-	P26-	A27S	H69-	V70-	G142D	K147N	V213G	G339D
		14	14	14	14	14	14	14	14	14	14	14

		R346T	S371F	S373P	S375F	T376A	D405N	R408S	K417N	N440K	L452R	S477N
		14	14	14	14	14	14	14	14	14	14	14
		T478K	E484A	F486V	Q498R	N501Y	Y505H	D614G	H655Y	N679K	P681H	N764K
		14	14	14	14	14	14	14	14	14	14	14
		D796Y	Q954H	N969K								
		14	14	14								
5	BF.14	T19I	L24-	P25-	P26-	A27S	H69-	V70-	G142D	V213G	G339D	S371F
		5	5	5	5	5	5	5	5	5	5	5
		S373P	S375F	T376A	D405N	R408S	K417N	N440K	N450D	L452R	S477N	T478K
		5	5	5	5	5	5	5	5	5	5	5
		E484A	F486V	Q498R	N501Y	Y505H	D614G	H655Y	N679K	P681H	N764K	D796Y
		5	5	5	5	5	5	5	5	5	5	5
		Q954H	N969K									
		5	5									
11	BF.40	T19I	L24-	P25-	P26-	A27S	H69-	V70-	G142D	V213G	G339D	S371F
		11	11	11	11	11	11	11	11	11	11	11
		S373P	S375F	T376A	D405N	R408S	K417N	N440K	V445A	L452R	S477N	T478K
		11	11	11	11	11	11	11	11	11	11	11
		E484A	F486V	Q498R	N501Y	Y505H	D614G	H655Y	N679K	P681H	N764K	D796Y
		11	11	11	11	11	11	11	11	11	11	11
		Q954H	N969K									
		11	11									
5	BA.5.2.6	T19I	L24-	P25-	P26-	A27S	H69-	V70-	G142D	V213G	G339D	R346T
		5	5	5	5	5	4	4	5	5	5	5
		S371F	S373P	S375F	T376A	D405N	R408S	K417N	N440K	L452R	S477N	T478K
		5	5	5	5	5	5	5	5	5	5	5
		E484A	F486V	Q498R	N501Y	Y505H	D614G	H655Y	N679K	P681H	N764K	D796Y
		5	5	5	5	5	5	5	5	5	5	5
		Q954H	N969K									
		5	5									
1	CR.1.3	T19I	L24-	P25-	P26-	A27S	H69-	V70-	G142D	Y144-	H146Q	V213G

		1	1	1	1	1	1	1	1	1	1	1
		G339D	R346T	S371F	S373P	S375F	T376A	D405N	R408S	K417N	N440K	K444R
		1	1	1	1	1	1	1	1	1	1	1
		L452R	S477N	T478K	E484A	F486V	Q498R	N501Y	Y505H	D614G	H655Y	N679K
		1	1	1	1	1	1	1	1	1	1	1
		P681H	N764K	D796Y	Q954H	N969K						
		1	1	1	1	1						
5	BA.5.2.20	T19I	L24-	P25-	P26-	A27S	H69-	V70-	G142D	V213G	G339D	S371F
		5	5	5	5	5	5	5	5	5	5	5
		S373P	S375F	T376A	D405N	R408S	K417N	N440K	L452R	S477N	T478K	E484A
		5	5	5	5	5	5	5	5	5	5	5
		F486V	Q498R	N501Y	Y505H	D614G	H655Y	N679K	P681H	N764K	D796Y	Q954H
		5	5	5	5	5	5	5	5	5	5	5
		N969K										
		5										
1	CK.2	T19I	L24-	P25-	P26-	A27S	H69-	V70-	G142D	V213G	G339D	S371F
		1	1	1	1	1	1	1	1	1	1	1
		S373P	S375F	T376A	D405N	R408S	K417N	N440K	K444N	L452R	S477N	T478K
		1	1	1	1	1	1	1	1	1	1	1
		E484A	F486V	Q498R	N501Y	Y505H	D614G	H655Y	N679K	P681H	N764K	D796Y
		1	1	1	1	1	1	1	1	1	1	1
		Q954H	N969K									
		1	1									
2	BA.5.2.26	T19I	L24-	P25-	P26-	A27S	H69-	V70-	G142D	V213G	G339D	S371F
		2	2	2	2	2	2	2	2	2	2	2
		S373P	S375F	T376A	D405N	R408S	K417N	N440K	L452R	S477N	T478K	E484A
		2	2	2	2	2	2	2	2	2	2	2
		F486V	Q498R	N501Y	Y505H	D614G	H655Y	N679K	P681H	N764K	D796Y	Q954H
		2	2	2	2	2	2	2	2	2	2	2
		N969K										
		2										

15	BA.5.2.34	T19I	L24-	P25-	P26-	A27S	H69-	V70-	G142D	V213G	G339D	R346T
		15	15	15	15	15	15	15	15	15	15	15
		S371F	S373P	S375F	T376A	D405N	R408S	K417N	N440K	L452R	S477N	T478K
		15	15	15	15	15	15	15	15	15	15	15
		E484A	F486V	Q498R	N501Y	Y505H	D614G	H655Y	N679K	P681H	N764K	D796Y
		15	15	15	15	15	15	15	15	15	15	15
		Q954H	N969K									
		15	15									
2	BA.5.2.36	T19I	L24-	P25-	P26-	A27S	H69-	V70-	G142D	V213G	G339D	S371F
		2	2	2	2	2	2	2	2	2	2	2
		S373P	S375F	T376A	D405N	R408S	K417N	N440K	K444T	L452R	S477N	T478K
		2	2	2	2	2	2	2	2	2	2	2
		E484A	F486V	Q498R	N501Y	Y505H	D614G	H655Y	N679K	P681H	N764K	D796Y
		2	2	2	2	2	2	2	2	2	2	2
		Q954H	N969K									
		2	2									
1	DQ.1	T19I	L24-	P25-	P26-	A27S	H69-	V70-	G142D	K147N	V213G	G339D
		1	1	1	1	1	1	1	1	1	1	1
		R346T	S371F	S373P	S375F	T376A	D405N	R408S	K417N	N440K	L452R	S477N
		1	1	1	1	1	1	1	1	1	1	1
		T478K	E484A	F486V	Q498R	N501Y	Y505H	D614G	H655Y	N679K	P681H	N764K
		1	1	1	1	1	1	1	1	1	1	1
		D796Y	Q954H	N969K								
		1	1	1								
1	BA.5.2.60	T19I	L24-	P25-	P26-	A27S	H69-	V70-	G142D	V213G	G339D	S371F
		1	1	1	1	1	1	1	1	1	1	1
		S373P	S375F	T376A	D405N	R408S	K417N	N440K	N450D	L452R	S477N	T478K
		1	1	1	1	1	1	1	1	1	1	1
		E484A	F486V	Q498R	N501Y	Y505H	D614G	H655Y	N679K	P681H	N764K	D796Y
		1	1	1	1	1	1	1	1	1	1	1
		Q954H	N969K									

		1	1									
1	BE.1.1	T19I	L24-	P25-	P26-	A27S	H69-	V70-	G142D	V213G	G339D	S371F
		1	1	1	1	1	1	1	1	1	1	1
		S373P	S375F	T376A	D405N	R408S	K417N	N440K	L452R	S477N	T478K	E484A
		1	1	1	1	1	1	1	1	1	1	1
		F486V	Q498R	N501Y	Y505H	D614G	H655Y	N679K	P681H	N764K	D796Y	Q954H
		1	1	1	1	1	1	1	1	1	1	1
		N969K										
		1										
3	BQ.1	T19I	L24-	P25-	P26-	A27S	H69-	V70-	G142D	V213G	G339D	S371F
		3	3	3	3	3	3	3	3	3	3	3
		S373P	S375F	T376A	D405N	R408S	K417N	N440K	K444T	L452R	N460K	S477N
		3	3	3	3	3	3	3	3	3	3	3
		T478K	E484A	F486V	Q498R	N501Y	Y505H	D614G	H655Y	N679K	P681H	N764K
		3	3	3	3	3	3	3	3	3	3	3
		D796Y	Q954H	N969K								
		3	3	3								
95	BQ.1.1	T19I	L24-	P25-	P26-	A27S	H69-	V70-	G142D	V213G	G339D	R346T
		95	95	95	95	95	92	91	95	95	94	95
		S371F	S373P	S375F	T376A	D405N	R408S	K417N	N440K	K444T	L452R	N460K
		95	95	95	95	95	95	95	95	95	95	95
		S477N	T478K	E484A	F486V	Q498R	N501Y	Y505H	D614G	H655Y	N679K	P681H
		95	95	95	95	95	95	95	95	95	95	95
		N764K	D796Y	Q954H	N969K							
		95	95	95	95							
3	BQ.1.1.1	T19I	L24-	P25-	P26-	A27S	H69-	V70-	G142D	V213G	G339D	R346T
		3	3	3	3	3	3	3	3	3	3	3
		S371F	S373P	S375F	T376A	D405N	R408S	K417N	N440K	K444T	L452R	N460K
		3	3	3	3	3	3	3	3	3	3	3
		S477N	T478K	E484A	F486V	Q498R	N501Y	Y505H	D614G	H655Y	N679K	P681H
		3	3	3	3	3	3	3	3	3	3	3

		N764K	D796Y	Q954H	N969K							
		3	3	3	3							
2	BQ.1.1.3	T19I	L24-	P25-	P26-	A27S	H69-	V70-	G142D	V213G	G339D	R346T
		2	2	2	2	2	2	2	2	2	2	2
		S371F	S373P	S375F	T376A	D405N	R408S	K417N	N440K	K444T	L452R	N460K
		2	2	2	2	2	2	2	2	2	2	2
		S477N	T478K	E484A	F486V	Q498R	N501Y	Y505H	D614G	H655Y	N679K	P681H
		2	2	2	2	2	2	2	2	2	2	2
		N764K	D796Y	Q954H	N969K							
		2	2	2	2							
1	BQ.1.1.4	T19I	L24-	P25-	P26-	A27S	H69-	V70-	G142D	V213G	G339D	R346T
		1	1	1	1	1	1	1	1	1	1	1
		S371F	S373P	S375F	T376A	D405N	R408S	K417N	N440K	K444T	L452R	N460K
		1	1	1	1	1	1	1	1	1	1	1
		S477N	T478K	E484A	F486V	Q498R	N501Y	Y505H	D614G	H655Y	N679K	P681H
		1	1	1	1	1	1	1	1	1	1	1
		N764K	D796Y	Q954H	N969K							
		1	1	1	1							
1	DN.1	T19I	L24-	P25-	P26-	A27S	H69-	V70-	G142D	K147N	V213G	G339D
		1	1	1	1	1	1	1	1	1	1	1
		R346T	S371F	S373P	S375F	T376A	D405N	R408S	K417N	N440K	K444T	L452R
		1	1	1	1	1	1	1	1	1	1	1
		N460K	S477N	T478K	E484A	F486V	Q498R	N501Y	Y505H	D614G	H655Y	N679K
		1	1	1	1	1	1	1	1	1	1	1
		P681H	N764K	D796Y	Q954H	N969K						
		1	1	1	1	1						
1	BQ.1.1.7	T19I	L24-	P25-	P26-	A27S	H69-	V70-	G142D	V213G	G339D	R346T
		1	1	1	1	1	1	1	1	1	1	1
		S371F	S373P	S375F	T376A	D405N	R408S	K417N	N440K	K444T	L452R	N460K
		1	1	1	1	1	1	1	1	1	1	1
		S477N	T478K	E484A	F486V	Q498R	N501Y	Y505H	D614G	H655Y	N679K	P681H

		1	1	1	1	1	1	1	1	1	1	1
		N764K	D796Y	Q954H	N969K							
		1	1	1	1							
2	EF.1.1	T19I	L24-	P25-	P26-	A27S	H69-	V70-	G142D	Y144-	V213G	G339D
		2	2	2	2	2	2	2	2	2	2	2
		R346T	S371F	S373P	S375F	T376A	D405N	R408S	K417N	N440K	K444T	L452R
		2	2	2	2	2	2	2	2	2	2	2
		N460K	S477N	T478K	E484A	F486V	Q498R	N501Y	Y505H	D614G	H655Y	N679K
		2	2	2	2	2	2	2	2	2	2	2
		P681H	N764K	D796Y	Q954H	N969K						
		2	2	2	2	2						
2	BQ.1.1.15	T19I	L24-	P25-	P26-	A27S	H69-	V70-	G142D	V213G	G339D	R346T
		2	2	2	2	2	2	2	2	2	2	2
		S371F	S373P	S375F	T376A	D405N	R408S	K417N	N440K	K444T	L452R	N460K
		2	2	2	2	2	2	2	2	2	2	2
		S477N	T478K	E484A	F486V	Q498R	N501Y	Y505H	D614G	H655Y	N679K	P681H
		2	2	2	2	2	2	2	2	2	2	2
		N764K	D796Y	Q954H	N969K							
		2	2	2	2							
1	BQ.1.1.20	T19I	L24-	P25-	P26-	A27S	H69-	V70-	G142D	Y144-	V213G	G339D
		1	1	1	1	1	1	1	1	1	1	1
		R346T	S371F	S373P	S375F	T376A	D405N	R408S	K417N	N440K	K444T	L452R
		1	1	1	1	1	1	1	1	1	1	1
		N460K	S477N	T478K	E484A	F486V	Q498R	N501Y	Y505H	D614G	H655Y	N679K
		1	1	1	1	1	1	1	1	1	1	1
		P681H	N764K	D796Y	Q954H	N969K						
		1	1	1	1	1						
1	BQ.1.1.22	T19I	L24-	P25-	P26-	A27S	H69-	V70-	G142D	Y144-	V213G	G339D
		1	1	1	1	1	1	1	1	1	1	1
		R346T	S371F	S373P	S375F	T376A	D405N	R408S	K417N	N440K	K444T	L452R
		1	1	1	1	1	1	1	1	1	1	1

		N460K	S477N	T478K	E484A	F486V	Q498R	N501Y	Y505H	D614G	H655Y	N679K
		1	1	1	1	1	1	1	1	1	1	1
		P681H	N764K	D796Y	Q954H	N969K						
		1	1	1	1	1						
1	ET.1	T19I	L24-	P25-	P26-	A27S	H69-	V70-	G142D	K147E	K182N	V213G
		1	1	1	1	1	1	1	1	1	1	1
		G339D	R346T	S371F	S373P	S375F	T376A	D405N	R408S	K417N	N440K	K444T
		1	1	1	1	1	1	1	1	1	1	1
		L452R	N460K	S477N	T478K	E484A	F486V	Q498R	N501Y	Y505H	D614G	H655Y
		1	1	1	1	1	1	1	1	1	1	1
		N679K	P681H	N764K	D796Y	Q954H	N969K					
		1	1	1	1	1	1					
1	BQ.1.1.44	T19I	L24-	P25-	P26-	A27S	H69-	V70-	G142D	V213G	D253G	G339D
		1	1	1	1	1	1	1	1	1	1	1
		R346T	S371F	S373P	S375F	T376A	D405N	R408S	K417N	N440K	K444T	L452R
		1	1	1	1	1	1	1	1	1	1	1
		N460K	S477N	T478K	E484A	F486V	Q498R	N501Y	Y505H	D614G	H655Y	N679K
		1	1	1	1	1	1	1	1	1	1	1
		P681H	N764K	D796Y	Q954H	N969K						
		1	1	1	1	1						
1	BQ.1.1.45	T19I	L24-	P25-	P26-	A27S	H69-	V70-	G142D	V213G	Y248D	G339D
		1	1	1	1	1	1	1	1	1	1	1
		R346T	S371F	S373P	S375F	T376A	D405N	R408S	K417N	N440K	K444T	L452R
		1	1	1	1	1	1	1	1	1	1	1
		N460K	S477N	T478K	E484A	F486V	Q498R	N501Y	Y505H	D614G	H655Y	N679K
		1	1	1	1	1	1	1	1	1	1	1
		P681H	N764K	D796Y	Q954H	N969K						
		1	1	1	1	1						
1	BQ.1.1.47	T19I	L24-	P25-	P26-	A27S	H69-	V70-	G142D	K147I	V213G	G339D
		1	1	1	1	1	1	1	1	1	1	1
		R346T	S371F	S373P	S375F	T376A	D405N	R408S	K417N	N440K	K444T	L452R

		1	1	1	1	1	1	1	1	1	1	1
		N460K	S477N	T478K	E484A	F486V	Q498R	N501Y	Y505H	D614G	H655Y	N679K
		1	1	1	1	1	1	1	1	1	1	1
		P681H	N764K	D796Y	Q954H	N969K						
		1	1	1	1	1						
1	BQ.1.1.55	T19I	L24-	P25-	P26-	A27S	H69-	V70-	G142D	Y144-	V213G	G339D
		1	1	1	1	1	1	1	1	1	1	1
		R346T	S371F	S373P	S375F	T376A	D405N	R408S	K417N	N440K	K444T	L452R
		1	1	1	1	1	1	1	1	1	1	1
		N460K	S477N	T478K	E484A	F486V	Q498R	N501Y	Y505H	D614G	H655Y	N679K
		1	1	1	1	1	1	1	1	1	1	1
		P681H	N764K	D796Y	Q954H	N969K	K1149R					
		1	1	1	1	1	1					
1	BQ.1.1.56	T19I	L24-	P25-	P26-	A27S	H69-	V70-	G142D	V213G	G339D	R346T
		1	1	1	1	1	1	1	1	1	1	1
		S371F	S373P	S375F	T376A	D405N	R408S	K417N	N440K	K444T	L452R	N460K
		1	1	1	1	1	1	1	1	1	1	1
		S477N	T478K	E484A	F486V	Q498R	N501Y	Y505H	D614G	H655Y	N679K	P681H
		1	1	1	1	1	1	1	1	1	1	1
		N764K	D796Y	Q954H	N969K	H1101Y						
		1	1	1	1	1						
1	BQ.1.1.66	L24-	P25-	P26-	A27S	H69-	V70-	G142D	K147N	V213G	G339D	R346T
		1	1	1	1	1	1	1	1	1	1	1
		S371F	S373P	S375F	T376A	D405N	R408S	K417N	N440K	K444T	L452R	N460K
		1	1	1	1	1	1	1	1	1	1	1
		S477N	T478K	E484A	F486V	Q498R	N501Y	Y505H	D614G	H655Y	N679K	P681H
		1	1	1	1	1	1	1	1	1	1	1
		N764K	D796Y	Q954H	N969K							
		1	1	1	1							
13	BQ.1.1.79	T19I	L24-	P25-	P26-	A27S	H69-	V70-	G142D	V213G	G339D	R346T
		13	13	13	13	13	13	13	13	13	13	13

		S371F	S373P	S375F	T376A	D405N	R408S	K417N	N440K	K444T	L452R	N460K
		13	13	13	13	13	13	13	13	13	13	13
		S477N	T478K	E484A	F486V	Q498R	N501Y	Y505H	Q613H	D614G	H655Y	N679K
		13	13	13	13	13	13	13	13	13	13	13
		P681H	N764K	D796Y	Q954H	N969K						
		13	13	13	13	13						
		T19I	L24-	P25-	P26-	A27S	H69-	V70-	G142D	V213G	G339D	S371F
		5	5	5	5	5	5	5	5	5	5	5
		S373P	S375F	T376A	D405N	R408S	K417N	N440K	K444T	L452R	N460K	S477N
5	BQ.1.2	5	5	5	5	5	5	5	5	5	5	5
		T478K	E484A	F486V	Q498R	N501Y	Y505H	D614G	H655Y	I666V	N679K	P681H
		5	5	5	5	5	5	5	5	5	5	5
		N764K	D796Y	Q954H	N969K							
		5	5	5	5							
		T19I	L24-	P25-	P26-	A27S	H69-	V70-	G142D	V213G	G339D	S371F
		5	5	5	5	5	5	5	5	5	4	4
		S373P	S375F	T376A	D405N	R408S	K417N	N440K	K444T	L452R	N460K	S477N
5	BQ.1.5	5	5	5	5	5	5	5	5	5	5	5
		T478K	E484A	F486V	Q498R	N501Y	Y505H	D614G	H655Y	N679K	P681H	N764K
		5	5	5	5	5	5	5	5	5	5	5
		D796Y	Q954H	N969K								
		5	5	5								
		T19I	L24-	P25-	P26-	A27S	H69-	V70-	G142D	Y144-	V213G	G339D
		1	1	1	1	1	1	1	1	1	1	1
		S371F	S373P	S375F	T376A	D405N	R408S	K417N	N440K	K444T	L452R	N460K
		1	1	1	1	1	1	1	1	1	1	1
1	BQ.1.8	S477N	T478K	E484A	F486V	Q498R	N501Y	Y505H	D614G	H655Y	N679K	P681H
		1	1	1	1	1	1	1	1	1	1	1
		N764K	D796Y	Q954H	N969K							
		1	1	1	1							
1	BQ.1.10	T19I	L24-	P25-	P26-	A27S	H69-	V70-	G142D	V213G	G339D	S371F

		1	1	1	1	1	1	1	1	1	1	1
		S373P	S375F	T376A	D405N	R408S	K417N	N440K	K444T	L452R	N460K	S477N
		1	1	1	1	1	1	1	1	1	1	1
		T478K	E484A	F486V	Q498R	N501Y	Y505H	D614G	H655Y	N679K	P681H	N764K
		1	1	1	1	1	1	1	1	1	1	1
		D796Y	Q954H	N969K								
		1	1	1								
11	BQ.1.10.2	T19I	L24-	P25-	P26-	A27S	H69-	V70-	G142D	V213G	G339D	S371F
		11	11	11	11	11	11	11	11	11	11	11
		S373P	S375F	T376A	D405N	R408S	K417N	N440K	K444T	L452R	N460K	S477N
		11	11	11	11	11	11	11	11	11	11	11
		T478K	E484A	F486V	Q498R	N501Y	Y505H	T523N	D614G	H655Y	N679K	P681H
		11	11	11	11	11	11	11	11	11	11	11
		N764K	D796Y	Q954H	N969K							
		11	11	11	11							
3	BQ.1.13.1	T19I	L24-	P25-	P26-	A27S	H69-	V70-	G142D	Y144-	F157L	V213G
		3	3	3	3	3	3	3	3	3	3	3
		G339D	S371F	S373P	S375F	T376A	D405N	R408S	K417N	N440K	K444T	L452R
		3	3	3	3	3	3	3	3	3	3	3
		N460K	S477N	T478K	E484A	F486V	Q498R	N501Y	Y505H	D614G	H655Y	N679K
		3	3	3	3	3	3	3	3	3	3	3
		P681H	N764K	D796Y	Q954H	N969K						
		3	3	3	3	3						
2	BQ.1.18	T19I	L24-	P25-	P26-	A27S	H69-	V70-	G142D	Y144-	V213G	G339D
		2	2	2	2	2	2	2	2	2	2	2
		R346T	S371F	S373P	S375F	T376A	D405N	R408S	K417N	N440K	K444T	L452R
		2	2	2	2	2	2	2	2	2	2	2
		N460K	S477N	T478K	E484A	F486V	Q498R	N501Y	Y505H	D614G	H655Y	N679K
		2	2	2	2	2	2	2	2	2	2	2
		P681H	N764K	D796Y	Q954H	N969K						
		2	2	2	2	2						

1	BA.5.9	T19I	L24-	P25-	P26-	A27S	H69-	V70-	G142D	V213G	G339D	R346I
		1	1	1	1	1	1	1	1	1	1	1
		S371F	S373P	S375F	T376A	D405N	R408S	K417N	N440K	L452R	S477N	T478K
		1	1	1	1	1	1	1	1	1	1	1
		E484A	F486V	Q498R	N501Y	Y505H	D614G	H655Y	N679K	P681H	N764K	D796Y
		1	1	1	1	1	1	1	1	1	1	1
		Q954H	N969K									
		1	1									
6	XBB.1	T19I	L24-	P25-	P26-	A27S	V83A	G142D	Y144-	H146Q	Q183E	V213E
		6	6	6	6	6	6	6	6	6	6	6
		G252V	G339H	R346T	L368I	S371F	S373P	S375F	T376A	D405N	R408S	K417N
		3	6	6	6	6	6	6	6	6	6	6
		N440K	V445P	G446S	N460K	S477N	T478K	E484A	F486S	F490S	Q498R	N501Y
		6	6	6	6	6	6	6	5	6	6	6
		Y505H	D614G	H655Y	N679K	P681H	N764K	D796Y	Q954H	N969K		
		5	6	6	6	6	6	6	6	6		
52	XBB.1.5	T19I	L24-	P25-	P26-	A27S	V83A	G142D	Y144-	H146Q	Q183E	V213E
		51	52	52	52	52	52	51	49	45	51	52
		G252V	G339H	R346T	L368I	S371F	S373P	S375F	T376A	D405N	R408S	K417N
		45	52	52	52	52	52	52	52	52	52	52
		N440K	V445P	G446S	N460K	S477N	T478K	E484A	F486P	F490S	Q498R	N501Y
		52	52	52	52	52	52	52	52	52	52	52
		Y505H	D614G	H655Y	N679K	P681H	N764K	D796Y	Q954H	N969K		
		52	52	52	52	52	52	52	52	52		
1	XBB.1.5.1	T19I	L24-	P25-	P26-	A27S	V83A	G142D	Y144-	H146Q	Q183E	V213E
		1	1	1	1	1	1	1	1	1	1	1
		G252V	G339H	R346T	L368I	S371F	S373P	S375F	T376A	D405N	R408S	K417N
		1	1	1	1	1	1	1	1	1	1	1
		N440K	V445P	G446S	N460K	S477N	T478K	E484A	F486P	F490S	Q498R	N501Y
		1	1	1	1	1	1	1	1	1	1	1
		Y505H	T573I	D614G	H655Y	N679K	P681H	N764K	D796Y	Q954H	N969K	

		1	1	1	1	1	1	1	1	1	1	
1	EM.1	T19I	L24-	P25-	P26-	A27S	V83A	G142D	Y144-	H146Q	Q183E	V213E
		1	1	1	1	1	1	1	1	1	1	1
		R214L	G252V	G339H	R346T	L368I	S371F	S373P	S375F	T376A	D405N	R408S
		1	1	1	1	1	1	1	1	1	1	1
		K417N	N440K	V445P	G446S	N460K	S477N	T478K	E484A	F486P	F490S	Q498R
		1	1	1	1	1	1	1	1	1	1	1
		N501Y	Y505H	D614G	H655Y	N679K	P681H	N764K	D796Y	Q954H	N969K	
		1	1	1	1	1	1	1	1	1	1	
1	XBB.1.5.11	T19I	L24-	P25-	P26-	A27S	V83A	G142D	Y144-	H146Q	Q183E	V213E
		1	1	1	1	1	1	1	1	1	1	1
		G252V	G339H	R346T	L368I	S371F	S373P	S375F	T376A	D405N	R408S	K417N
		1	1	1	1	1	1	1	1	1	1	1
		N440K	V445P	G446S	N460K	S477N	T478K	E484A	F486P	F490S	Q498R	N501Y
		1	1	1	1	1	1	1	1	1	1	1
		Y505H	D614G	H655Y	N679K	P681H	N764K	D796Y	Q954H	N969K		
		1	1	1	1	1	1	1	1	1		
4	XBB.1.5.12	T19I	L24-	P25-	P26-	A27S	V83A	G142D	Y144-	H146Q	Q183E	V213E
		4	4	4	4	4	4	4	4	4	4	4
		G252V	G339H	R346T	L368I	S371F	S373P	S375F	T376A	D405N	R408S	K417N
		4	4	4	4	4	4	4	4	4	4	4
		N440K	V445P	G446S	N460K	S477N	T478K	E484A	F486P	F490S	Q498R	N501Y
		4	4	4	4	4	4	4	4	4	4	4
		Y505H	D614G	H655Y	N679K	P681H	N764K	D796Y	Q954H	N969K		
		4	4	4	4	4	4	4	4	4		
1	XBB.1.5.13	T19I	L24-	P25-	P26-	A27S	V83A	G142D	Y144-	H146Q	Q183E	V213E
		1	1	1	1	1	1	1	1	1	1	1
		G252V	R346T	L368I	S371F	S373P	S375F	T376A	D405N	R408S	K417N	N440K
		1	1	1	1	1	1	1	1	1	1	1
		V445P	G446S	N460K	S477N	T478K	E484A	F486P	F490S	Q498R	N501Y	Y505H
		1	1	1	1	1	1	1	1	1	1	1

		D614G	H655Y	N679K	P681H	N764K	D796Y	Q954H	N969K			
		1	1	1	1	1	1	1	1			
1	XBB.1.5.14	T19I	L24-	P25-	P26-	A27S	V83A	G142D	Y144-	H146Q	Q183E	V213E
		1	1	1	1	1	1	1	1	1	1	1
		G252V	G339H	R346T	L368I	S371F	S373P	S375F	T376A	D405N	R408S	K417N
		1	1	1	1	1	1	1	1	1	1	1
		N440K	V445P	G446S	N460K	S477N	T478K	E484A	F486P	F490S	Q498R	N501Y
		1	1	1	1	1	1	1	1	1	1	1
		Y505H	D614G	H655Y	N679K	P681H	N764K	D796Y	Q954H	N969K		
		1	1	1	1	1	1	1	1	1		
1	XBB.1.5.15	T19I	L24-	P25-	P26-	A27S	V83A	G142D	Y144-	H146Q	Q183E	V213E
		1	1	1	1	1	1	1	1	1	1	1
		G252V	G339H	R346T	L368I	S371F	S373P	S375F	T376A	D405N	R408S	K417N
		1	1	1	1	1	1	1	1	1	1	1
		N440K	V445P	G446S	N460K	S477N	T478K	E484A	F486P	F490S	Q498R	N501Y
		1	1	1	1	1	1	1	1	1	1	1
		Y505H	D614G	H655Y	N679K	P681H	N764K	D796Y	Q954H	N969K		
		1	1	1	1	1	1	1	1	1		
1	FD.1	T19I	L24-	P25-	P26-	A27S	V83A	G142D	Y144-	H146Q	Q183E	V213E
		1	1	1	1	1	1	1	1	1	1	1
		G252V	G339H	R346T	L368I	S371F	S373P	S375F	T376A	D405N	R408S	K417N
		1	1	1	1	1	1	1	1	1	1	1
		N440K	V445P	G446S	N460K	S477N	T478K	E484A	F486P	F490S	Q498R	N501Y
		1	1	1	1	1	1	1	1	1	1	1
		Y505H	D614G	H655Y	N679K	P681H	N764K	D796Y	Q954H	N969K		
		1	1	1	1	1	1	1	1	1		
1	FD.3	T19I	L24-	P25-	P26-	A27S	V83A	G142D	Y144-	H146Q	Q183E	V213E
		1	1	1	1	1	1	1	1	1	1	1
		G252V	G339H	R346T	A348S	L368I	S371F	S373P	S375F	T376A	D405N	R408S
		1	1	1	1	1	1	1	1	1	1	1
		K417N	N440K	V445P	G446S	N460K	S477N	T478K	E484A	F486P	F490S	Q498R

		1	1	1	1	1	1	1	1	1	1	1
		N501Y	Y505H	D614G	H655Y	N679K	P681H	N764K	D796Y	Q954H	N969K	
		1	1	1	1	1	1	1	1	1	1	
4	XBB.1.5.18	T19I	L24-	P25-	P26-	A27S	V83A	G142D	Y144-	H146Q	Q183E	V213E
		4	4	4	4	4	4	4	4	4	4	4
		G252V	G339H	R346T	L368I	S371F	S373P	S375F	T376A	D405N	R408S	K417N
		4	4	4	4	4	4	4	4	4	4	4
		N440K	V445P	G446S	N460K	S477N	T478K	E484A	F486P	F490S	Q498R	N501Y
		4	4	4	4	4	4	4	4	4	4	4
		Y505H	D614G	H655Y	N679K	P681H	N764K	D796Y	Q954H	N969K		
		4	4	4	4	4	4	4	4	4		
2	XBB.1.5.24	T19I	L24-	P25-	P26-	A27S	V83A	G142D	Y144-	H146Q	Q183E	V213E
		2	2	2	2	2	2	2	2	2	2	2
		G252V	G339H	R346T	L368I	S371F	S373P	S375F	T376A	D405N	R408S	K417N
		2	2	2	2	2	2	2	2	2	2	2
		N440K	V445P	G446S	N460K	S477N	T478K	E484A	F486P	F490S	Q498R	N501Y
		2	2	2	2	2	2	2	2	2	2	2
		Y505H	D614G	H655Y	N679K	P681H	N764K	D796Y	Q954H	N969K		
		2	2	2	2	2	2	2	2	2		
11	EU.1.1	T19I	L24-	P25-	P26-	A27S	V83A	G142D	Y144-	H146Q	Q183E	V213E
		11	11	11	11	11	11	11	9	9	11	11
		G252V	G339H	R346T	L368I	S371F	S373P	S375F	T376A	D405N	R408S	I410V
		11	11	11	11	11	11	11	11	11	11	11
		K417N	N440K	V445P	G446S	N460K	S477N	T478K	E484A	F486P	F490S	Q498R
		11	11	11	11	11	11	11	11	11	11	11
		N501Y	Y505H	P521S	D614G	H655Y	N679K	P681H	N764K	D796Y	Q954H	N969K
		11	11	11	11	11	11	11	11	11	11	11
6	XBB.1.5.28	T19I	L24-	P25-	P26-	A27S	V83A	G142D	Y144-	H146Q	Q183E	V213E
		6	6	6	6	6	6	6	6	4	6	6
		G252V	G339H	R346T	L368I	S371F	S373P	S375F	T376A	D405N	R408S	K417N
		5	6	6	6	6	6	6	6	6	6	6

		N440K	V445P	G446S	N460K	S477N	T478R	E484A	F486P	F490S	Q498R	N501Y
		6	6	6	6	6	6	6	6	6	6	6
		Y505H	D614G	H655Y	N679K	P681H	N764K	D796Y	Q954H	N969K		
		6	6	6	6	6	6	6	6	6		
2	XBB.1.5.33	T19I	L24-	P25-	P26-	A27S	V83A	G142D	Y144-	H146Q	Q183E	V213E
		2	2	2	2	2	2	2	2	2	2	2
		G252V	G339H	R346T	L368I	S371F	S373P	S375F	T376A	D405N	R408S	K417N
		2	2	2	2	2	2	2	2	2	2	2
		N440K	V445P	G446S	N460K	S477N	T478K	E484A	F486P	F490S	Q498R	N501Y
		2	2	2	2	2	2	2	2	2	2	2
		Y505H	D614G	H655Y	N679K	P681H	N764K	D796Y	Q954H	N969K		
		2	2	2	2	2	2	2	2	2		
1	XBB.1.5.35	T19I	L24-	P25-	P26-	A27S	V83A	G142D	Y144-	H146Q	Q183E	V213E
		1	1	1	1	1	1	1	1	1	1	1
		G252V	G339H	R346T	L368I	S371F	S373P	S375F	T376A	D405N	R408S	K417N
		1	1	1	1	1	1	1	1	1	1	1
		N440K	V445P	G446S	N460K	S477N	T478K	E484A	F486P	F490S	Q498R	N501Y
		1	1	1	1	1	1	1	1	1	1	1
		Y505H	D614G	H655Y	N679K	P681H	N764K	D796Y	Q954H	N969K	N978S	
		1	1	1	1	1	1	1	1	1	1	
2	XBB.1.5.37	T19I	L24-	P25-	P26-	A27S	V83A	G142D	Y144-	H146Q	Q183E	V213E
		2	2	2	2	2	2	2	2	2	2	2
		G252V	G339H	R346T	L368I	S371F	S373P	S375F	T376A	D405N	R408S	K417N
		1	2	2	2	2	2	2	2	2	2	2
		N440K	V445P	G446S	N460K	S477N	T478K	E484A	F486P	F490S	Q498R	N501Y
		2	2	2	2	2	2	2	2	2	2	2
		Y505H	D614G	H655Y	N679K	P681H	N764K	D796Y	Q954H	N969K	K1045R	
		2	2	2	2	2	2	2	2	2	2	
1	XBB.1.5.38	T19I	L24-	P25-	P26-	A27S	V83A	G142D	Y144-	H146Q	Q183E	V213E
		1	1	1	1	1	1	1	1	1	1	1
		G252V	G339H	R346T	L368I	S371F	S373P	S375F	T376A	D405N	R408S	K417N

		1	1	1	1	1	1	1	1	1	1	1
		N440K	V445P	G446S	N460K	S477N	T478K	E484A	F486P	F490S	Q498R	N501Y
		1	1	1	1	1	1	1	1	1	1	1
		Y505H	D614G	H655Y	I666V	N679K	P681H	N764K	D796Y	Q954H	N969K	
		1	1	1	1	1	1	1	1	1	1	
1	XBB.1.5.46	T19I	L24-	P25-	P26-	A27S	V83A	G142D	Y144-	H146Q	Q183E	V213E
		1	1	1	1	1	1	1	1	1	1	1
		G252V	G339H	R346T	L368I	S371F	S373P	S375F	T376A	D405N	R408S	K417N
		1	1	1	1	1	1	1	1	1	1	1
		N440K	V445P	G446S	N460K	S477N	T478K	E484A	F486P	F490S	Q498R	N501Y
		1	1	1	1	1	1	1	1	1	1	1
		Y505H	D614G	H655Y	N679K	P681H	N764K	D796Y	Q954H	N969K		
		1	1	1	1	1	1	1	1	1		
4	GV.1	T19I	L24-	P25-	P26-	A27S	V83A	G142D	Y144-	H146Q	Q183E	V213E
		4	4	4	4	4	4	4	4	4	4	4
		G252V	G339H	R346T	L368I	S371F	S373P	S375F	T376A	D405N	R408S	K417N
		3	4	4	4	4	4	4	4	4	4	4
		N440K	V445P	G446S	N460K	S477N	E484A	F486P	F490S	Q498R	N501Y	Y505H
		4	4	4	4	4	4	4	4	4	4	4
		D614G	H655Y	N679K	P681H	N764K	D796Y	Q954H	N969K			
		4	4	4	4	4	4	4	4			
3	XBB.1.5.49	T19I	L24-	P25-	P26-	A27S	V83A	G142D	Y144-	H146Q	Q183E	V213E
		3	3	3	3	3	3	3	3	3	3	3
		G252V	G339H	R346T	L368I	S371F	S373P	S375F	T376A	D405N	R408S	K417N
		3	3	3	3	3	3	3	3	3	3	3
		N440K	V445P	G446S	N460K	S477N	T478K	E484A	F486P	F490S	Q498R	N501Y
		3	3	3	3	3	2	3	3	3	3	3
		Y505H	D614G	H655Y	N679K	P681H	N764K	D796Y	Q954H	N969K		
		3	3	3	3	3	3	3	3	3		
12	XBB.1.5.52	T19I	L24-	P25-	P26-	A27S	V83A	G142D	Y144-	H146Q	Q183E	V213E

		12	12	12	12	12	12	12	12	12	12	12
		G252V	G339H	R346T	L368I	S371F	S373P	S375F	T376A	D405N	R408S	K417N
		8	12	12	12	12	12	12	12	12	12	12
		N440K	V445P	G446S	N460K	S477N	T478K	E484A	F486P	F490S	Q498R	N501Y
		12	12	12	12	12	12	12	12	12	12	12
		Y505H	D614G	H655Y	N679K	P681H	N764K	D796Y	Q954H	N969K		
		12	12	12	12	12	12	12	12	12		
5	XBB.1.5.55	T19I	L24-	P25-	P26-	A27S	V83A	G142D	Y144-	H146Q	Q183E	V213E
		5	5	5	5	5	5	5	5	5	5	5
		G252V	G339H	R346T	L368I	S371F	S373P	S375F	T376A	D405N	R408S	K417N
		3	5	5	5	5	5	5	5	5	5	5
		N440K	V445P	G446S	N460K	S477N	T478K	E484A	F486P	F490S	Q498R	N501Y
		5	5	5	5	5	5	5	5	5	5	5
		Y505H	D614G	H655Y	N679K	P681H	N764K	D796Y	Q954H	N969K		
		5	5	5	5	5	5	5	5	5		
1	XBB.1.5.62	T19I	L24-	P25-	P26-	A27S	V83A	G142D	Y144-	H146Q	Q183E	V213E
		1	1	1	1	1	1	1	1	1	1	1
		G252V	G339H	R346T	L368I	S371F	S373P	S375F	T376A	D405N	R408S	K417N
		1	1	1	1	1	1	1	1	1	1	1
		N440K	V445P	G446S	N460K	S477N	T478K	E484A	F486P	F490S	Q498R	N501Y
		1	1	1	1	1	1	1	1	1	1	1
		Y505H	D614G	H655Y	N679K	P681H	N764K	D796Y	Q954H	N969K		
		1	1	1	1	1	1	1	1	1		
2	XBB.1.5.63	T19I	L24-	P25-	P26-	A27S	V83A	G142D	Y144-	H146Q	Q183E	V213E
		2	2	2	2	2	2	2	2	2	2	2
		G252V	G339H	R346T	L368I	S371F	S373P	S375F	T376A	D405N	R408S	K417N
		2	2	2	2	2	2	2	2	2	2	2
		N440K	V445P	G446S	N460K	S477N	T478K	E484A	F486P	F490S	Q498R	N501Y
		2	2	2	2	2	2	2	2	2	2	2
		Y505H	D614G	H655Y	N679K	P681H	N764K	D796Y	Q954H	N969K		
		2	2	2	2	2	2	2	2	2		

		T19I	L24-	P25-	P26-	A27S	V83A	G142D	Y144-	H146Q	Q183E	V213E
		1	1	1	1	1	1	1	1	1	1	1
		G252V	G339H	R346T	L368I	S371F	S373P	S375F	T376A	D405N	R408S	K417N
1	XBB.1.5.69	1	1	1	1	1	1	1	1	1	1	1
		N440K	V445P	G446S	N460K	S477N	T478K	E484A	F486P	F490S	Q498R	N501Y
		1	1	1	1	1	1	1	1	1	1	1
		Y505H	D614G	H655Y	N679K	P681H	N764K	D796Y	Q954H	N969K		
		1	1	1	1	1	1	1	1	1		
		T19I	L24-	P25-	P26-	A27S	V83A	G142D	Y144-	H146Q	Q183E	V213E
		3	3	3	3	3	2	3	3	3	3	3
		G252V	G339H	R346T	L368I	S371F	S373P	S375F	T376A	D405N	R408S	K417N
		2	3	3	3	3	3	3	3	3	3	3
3	GK.1.1	N440K	V445P	G446S	L455F	F456L	N460K	S477N	T478K	E484A	F486P	F490S
		3	3	3	3	3	3	3	3	3	3	3
		Q498R	N501Y	Y505H	T573I	D614G	H655Y	N679K	P681H	S704L	N764K	D796Y
		3	3	3	3	2	1	3	3	3	3	3
		Q954H	N969K									
		3	3									
		T19I	L24-	P25-	P26-	A27S	V83A	G142D	Y144-	H146Q	Q183E	V213E
		1	1	1	1	1	1	1	1	1	1	1
		G252V	G339H	R346T	L368I	S371F	S373P	S375F	T376A	D405N	R408S	K417N
		1	1	1	1	1	1	1	1	1	1	1
1	GK.1.1.1	N440K	V445P	G446S	L455F	F456L	N460K	S477N	T478K	E484A	F486P	F490S
		1	1	1	1	1	1	1	1	1	1	1
		Q498R	N501Y	Y505H	T573I	D614G	H655Y	N679K	P681H	S704L	N764K	D796Y
		1	1	1	1	1	1	1	1	1	1	1
		Q954H	N969K									
		1	1									
		T19I	L24-	P25-	P26-	A27S	V83A	G142D	Y144-	H146Q	Q183E	N185I
2	GK.8.1	2	2	2	2	2	2	2	2	2	2	2
		V213E	G252V	G339H	R346T	L368I	S371F	S373P	S375F	T376A	D405N	R408S

		2	2	2	2	2	2	2	2	2	2	2
		K417N	N440K	V445P	G446S	L455F	F456L	N460K	A475V	S477N	T478K	E484A
		2	2	2	2	2	2	2	2	2	2	2
		F486P	F490S	Q498R	N501Y	Y505H	D614G	H655Y	N679K	P681H	N764K	D796Y
		2	2	2	2	2	2	2	2	2	2	2
		Q954H	N969K									
		2	2									
4	XBB.1.5.90	T19I	L24-	P25-	P26-	A27S	V83A	G142D	Y144-	H146Q	Q183E	V213E
		4	4	4	4	4	4	4	4	4	4	4
		G252V	G339H	R346T	L368I	S371F	S373P	S375F	T376A	D405N	R408S	K417N
		4	4	4	4	4	4	4	4	4	4	4
		N440K	V445P	G446S	N460K	S477N	T478K	E484A	F486P	F490S	Q498R	N501Y
		4	4	4	4	4	4	4	4	4	4	4
		Y505H	D614G	P621S	H655Y	N679K	P681H	N764K	D796Y	Q954H	N969K	
		4	4	4	4	4	4	4	4	4	4	
6	JD.1.1	T19I	L24-	P25-	P26-	A27S	V83A	G142D	Y144-	H146Q	Q183E	V213E
		6	6	6	6	6	6	6	6	6	6	6
		G252V	G339H	R346T	L368I	S371F	S373P	S375F	T376A	D405N	R408S	K417N
		6	6	6	6	6	6	6	6	6	6	6
		N440K	V445P	G446S	L455F	F456L	N460K	A475V	S477N	T478K	E484A	F486P
		6	6	6	6	6	6	6	6	6	6	6
		F490S	Q498R	N501Y	Y505H	D614G	H655Y	N679K	P681H	N764K	D796Y	Q954H
		6	6	6	6	6	6	6	6	6	6	6
		N969K										
		6										
1	JD.1.1.1	T19I	L24-	P25-	P26-	A27S	V83A	G142D	Y144-	H146Q	Q183E	V213E
		1	1	1	1	1	1	1	1	1	1	1
		Y248H	G252V	G339H	R346T	L368I	S371F	S373P	S375F	T376A	D405N	R408S
		1	1	1	1	1	1	1	1	1	1	1
		K417N	N440K	V445P	G446S	L455F	F456L	N460K	A475V	S477N	T478K	E484A
		1	1	1	1	1	1	1	1	1	1	1

		F486P	F490S	Q498R	N501Y	Y505H	D614G	H655Y	N679K	P681H	N764K	D796Y
		1	1	1	1	1	1	1	1	1	1	1
		Q954H	N969K									
		1	1									
1	JD.1.1.5	T19I	L24-	P25-	P26-	A27S	V83A	G142D	Y144-	H146Q	Q183E	V213E
		1	1	1	1	1	1	1	1	1	1	1
		G252V	G339H	R346T	L368I	S371F	S373P	S375F	T376A	D405N	R408S	K417N
		1	1	1	1	1	1	1	1	1	1	1
		N440K	V445P	G446S	L455F	F456L	N460K	A475V	S477N	T478K	E484A	F486P
		1	1	1	1	1	1	1	1	1	1	1
		F490S	Q498R	N501Y	Y505H	D614G	H655Y	Q677R	N679K	P681H	N764K	D796Y
		1	1	1	1	1	1	1	1	1	1	1
		Q954H	N969K									
		1	1									
1	JD.1.2	T19I	L24-	P25-	P26-	A27S	V83A	G142D	Y144-	H146Q	Q183E	V213E
		1	1	1	1	1	1	1	1	1	1	1
		G252V	G339H	R346T	L368I	S371F	S373P	S375F	T376A	D405N	R408S	K417N
		1	1	1	1	1	1	1	1	1	1	1
		N440K	V445P	G446S	L455F	F456L	N460K	S477N	T478K	E484A	F486P	F490S
		1	1	1	1	1	1	1	1	1	1	1
		Q498R	N501Y	Y505H	D614G	H655Y	N679K	P681H	N764K	D796Y	Q954H	N969K
		1	1	1	1	1	1	1	1	1	1	1
		P1162S										
		1										
1	JD.2	T19I	L24-	P25-	P26-	A27S	V83A	G142D	Y144-	H146Q	Q183E	V213E
		1	1	1	1	1	1	1	1	1	1	1
		G339H	R346T	L368I	S371F	S373P	S375F	T376A	D405N	R408S	K417N	N440K
		1	1	1	1	1	1	1	1	1	1	1
		V445P	G446S	N460K	S477N	T478K	E484A	F486P	F490S	Q498R	N501Y	Y505H
		1	1	1	1	1	1	1	1	1	1	1
		D614G	H655Y	N679K	P681H	N764K	D796Y	Q954H	N969K			

		1	1	1	1	1	1	1	1			
63	XBB.1.9.1	T19I	L24-	P25-	P26-	A27S	V83A	G142D	Y144-	H146Q	Q183E	V213E
		63	63	63	63	63	63	63	63	63	63	63
		G252V	G339H	R346T	L368I	S371F	S373P	S375F	T376A	D405N	R408S	K417N
		48	63	63	63	63	63	63	63	63	63	63
		N440K	V445P	G446S	N460K	S477N	T478K	E484A	F486P	F490S	Q498R	N501Y
		63	63	63	63	63	58	63	63	63	63	63
		Y505H	D614G	H655Y	N679K	P681H	N764K	D796Y	Q954H	N969K		
		63	63	63	63	63	63	63	63	63		
17	FL.1.5.1	T19I	L24-	P25-	P26-	A27S	V83A	G142D	Y144-	H146Q	Q183E	V213E
		17	17	17	17	17	17	17	17	17	17	17
		G252V	G339H	R346T	L368I	S371F	S373P	S375F	T376A	D405N	R408S	K417N
		17	17	17	17	17	17	17	17	17	17	17
		N440K	V445P	G446S	F456L	N460K	S477N	T478R	E484A	F486P	F490S	Q498R
		17	17	17	17	17	17	17	17	17	17	17
		N501Y	Y505H	D614G	H655Y	N679K	P681H	A701V	N764K	D796Y	Q954H	N969K
		17	17	17	17	17	17	17	17	17	17	17
1	HN.4	T19I	L24-	P25-	P26-	A27S	V83A	G142D	Y144-	H146Q	Q183E	V213E
		1	1	1	1	1	1	1	1	1	1	1
		G252V	G339H	R346T	L368I	S371F	S373P	S375F	T376A	D405N	R408S	K417N
		1	1	1	1	1	1	1	1	1	1	1
		N440K	V445P	G446S	F456L	N460K	S477N	T478R	E484A	F486P	F490S	Q498R
		1	1	1	1	1	1	1	1	1	1	1
		N501Y	Y505H	D614G	H655Y	N679K	P681H	A701V	N764K	D796Y	Q954H	N969K
		1	1	1	1	1	1	1	1	1	1	1
1	HN.4.2	Q14H	T19I	L24-	P25-	P26-	A27S	V83A	G142D	Y144-	H146Q	Q183E
		1	1	1	1	1	1	1	1	1	1	1
		V213E	G252V	G339H	R346T	L368I	S371F	S373P	S375F	T376A	D405N	R408S
		1	1	1	1	1	1	1	1	1	1	1
		K417N	N440K	V445P	G446S	F456L	N460K	S477N	T478R	E484A	F486P	F490S
		1	1	1	1	1	1	1	1	1	1	1

		Q498R	N501Y	Y505H	D614G	H655Y	N679K	P681H	A701V	N764K	D796Y	Q954H
		1	1	1	1	1	1	1	1	1	1	1
		N969K										
		1										
6	HN.5	T19I	L24-	P25-	P26-	A27S	V83A	G142D	Y144-	H146Q	Q183E	V213E
		6	6	6	6	6	6	6	6	6	6	6
		G252V	G339H	R346T	L368I	S371F	S373P	S375F	T376A	D405N	R408S	K417N
		6	6	5	5	5	5	5	5	6	6	6
		N440K	V445P	G446S	F456L	N460K	S477N	T478R	E484A	F486P	F490S	Q498R
		6	6	6	6	6	6	6	6	6	6	6
		N501Y	Y505H	E554K	D614G	H655Y	N679K	P681H	A701V	N764K	D796Y	Q954H
		6	6	6	6	5	6	6	6	6	6	6
		N969K										
		6										
4	KC.1	T19I	L24-	P25-	P26-	A27S	V83A	G142D	Y144-	H146Q	Q183E	V213E
		4	4	4	4	4	4	4	4	4	4	4
		G252V	G339H	R346T	L368I	S371F	S373P	S375F	T376A	D405N	R408S	K417N
		4	4	4	4	4	4	4	4	4	4	4
		N440K	V445P	G446S	F456L	N460K	S477N	T478K	N481K	E484A	F486P	F490P
		4	4	4	4	4	4	4	4	4	4	4
		Q498R	N501Y	Y505H	D614G	H655Y	N679K	P681R	A701V	N764K	D796Y	Q954H
		4	4	4	4	4	4	4	4	4	4	4
		N969K										
		4										
1	FL.2	T19I	L24-	P25-	P26-	A27S	V83A	G142D	Y144-	H146Q	Q183E	V213E
		1	1	1	1	1	1	1	1	1	1	1
		G252V	G339H	R346T	L368I	S371F	S373P	S375F	T376A	D405N	R408S	K417N
		1	1	1	1	1	1	1	1	1	1	1
		N440K	V445P	G446S	N460K	S477N	E484A	F486P	F490S	Q498R	N501Y	Y505H
		1	1	1	1	1	1	1	1	1	1	1
		D614G	H655Y	N679K	P681H	N764K	D796Y	Q954H	N969K			

		1	1	1	1	1	1	1	1			
2	FL.3	T19I	L24-	P25-	P26-	A27S	V83A	G142D	Y144-	H146Q	Q183E	V213E
		2	2	2	2	2	2	2	2	2	2	2
		G252V	G339H	R346T	L368I	S371F	S373P	S375F	T376A	D405N	R408S	K417N
		2	2	2	2	2	2	2	2	2	2	2
		N440K	V445P	G446S	N460K	S477N	T478K	E484A	F486P	F490S	Q498R	N501Y
		2	2	2	2	2	2	2	2	2	2	2
		Y505H	D614G	H655Y	N679K	P681H	N764K	D796Y	Q954H	N969K		
		2	2	2	2	2	2	2	2	2		
19	FL.3.1	T19I	L24-	P25-	P26-	A27S	V83A	G142D	Y144-	H146Q	Q183E	V213E
		19	19	19	19	19	18	19	19	18	19	19
		G252V	G339H	R346T	L368I	S371F	S373P	S375F	T376A	D405N	R408S	K417N
		11	19	19	19	19	19	19	19	19	19	19
		N440K	V445P	G446S	N460K	S477N	T478K	E484A	F486P	F490S	Q498R	N501Y
		19	19	19	19	19	19	19	19	19	19	19
		Y505H	D614G	H655Y	N679K	P681H	N764K	D796Y	Q954H	N969K		
		19	19	19	19	19	19	19	19	19		
2	FL.5	T19I	L24-	P25-	P26-	A27S	V83A	G142D	Y144-	H146Q	Q183E	V213E
		2	2	2	2	2	2	2	2	2	2	2
		G252V	G339H	R346T	L368I	S371F	S373P	S375F	T376A	D405N	R408S	K417N
		1	2	2	2	2	2	2	2	2	2	2
		N440K	V445P	G446S	N460K	S477N	T478K	E484A	F486P	F490S	Q498R	N501Y
		2	2	2	2	2	2	2	2	2	2	2
		Y505H	D614G	H655Y	N679K	P681H	N764K	D796Y	Q954H	N969K		
		2	2	2	2	2	2	2	2	2		
3	FL.10	T19I	L24-	P25-	P26-	A27S	V83A	G142D	Y144-	H146Q	Q183E	V213E
		3	3	3	3	3	3	3	3	3	3	3
		G252V	G339H	R346T	L368I	S371F	S373P	S375F	T376A	D405N	R408S	K417N
		2	3	3	3	3	3	3	3	3	3	3
		N440K	V445P	G446S	N460K	S477N	T478K	E484A	F486P	F490S	Q498R	N501Y
		3	3	3	3	3	3	3	3	3	3	3

		Y505H	D614G	H655Y	N679K	P681H	N764K	D796Y	Q954H	N969K		
		3	3	3	3	3	3	3	3	3		
2	FL.13	T19I	L24-	P25-	P26-	A27S	V83A	G142D	Y144-	H146Q	Q183E	V213E
		2	2	2	2	2	2	2	2	1	2	2
		G252V	G339H	R346T	L368I	S371F	S373P	S375F	T376A	D405N	R408S	K417N
		2	2	2	2	2	2	2	2	2	2	2
		N440K	V445P	G446S	N460K	S477N	T478K	E484A	F486P	F490S	Q498R	N501Y
		2	2	2	2	2	2	2	2	2	2	2
		Y505H	D614G	H655Y	N679K	P681H	N764K	D796Y	Q954H	N969K		
		2	2	2	2	2	2	2	2	2		
6	FL.15	T19I	L24-	P25-	P26-	A27S	V83A	G142D	Y144-	H146Q	Q183E	V213E
		6	6	6	6	6	6	6	6	5	6	6
		G252V	G339H	R346T	L368I	S371F	S373P	S375F	T376A	D405N	R408S	K417N
		6	6	6	6	6	6	6	6	6	6	6
		N440K	V445P	G446S	F456L	N460K	S477N	T478K	E484A	F486P	F490S	Q498R
		6	6	6	6	6	6	6	6	6	6	6
		N501Y	Y505H	D614G	H655Y	N679K	P681H	N764K	D796Y	Q954H	N969K	
		6	6	6	5	6	6	6	5	6	6	
4	FL.15.1.1	T19I	L24-	P25-	P26-	A27S	V83A	G142D	Y144-	H146Q	Q183E	V213E
		4	4	4	4	4	4	4	4	4	4	4
		G252V	G339H	R346T	L368I	S371F	S373P	S375F	T376A	D405N	R408S	K417N
		4	4	4	4	4	4	4	4	4	4	4
		N440K	V445P	G446S	L455F	F456L	N460K	A475V	S477N	T478K	E484A	F486P
		4	4	4	4	4	4	4	4	4	4	4
		F490S	Q498R	N501Y	Y505H	D614G	H655Y	N679K	P681H	N764K	D796Y	Q954H
		4	4	4	4	4	4	4	4	4	4	4
		N969K										
		4										
1	FL.17	T19I	L24-	P25-	P26-	A27S	V83A	G142D	Y144-	H146Q	Q183E	G339H
		1	1	1	1	1	1	1	1	1	1	1
		R346T	L368I	S371F	S373P	S375F	T376A	D405N	R408S	K417N	N440K	V445P

		1	1	1	1	1	1	1	1	1	1	1
		G446S	N460K	S477N	T478K	E484A	F486P	F490S	Q498R	N501Y	Y505H	D614G
		1	1	1	1	1	1	1	1	1	1	1
		H655Y	N679K	P681H	N764K	D796Y	Q954H	N969K				
		1	1	1	1	1	1	1				
3	FL.18	T19I	L24-	P25-	P26-	A27S	V83A	G142D	Y144-	H146Q	Q183E	V213E
		3	3	3	3	3	3	3	3	3	3	3
		G252V	G339H	R346T	L368I	S371F	S373P	S375F	T376A	D405N	R408S	K417N
		1	3	3	3	3	3	3	3	3	3	3
		N440K	V445P	G446S	N460K	S477N	T478K	E484A	F486P	F490S	Q498R	N501Y
		3	3	3	3	3	3	3	3	3	3	3
		Y505H	D614G	H655Y	N679K	P681H	N764K	D796Y	Q954H	N969K		
		3	3	3	3	3	3	3	3	3		
1	FL.20	T19I	L24-	P25-	P26-	A27S	Q52H	V83A	G142D	Y144-	H146Q	Q183E
		1	1	1	1	1	1	1	1	1	1	1
		V213E	G252V	G339H	R346T	L368I	S371F	S373P	S375F	T376A	D405N	R408S
		1	1	1	1	1	1	1	1	1	1	1
		K417N	N440K	V445P	G446S	F456L	N460K	S477N	T478K	E484A	F486P	F490S
		1	1	1	1	1	1	1	1	1	1	1
		Q498R	N501Y	Y505H	D614G	H655Y	N679K	P681H	N764K	D796Y	Q954H	N969K
		1	1	1	1	1	1	1	1	1	1	1
1	FL.25	T19I	L24-	P25-	P26-	A27S	V83A	G142D	Y144-	Q183E	V213E	G252V
		1	1	1	1	1	1	1	1	1	1	1
		G339H	R346T	L368I	S371F	S373P	S375F	T376A	D405N	R408S	K417N	N440K
		1	1	1	1	1	1	1	1	1	1	1
		V445P	G446S	N460K	S477N	E484A	F486P	F490S	Q498R	N501Y	Y505H	E554K
		1	1	1	1	1	1	1	1	1	1	1
		D614G	H655Y	N679K	P681H	N764K	D796Y	Q954H	N969K			
		1	1	1	1	1	1	1	1			
1	FL.26	T19I	L24-	P25-	P26-	A27S	V83A	G142D	Y144-	H146Q	Q183E	V213E
		1	1	1	1	1	1	1	1	1	1	1

		G252V	G339H	R346T	L368I	S371F	S373P	S375F	T376A	D405N	R408S	K417N
		1	1	1	1	1	1	1	1	1	1	1
		N440K	V445P	G446S	N460K	S477N	T478K	E484A	F486P	F490S	Q498R	N501Y
		1	1	1	1	1	1	1	1	1	1	1
		Y505H	D614G	H655Y	N679K	P681H	N764K	D796Y	Q954H	N969K		
		1	1	1	1	1	1	1	1	1		
1	FL.40	T19I	L24-	P25-	P26-	A27S	V83A	G142D	Y144-	H146Q	Q183E	V213E
		1	1	1	1	1	1	1	1	1	1	1
		G339H	R346T	L368I	S371F	S373P	S375F	T376A	D405N	R408S	K417N	N440K
		1	1	1	1	1	1	1	1	1	1	1
		V445P	G446S	N460K	S477N	E484A	F486P	F490S	Q498R	N501Y	Y505H	D614G
		1	1	1	1	1	1	1	1	1	1	1
		H655Y	N679K	P681H	N764K	D796Y	Q954H	N969K				
		1	1	1	1	1	1	1				
1	XBB.1.9.2	T19I	L24-	P25-	P26-	A27S	V83A	G142D	Y144-	H146Q	Q183E	V213E
		1	1	1	1	1	1	1	1	1	1	1
		G339H	R346T	L368I	S371F	S373P	S375F	T376A	D405N	R408S	K417N	N440K
		1	1	1	1	1	1	1	1	1	1	1
		V445P	G446S	N460K	S477N	T478K	E484A	F486P	F490S	Q498R	N501Y	Y505H
		1	1	1	1	1	1	1	1	1	1	1
		D614G	H655Y	N679K	P681H	N764K	D796Y	Q954H	N969K			
		1	1	1	1	1	1	1	1			
26	EG.1	T19I	L24-	P25-	P26-	A27S	V83A	G142D	Y144-	H146Q	Q183E	V213E
		26	26	26	26	26	26	26	26	26	26	26
		G252V	G339H	R346T	L368I	S371F	S373P	S375F	T376A	D405N	R408S	K417N
		20	26	26	26	26	26	26	26	26	26	26
		N440K	V445P	G446S	N460K	S477N	T478K	E484A	F486P	F490S	Q498R	N501Y
		26	26	26	26	26	26	26	26	26	26	26
		Y505H	Q613H	D614G	H655Y	N679K	P681H	N764K	D796Y	Q954H	N969K	
		26	26	26	26	26	26	26	26	26	26	
2	EG.1.2	T19I	L24-	P25-	P26-	A27S	V83A	G142D	Y144-	H146Q	Q183E	V213E

		2	2	2	2	2	2	2	2	2	2	2
		G252V	G339H	R346T	L368I	S371F	S373P	S375F	T376A	D405N	R408S	K417N
		2	2	2	2	2	2	2	2	2	2	2
		N440K	V445P	G446S	N460K	S477N	T478K	E484A	F486P	F490S	Q498R	N501Y
		2	2	2	2	2	2	2	2	2	2	2
		Y505H	Q613H	D614G	H655Y	N679K	P681H	N764K	D796Y	Q954H	N969K	
		2	2	2	2	2	2	2	2	2	2	
		T19I	L24-	P25-	P26-	A27S	V83A	G142D	Y144-	H146Q	Q183E	V213E
		1	1	1	1	1	1	1	1	1	1	1
		G252V	G339H	R346T	L368I	S371F	S373P	S375F	T376A	D405N	R408S	K417N
		1	1	1	1	1	1	1	1	1	1	1
1	EG.1.3	N440K	V445P	G446S	N460K	S477N	T478K	E484A	F486P	F490S	Q498R	N501Y
		1	1	1	1	1	1	1	1	1	1	1
		Y505H	Q613H	D614G	H655Y	N679K	P681H	N764K	D796Y	Q954H	N969K	
		1	1	1	1	1	1	1	1	1	1	
		T19I	L24-	P25-	P26-	A27S	V83A	G142D	Q183E	V213E	G252V	G339H
		3	3	3	3	3	3	3	3	3	2	3
		R346T	L368I	S371F	S373P	S375F	T376A	D405N	R408S	K417N	N440K	V445P
		3	3	3	3	3	3	3	3	3	3	3
3	EG.1.6	G446S	N460K	S477N	T478K	E484A	F486P	F490S	Q498R	N501Y	Y505H	Q613H
		3	3	3	3	3	3	3	3	3	3	3
		D614G	H655Y	N679K	P681H	N764K	D796Y	Q954H	N969K			
		3	3	3	3	3	3	3	3			
		T19I	L24-	P25-	P26-	A27S	V83A	G142D	Y144-	H146Q	Q183E	V213E
		1	1	1	1	1	1	1	1	1	1	1
		G339H	R346T	L368I	S371F	S373P	S375F	T376A	D405N	R408S	K417N	N440K
		1	1	1	1	1	1	1	1	1	1	1
1	EG.5	V445P	G446S	F456L	N460K	S477N	T478K	E484A	F486P	F490S	Q498R	N501Y
		1	1	1	1	1	1	1	1	1	1	1
		Y505H	D614G	H655Y	N679K	P681H	N764K	D796Y	Q954H	N969K		
		1	1	1	1	1	1	1	1	1		

3	EG.5.1	T19I	L24-	P25-	P26-	A27S	Q52H	V83A	G142D	Y144-	H146Q	Q183E
		3	3	3	3	3	3	2	3	3	2	3
		V213E	G252V	G339H	R346T	L368I	S371F	S373P	S375F	T376A	D405N	R408S
		3	2	3	3	3	3	3	3	3	3	3
		K417N	N440K	V445P	G446S	F456L	N460K	S477N	T478K	E484A	F486P	F490S
		3	3	3	3	3	3	3	3	3	3	3
		Q498R	N501Y	Y505H	D614G	H655Y	N679K	P681H	N764K	D796Y	Q954H	N969K
		3	3	3	3	3	3	3	3	3	3	3
17	EG.5.1.1	T19I	L24-	P25-	P26-	A27S	Q52H	V83A	G142D	Y144-	H146Q	Q183E
		17	17	17	17	17	17	17	17	17	17	17
		V213E	G252V	G339H	R346T	L368I	S371F	S373P	S375F	T376A	D405N	R408S
		17	14	17	17	17	17	17	17	17	17	17
		K417N	N440K	V445P	G446S	F456L	N460K	S477N	T478K	E484A	F486P	F490S
		17	17	17	17	17	17	17	17	17	17	17
		Q498R	N501Y	Y505H	D614G	H655Y	N679K	P681H	N764K	D796Y	Q954H	N969K
		17	17	17	17	17	17	17	17	17	17	17
2	HK.1.2	T19I	L24-	P25-	P26-	A27S	Q52H	V83A	G142D	Y144-	H146Q	Q183E
		2	2	2	2	2	2	2	2	2	2	2
		V213E	G252V	G257V	T307I	G339H	R346T	L368I	S371F	S373P	S375F	T376A
		2	2	2	2	2	2	2	2	2	2	2
		D405N	R408S	K417N	N440K	V445P	G446S	L455F	F456L	N460K	S477N	T478K
		2	2	2	2	2	2	2	2	2	2	2
		E484A	F486P	F490S	Q498R	N501Y	Y505H	D614G	H655Y	N679K	P681H	N764K
		2	2	2	2	2	2	2	2	2	2	2
		D796Y	Q954H	N969K								
		2	2	2								
1	HK.2	Q14H	T19I	L24-	P25-	P26-	A27S	Q52H	V83A	G142D	Y144-	H146Q
		1	1	1	1	1	1	1	1	1	1	1
		Q183E	V213E	G252V	G339H	R346T	L368I	S371F	S373P	S375F	T376A	D405N
		1	1	1	1	1	1	1	1	1	1	1
		R408S	K417N	N440K	V445P	G446S	F456L	N460K	S477N	T478K	E484A	F486P

		1	1	1	1	1	1	1	1	1	1	1
		F490S	Q498R	N501Y	Y505H	D614G	H655Y	N679K	P681H	N764K	D796Y	Q954H
		1	1	1	1	1	1	1	1	1	1	1
		N969K										
		1										
24	HK.3	T19I	L24-	P25-	P26-	A27S	Q52H	V83A	G142D	Y144-	H146Q	Q183E
		24	24	24	24	24	24	24	24	24	24	24
		V213E	G252V	G339H	R346T	L368I	S371F	S373P	S375F	T376A	D405N	R408S
		24	24	24	24	24	24	24	24	24	24	24
		K417N	N440K	V445P	G446S	L455F	F456L	N460K	S477N	T478K	E484A	F486P
		24	24	24	24	24	24	24	24	24	24	23
		F490S	Q498R	N501Y	Y505H	D614G	H655Y	N679K	P681H	N764K	D796Y	Q954H
		24	24	24	24	24	24	24	24	24	24	24
		N969K										
		24										
2	HK.3.1	T19I	L24-	P25-	P26-	A27S	Q52H	V83A	G142D	Y144-	H146Q	F157L
		2	2	2	2	2	2	2	2	2	2	2
		Q183E	V213E	G252V	G339H	R346T	L368I	S371F	S373P	S375F	T376A	D405N
		2	2	2	2	2	2	2	2	2	2	2
		R408S	K417N	N440K	V445P	G446S	L455F	F456L	N460K	S477N	T478K	E484A
		2	2	2	2	2	2	2	2	2	2	2
		F486P	F490S	Q498R	N501Y	Y505H	D614G	H655Y	N679K	P681H	N764K	D796Y
		2	2	2	2	2	2	2	2	2	2	2
		Q954H	N969K									
		2	2									
2	HK.3.2	Q14K	T19I	L24-	P25-	P26-	A27S	Q52H	V83A	G142D	Y144-	H146Q
		2	2	2	2	2	2	2	2	2	2	2
		Q183E	V213E	G252V	G339H	R346T	L368I	S371F	S373P	S375F	T376A	D405N
		2	2	2	2	2	2	2	2	2	2	2
		R408S	K417N	N440K	V445P	G446S	L455F	F456L	N460K	S477N	T478K	E484A
		2	2	2	2	2	2	2	2	2	2	2

		F486P	F490S	Q498R	N501Y	Y505H	D614G	H655Y	N679K	P681H	N764K	D796Y
		2	2	2	2	2	2	1	2	2	2	2
		Q954H	N969K									
		2	2									
1	HK.3.9	T19I	L24-	P25-	P26-	A27S	Q52H	V83A	G142D	Y144-	H146Q	Q183E
		1	1	1	1	1	1	1	1	1	1	1
		V213E	G252V	G339H	R346T	L368I	S371F	S373P	S375F	T376A	D405N	R408S
		1	1	1	1	1	1	1	1	1	1	1
		K417N	N440K	V445P	G446S	L455F	F456L	N460K	S477N	T478K	E484A	F486P
		1	1	1	1	1	1	1	1	1	1	1
		F490S	Q498R	N501Y	Y505H	D614G	H655Y	N679K	P681H	N764K	D796Y	Q954H
		1	1	1	1	1	1	1	1	1	1	1
		N969K										
		1										
14	HK.6	T19I	L24-	P25-	P26-	A27S	Q52H	V83A	G142D	Y144-	H146Q	Q183E
		14	14	14	14	14	12	12	14	14	14	14
		V213E	G252V	D253G	G339H	R346T	L368I	S371F	S373P	S375F	T376A	D405N
		14	7	6	14	14	14	14	14	14	14	14
		R408S	K417N	N440K	V445P	G446S	F456L	N460K	S477N	T478K	E484A	F486P
		14	14	14	14	13	14	14	14	14	14	14
		F490S	Q498R	N501Y	Y505H	D614G	H655Y	N679K	P681H	N764K	D796Y	Q954H
		14	14	14	14	14	13	14	14	14	14	14
		N969K										
		14										
1	HK.11	T19I	L24-	P25-	P26-	A27S	Q52H	V83A	G142D	Y144-	H146Q	Q183E
		1	1	1	1	1	1	1	1	1	1	1
		G184S	V213E	G339H	R346T	L368I	S371F	S373P	S375F	T376A	D405N	R408S
		1	1	1	1	1	1	1	1	1	1	1
		K417N	N440K	V445P	G446S	F456L	N460K	S477N	T478K	E484A	F486P	F490S
		1	1	1	1	1	1	1	1	1	1	1
		Q498R	N501Y	Y505H	D614G	H655Y	N679K	P681H	N764K	D796Y	Q954H	N969K

		1	1	1	1	1	1	1	1	1	1	1
18	HK.15	T19I	L24-	P25-	P26-	A27S	Q52H	V83A	G142D	Y144-	H146Q	Q183E
		18	18	18	18	18	17	18	17	18	18	18
		V213E	G252V	A262T	G339H	R346T	L368I	S371F	S373P	S375F	T376A	D405N
		18	11	14	18	18	18	18	18	18	18	18
		R408S	K417N	N440K	V445P	G446S	F456L	N460K	S477N	T478K	E484A	F486P
		18	18	18	18	18	18	18	18	18	18	18
		F490S	Q498R	N501Y	Y505H	D614G	H655Y	N679K	P681H	N764K	D796Y	Q954H
		18	18	18	18	18	17	18	18	18	18	18
		N969K										
		18										
4	HK.26	T19I	L24-	P25-	P26-	A27S	Q52H	V83A	G142D	Y144-	H146Q	Q183E
		4	4	4	4	4	4	4	4	4	4	4
		V213E	G252V	G339H	R346T	L368I	S371F	S373P	S375F	T376A	D405N	R408S
		4	3	4	4	4	4	4	4	4	4	4
		K417N	N440K	V445P	G446S	L455F	F456L	N460K	S477N	T478K	E484A	F486P
		4	4	4	4	4	4	4	4	4	4	4
		F490S	Q498R	N501Y	Y505H	D614G	H655Y	N679K	P681H	N764K	D796Y	Q954H
		4	4	4	4	4	4	4	4	4	4	4
		N969K										
		4										
11	EG.5.1.3	T19I	L24-	P25-	P26-	A27S	Q52H	V83A	G142D	Y144-	H146Q	Q183E
		11	11	11	11	11	11	11	11	11	11	11
		V213E	G252V	G339H	R346T	L368I	S371F	S373P	S375F	T376A	D405N	R408S
		11	9	11	11	11	10	10	10	10	11	11
		K417N	N440K	V445P	G446S	F456L	N460K	S477N	T478K	E484A	F486P	F490S
		11	11	11	11	11	11	11	11	11	11	11
		Q498R	N501Y	Y505H	D614G	H655Y	N679K	P681H	N764K	D796Y	Q954H	N969K
		11	11	11	11	11	11	11	11	11	11	11
1	JG.2	T19I	L24-	P25-	P26-	A27S	Q52H	G75V	V83A	G142D	Y144-	H146Q
		1	1	1	1	1	1	1	1	1	1	1

		Q183E	V213E	G252V	G339H	R346T	L368I	S371F	S373P	S375F	T376A	D405N
		1	1	1	1	1	1	1	1	1	1	1
		R408S	K417N	N440K	V445P	G446S	F456L	N460K	S477N	T478K	E484A	F486P
		1	1	1	1	1	1	1	1	1	1	1
		F490S	Q498R	N501Y	Y505H	D614G	H655Y	N679K	N764K	D796Y	Q954H	N969K
		1	1	1	1	1	1	1	1	1	1	1
14	JG.3	T19I	L24-	P25-	P26-	A27S	Q52H	V83A	G142D	Y144-	H146Q	Q183E
		14	14	14	14	14	14	14	14	14	14	14
		V213E	G252V	G339H	R346T	L368I	S371F	S373P	S375F	T376A	D405N	R408S
		14	14	14	14	14	14	14	14	14	14	14
		K417N	N440K	V445P	G446S	L455F	F456L	N460K	S477N	T478K	E484A	F486P
		14	14	14	14	14	14	14	14	14	14	14
		F490S	Q498R	N501Y	Y505H	D614G	H655Y	N679K	P681H	S704L	N764K	D796Y
		14	14	14	14	14	14	14	14	14	14	14
		Q954H	N969K									
		14	14									
1	EG.5.1.4	T19I	L24-	P25-	P26-	A27S	Q52H	V83A	G142D	Y144-	H146Q	Q183E
		1	1	1	1	1	1	1	1	1	1	1
		V213E	G252V	G339H	R346T	L368I	S371F	S373P	S375F	T376A	D405N	R408S
		1	1	1	1	1	1	1	1	1	1	1
		K417N	N440K	V445P	G446S	F456L	N460K	S477N	T478K	E484A	F486P	F490S
		1	1	1	1	1	1	1	1	1	1	1
		Q498R	N501Y	Y505H	D614G	H655Y	N679K	P681H	N764K	D796Y	Q954H	N969K
		1	1	1	1	1	1	1	1	1	1	1
12	EG.5.1.6	T19I	L24-	P25-	P26-	A27S	Q52H	V83A	G142D	Y144-	H146Q	F157L
		12	12	12	12	12	12	12	12	11	8	12
		Q183E	V213E	G252V	G339H	R346T	L368I	S371F	S373P	S375F	T376A	D405N
		12	12	9	12	12	12	12	11	12	12	12
		R408S	K417N	N440K	V445P	G446S	F456L	N460K	S477N	T478K	E484A	F486P
		12	12	12	12	12	12	12	12	12	12	12
		F490S	Q498R	N501Y	Y505H	D614G	H655Y	N679K	P681H	N764K	D796Y	Q954H

		12	12	12	12	12	11	12	12	11	12	12
		N969K										
		12										
19	HV.1	T19I	L24-	P25-	P26-	A27S	Q52H	V83A	G142D	Y144-	H146Q	F157L
		19	19	19	19	19	19	19	19	19	18	19
		Q183E	V213E	G252V	G339H	R346T	L368I	S371F	S373P	S375F	T376A	D405N
		19	19	19	19	18	18	18	18	18	18	19
		R408S	K417N	N440K	V445P	G446S	L452R	F456L	N460K	S477N	T478K	E484A
		19	19	19	19	19	18	19	19	19	19	19
		F486P	F490S	Q498R	N501Y	Y505H	D614G	H655Y	N679K	P681H	N764K	D796Y
		19	19	19	19	19	19	18	19	19	19	19
		Q954H	N969K									
		19	19									
2	HV.1.1	T19I	L24-	P25-	P26-	A27S	Q52H	V83A	G142D	Y144-	H146Q	F157L
		2	2	2	2	2	2	2	2	2	2	2
		Q183E	V213E	G252V	G339H	R346T	L368I	S371F	S373P	S375F	T376A	D405N
		2	2	2	2	2	2	2	2	2	2	2
		R408S	K417N	N440K	K444T	V445P	G446S	L452R	F456L	N460K	S477N	T478K
		2	2	2	2	2	2	2	2	2	2	2
		E484A	F486P	F490S	Q498R	N501Y	Y505H	D614G	H655Y	N679K	P681H	N764K
		2	2	2	2	2	2	2	2	2	2	2
		D796Y	Q954H	N969K								
		2	2	2								
1	EG.5.1.7	T19I	L24-	P25-	P26-	A27S	Q52H	V83A	G142D	Y144-	H146Q	Q183E
		1	1	1	1	1	1	1	1	1	1	1
		V213E	G252V	K278N	G339H	R346T	L368I	S371F	S373P	S375F	T376A	D405N
		1	1	1	1	1	1	1	1	1	1	1
		R408S	K417N	N440K	V445P	G446S	F456L	N460K	S477N	T478K	E484A	F486P
		1	1	1	1	1	1	1	1	1	1	1
		F490S	Q498R	N501Y	Y505H	D614G	H655Y	N679K	P681H	N764K	D796Y	Q954H
		1	1	1	1	1	1	1	1	1	1	1

		N969K										
		1										
1	JR.1.1	T19I	L24-	P25-	P26-	A27S	Q52H	V83A	G142D	Y144-	H146Q	K182Q
		1	1	1	1	1	1	1	1	1	1	1
		Q183E	V213E	G252V	G339H	R346T	L368I	S371F	S373P	S375F	T376A	D405N
		1	1	1	1	1	1	1	1	1	1	1
		R408S	K417N	N440K	V445P	G446S	L455F	F456L	N460K	S477N	T478R	E484A
		1	1	1	1	1	1	1	1	1	1	1
		F486P	F490S	Q498R	N501Y	Y505H	D614G	H655Y	N679K	P681H	N764K	D796Y
		1	1	1	1	1	1	1	1	1	1	1
		Q954H	N969K									
		1	1									
20	EG.6.1	T19I	L24-	P25-	P26-	A27S	V83A	G142D	Y144-	H146Q	Q183E	V213E
		20	20	20	20	20	19	20	20	20	20	20
		G252V	G339H	R346T	L368I	S371F	S373P	S375F	T376A	D405N	R408S	K417N
		13	20	20	20	20	20	20	20	20	20	20
		N440K	V445P	G446S	F456L	N460K	S477N	T478K	E484A	F486P	F490S	Q498R
		20	20	20	20	20	20	20	20	20	20	20
		N501Y	Y505H	D614G	H655Y	N679K	P681H	N764K	D796Y	Q954H	N969K	
		20	20	20	20	20	20	20	20	20	20	
2	EG.10.1.1	S:T19I	S:L24-	S:P25-	S:P26-	S:A27S	S:V83A	S:G142D	S:Y144-	S:H146Q	S:Q183E	S:V213E
		2	2	2	2	2	2	2	2	2	2	2
		S:G252V	S:G339H	S:A344T	S:R346T	S:L368I	S:S371F	S:S373P	S:S375F	S:T376A	S:R403K	S:D405N
		1	2	2	2	2	2	2	2	2	2	2
		S:R408S	S:K417N	S:N440K	S:V445P	S:G446S	S:N460K	S:S477N	S:T478K	S:E484A	S:F486P	S:F490S
		2	2	1	2	2	2	2	2	2	2	2
		S:S494P	S:Q498R	S:N501Y	S:Y505H	S:D614G	S:H655Y	S:N679K	S:P681H	S:A701V	S:N764K	S:D796Y
		2	2	2	2	2	2	2	2	2	2	2
		S:Q954H	S:N969K									
		2	2									
1	EG.13	T19I	L24-	P25-	P26-	A27S	V83A	G142D	Y144-	H146Q	Q183E	V213E

		1	1	1	1	1	1	1	1	1	1	1
		G252V	G339H	R346T	L368I	S371F	S373P	S375F	T376A	D405N	R408S	K417N
		1	1	1	1	1	1	1	1	1	1	1
		N440K	V445P	G446S	N460K	S477N	T478K	E484A	F486P	F490S	Q498R	N501Y
		1	1	1	1	1	1	1	1	1	1	1
		Y505H	D614G	H655Y	N679K	P681H	N764K	D796Y	Q954H	N969K		
		1	1	1	1	1	1	1	1	1		
1	EG.14	T19I	L24-	P25-	P26-	A27S	V83A	G142D	Y144-	H146Q	Q183E	V213E
		1	1	1	1	1	1	1	1	1	1	1
		G252V	G339H	R346T	L368I	S371F	S373P	S375F	T376A	D405N	R408S	K417N
		1	1	1	1	1	1	1	1	1	1	1
		N440K	V445P	G446S	N460K	S477N	T478K	E484A	F486P	F490S	Q498R	N501Y
		1	1	1	1	1	1	1	1	1	1	1
		Y505H	D614G	H655Y	N679K	P681H	N764K	D796Y	Q954H	N969K		
		1	1	1	1	1	1	1	1	1		
7	XBB.1.16	T19I	L24-	P25-	P26-	A27S	V83A	G142D	Y144-	H146Q	E180V	Q183E
		7	7	7	7	7	7	7	7	7	7	7
		V213E	G252V	G339H	R346T	L368I	S371F	S373P	S375F	T376A	D405N	R408S
		7	3	7	7	7	7	7	7	7	7	7
		K417N	N440K	V445P	G446S	N460K	S477N	T478R	E484A	F486P	F490S	Q498R
		7	7	7	7	7	7	7	7	7	7	7
		N501Y	Y505H	D614G	H655Y	N679K	P681H	N764K	D796Y	Q954H	N969K	
		7	7	7	7	7	7	7	7	7	7	
11	XBB.1.16.1	T19I	L24-	P25-	P26-	A27S	V83A	G142D	Y144-	H146Q	E180V	Q183E
		11	11	11	11	11	11	11	11	11	11	11
		V213E	G252V	G339H	R346T	L368I	S371F	S373P	S375F	T376A	D405N	R408S
		11	7	11	11	11	11	11	11	11	11	11
		K417N	N440K	V445P	G446S	N460K	S477N	T478R	E484A	F486P	F490S	Q498R
		11	11	11	11	11	11	11	11	11	11	11
		N501Y	Y505H	T547I	D614G	H655Y	N679K	P681H	N764K	D796Y	Q954H	N969K
		11	11	11	11	11	11	11	11	11	11	11

1	FU.1	T19I	L24-	P25-	P26-	A27S	V83A	G142D	Y144-	H146Q	E180V	Q183E
		1	1	1	1	1	1	1	1	1	1	1
		V213E	G339H	R346T	L368I	S371F	S373P	S375F	T376A	D405N	R408S	K417N
		1	1	1	1	1	1	1	1	1	1	1
		N440K	V445P	G446S	N460K	S477N	T478R	E484A	F486P	F490S	Q498R	N501Y
		1	1	1	1	1	1	1	1	1	1	1
		Y505H	T547I	D614G	H655Y	N679K	P681H	N764K	D796Y	Q954H	N969K	
		1	1	1	1	1	1	1	1	1	1	
3	FU.2.1	T19I	L24-	P25-	P26-	A27S	V83A	G142D	Y144-	H146Q	E180V	Q183E
		3	3	3	3	3	3	3	3	3	3	3
		V213E	G252V	G339H	R346T	L368I	S371F	S373P	S375F	T376A	D405N	R408S
		3	1	3	3	3	3	3	3	3	3	3
		K417N	N440K	V445P	G446S	N460K	S477N	T478R	E484A	F486P	F490S	Q498R
		3	3	3	3	3	3	3	3	3	3	3
		N501Y	Y505H	T547I	D614G	E619Q	H655Y	N679K	P681H	N764K	D796Y	Q954H
		3	3	3	3	3	3	3	3	3	3	3
		N969K										
		3										
1	FU.5	T19I	L24-	P25-	P26-	A27S	V83A	K97E	G142D	Y144-	H146Q	E180V
		1	1	1	1	1	1	1	1	1	1	1
		Q183E	V213E	G252V	G339H	R346T	L368I	S371F	S373P	S375F	T376A	D405N
		1	1	1	1	1	1	1	1	1	1	1
		R408S	K417N	N440K	V445P	G446S	N460K	S477N	T478R	E484A	F486P	F490S
		1	1	1	1	1	1	1	1	1	1	1
		Q498R	N501Y	Y505H	T547I	D614G	H655Y	N679K	P681H	N764K	D796Y	Q954H
		1	1	1	1	1	1	1	1	1	1	1
		N969K										
		1										
3	XBB.1.16.2	T19I	L24-	P25-	P26-	A27S	V83A	G142D	Y144-	H146Q	E180V	Q183E
		3	3	3	3	3	3	3	3	3	3	3
		V213E	G252V	G339H	R346T	L368I	S371F	S373P	S375F	T376A	D405N	R408S

		3	1	3	3	3	3	3	3	3	3	3
		K417N	N440K	V445P	G446S	N460K	S477N	T478R	E484A	F486P	F490S	Q498R
		3	3	3	3	3	3	3	3	3	3	3
		N501Y	Y505H	D614G	H655Y	N679K	P681H	N764K	D796Y	Q954H	N969K	
		3	3	2	2	3	3	3	3	3	3	
3	GY.1	T19I	L24-	P25-	P26-	A27S	V83A	L84I	G142D	Y144-	H146Q	E180V
		3	3	3	3	3	3	3	3	3	3	3
		Q183E	V213E	G252V	G339H	R346T	L368I	S371F	S373P	S375F	T376A	D405N
		3	3	3	3	3	3	3	3	3	3	3
		R408S	K417N	N440K	V445P	G446S	N460K	S477N	T478R	E484A	F486P	F490S
		3	3	3	3	3	3	3	3	3	3	3
		Q498R	N501Y	Y505H	D614G	H655Y	N679K	P681H	N764K	D796Y	Q954H	N969K
		3	3	3	3	2	3	3	3	3	3	3
3	XBB.1.16.6	T19I	L24-	P25-	P26-	A27S	V83A	G142D	Y144-	H146Q	E180V	Q183E
		3	3	3	3	3	3	3	3	3	3	3
		V213E	G252V	G339H	R346T	L368I	S371F	S373P	S375F	T376A	D405N	R408S
		3	3	3	3	3	3	3	3	3	3	3
		K417N	N440K	V445P	G446S	F456L	N460K	S477N	T478R	E484A	F486P	F490S
		3	3	3	3	3	3	3	3	3	3	3
		Q498R	N501Y	Y505H	D614G	H655Y	N679K	P681H	N764K	D796Y	Q954H	N969K
		3	3	3	3	3	3	3	3	3	3	3
3	JF.1.1	T19I	L24-	P25-	P26-	A27S	V83A	G142D	Y144-	H146Q	E180V	Q183E
		3	3	3	3	3	3	3	3	3	3	3
		V213E	G252V	G339H	R346T	L368I	S371F	S373P	S375F	T376A	D405N	R408S
		3	2	3	3	3	3	3	3	3	3	3
		K417N	N440K	V445P	G446S	L455F	F456L	N460K	S477N	T478R	E484A	F486P
		3	3	3	3	3	3	3	3	3	3	3
		F490S	Q498R	N501Y	Y505H	D614G	H655Y	N679K	P681H	N764K	D796Y	Q954H
		3	3	3	3	2	2	3	3	3	3	3
		N969K										
		3										

1	JF.4	T19I	L24-	P25-	P26-	A27S	V83A	G142D	Y144-	E180V	Q183E	V213E
		1	1	1	1	1	1	1	1	1	1	1
		G252V	G339H	R346T	L368I	S371F	S373P	S375F	T376A	D405N	R408S	K417N
		1	1	1	1	1	1	1	1	1	1	1
		N440K	V445P	G446S	F456L	N460K	S477N	T478R	E484A	F486P	F490S	Q498R
		1	1	1	1	1	1	1	1	1	1	1
		N501Y	Y505H	D614G	H655Y	N679K	P681H	N764K	D796Y	Q954H	N969K	
		1	1	1	1	1	1	1	1	1	1	
1	XBB.1.16.10	T19I	L24-	P25-	P26-	A27S	V83A	G142D	Y144-	H146Q	E180V	Q183E
		1	1	1	1	1	1	1	1	1	1	1
		V213E	G252V	G339H	R346T	L368I	S371F	S373P	S375F	T376A	D405N	R408S
		1	1	1	1	1	1	1	1	1	1	1
		K417N	N440K	V445P	G446S	N460K	S477N	T478R	E484A	F486P	F490S	Q498R
		1	1	1	1	1	1	1	1	1	1	1
		N501Y	Y505H	D614G	H655Y	N679K	P681H	N764K	D796Y	Q954H	N969K	
		1	1	1	1	1	1	1	1	1	1	
14	XBB.1.16.11	T19I	L24-	P25-	P26-	A27S	V83A	G142D	Y144-	H146Q	E180V	Q183E
		14	14	14	14	14	14	13	13	14	14	14
		V213E	G252V	G339H	R346T	L368I	S371F	S373P	S375F	T376A	D405N	R408S
		14	12	14	14	14	14	14	14	14	14	13
		K417N	N440K	V445P	G446S	N460K	S477N	T478R	E484A	F486P	F490S	Q498R
		13	14	14	14	14	14	14	14	14	14	14
		N501Y	Y505H	P521T	D614G	H655Y	N679K	P681H	N764K	D796Y	Q954H	N969K
		14	14	14	14	14	14	14	14	14	14	14
9	XBB.1.16.13	T19I	L24-	P25-	P26-	A27S	V83A	G142D	Y144-	H146Q	E180V	Q183E
		9	9	9	9	9	9	9	9	9	9	9
		V213E	G252V	G339H	R346T	L368I	S371F	S373P	S375F	T376A	D405N	R408S
		9	9	9	9	9	9	9	9	9	9	9
		K417N	N440K	V445P	G446S	N460K	S477N	T478R	E484A	F486P	F490S	Q498R
		9	9	9	9	9	9	9	9	9	9	9
		N501Y	Y505H	D614G	H655Y	N679K	P681H	N764K	D796Y	Q954H	N969K	

		9	9	9	9	9	9	9	9	9	9	
4	HF.1	T19I	L24-	P25-	P26-	A27S	V83A	G142D	Y144-	H146Q	E180V	Q183E
		4	4	4	4	4	4	4	4	4	4	4
		V213E	G252V	K304N	G339H	R346T	L368I	S371F	S373P	S375F	T376A	D405N
		4	4	4	4	4	4	4	4	4	4	4
		R408S	K417N	N440K	V445P	G446S	N460K	S477N	T478R	E484A	F486P	F490S
		4	4	4	4	4	4	4	4	4	4	4
		Q498R	N501Y	Y505H	D614G	H655Y	N679K	P681H	N764K	D796Y	Q954H	N969K
		4	4	4	4	4	4	4	4	4	4	4
2	XBB.1.16.15	T19I	L24-	P25-	P26-	A27S	V83A	G142D	Y144-	H146Q	K147N	E180V
		2	2	2	2	2	2	2	2	2	2	2
		Q183E	V213E	G252V	G339H	R346T	L368I	S371F	S373P	S375F	T376A	D405N
		2	2	2	2	2	2	2	2	2	2	2
		R408S	K417N	N440K	V445P	G446S	N460K	S477N	T478R	E484A	F486P	F490S
		2	2	2	2	2	2	2	2	2	2	2
		Q498R	N501Y	Y505H	P521S	D614G	H655Y	N679K	P681H	N764K	D796Y	Q954H
		2	2	2	2	2	2	2	2	2	2	2
		N969K										
		2										
1	XBB.1.16.18	T19I	L24-	P25-	P26-	A27S	V83A	G142D	Y144-	H146Q	E180V	Q183E
		1	1	1	1	1	1	1	1	1	1	1
		V213E	G339H	R346T	L368I	S371F	S373P	S375F	T376A	D405N	R408S	K417N
		1	1	1	1	1	1	1	1	1	1	1
		N440K	V445P	G446S	N460K	S477N	T478R	E484A	F486P	F490S	Q498R	N501Y
		1	1	1	1	1	1	1	1	1	1	1
		Y505H	D614G	H655Y	N679K	P681H	N764K	D796Y	Q954H	N969K		
		1	1	1	1	1	1	1	1	1		
2	XBB.1.16.21	T19I	L24-	P25-	P26-	A27S	V83A	G142D	Y144-	H146Q	E180V	Q183E
		2	2	2	2	2	2	2	2	2	2	2
		V213E	G252V	G339H	R346T	L368I	S371F	S373P	S375F	T376A	D405N	R408S
		2	2	2	2	2	2	2	2	2	2	2

		K417N	N440K	V445P	G446S	N460K	S477N	T478R	E484A	F486P	F490S	Q498R
		2	2	2	2	2	2	2	2	2	2	2
		N501Y	Y505H	D614G	H655Y	N679K	P681H	N764K	D796Y	Q954H	N969K	
		2	2	2	2	2	2	2	2	2	2	
1	XBB.1.16.27	T19I	L24-	P25-	P26-	A27S	V83A	G142D	Y144-	H146Q	E180V	Q183E
		1	1	1	1	1	1	1	1	1	1	1
		V213E	G252V	G339H	R346T	L368I	S371F	S373P	S375F	T376A	D405N	R408S
		1	1	1	1	1	1	1	1	1	1	1
		K417N	N440K	V445P	G446S	F456L	N460K	S477N	T478R	E484A	F486P	F490S
		1	1	1	1	1	1	1	1	1	1	1
		Q498R	N501Y	Y505H	E554K	D614G	H655Y	N679K	P681H	N764K	D796Y	Q954H
		1	1	1	1	1	1	1	1	1	1	1
		N969K										
		1										
3	FE.1.1	T19I	L24-	P25-	P26-	A27S	V83A	G142D	Y144-	H146Q	Q183E	V213E
		3	3	3	3	3	3	3	3	2	3	3
		G252V	G339H	R346T	L368I	S371F	S373P	S375F	T376A	D405N	R408S	K417N
		2	3	3	3	3	3	3	3	3	3	3
		N440K	V445P	G446S	F456L	N460K	S477N	T478K	E484A	F486P	F490S	Q498R
		3	3	3	3	3	3	3	3	3	3	3
		N501Y	Y505H	D614G	H655Y	N679K	P681H	N764K	D796Y	Q954H	N969K	
		3	3	3	3	3	3	3	3	3	3	
2	FE.1.1.5	T19I	L24-	P25-	P26-	A27S	V83A	G142D	Y144-	H146Q	Q183E	V213E
		2	2	2	2	2	2	2	2	2	2	2
		G252V	G339H	R346T	L368I	S371F	S373P	S375F	T376A	D405N	R408S	K417N
		1	2	2	2	2	2	2	2	2	2	2
		N440K	V445P	G446S	F456L	N460K	S477N	T478K	E484A	F486P	F490S	Q498R
		2	2	2	2	2	2	2	2	2	2	2
		N501Y	Y505H	E554K	D614G	H655Y	N679K	P681H	N764K	D796Y	Q954H	N969K
		2	2	2	2	2	2	2	2	2	2	2
1	GW.4	T19I	L24-	P25-	P26-	A27S	V83A	G142D	Y144-	H146Q	Q183E	V213E

		1	1	1	1	1	1	1	1	1	1	1
		G252V	G339H	R346T	L368I	S371F	S373P	S375F	T376A	D405N	R408S	K417N
		1	1	1	1	1	1	1	1	1	1	1
		N440K	V445P	G446S	N460K	S477N	T478K	E484A	F486P	F490S	Q498R	N501Y
		1	1	1	1	1	1	1	1	1	1	1
		Y505H	E554K	D614G	H655Y	N679K	P681H	N764K	D796Y	Q954H	N969K	
		1	1	1	1	1	1	1	1	1	1	
1	XBB.1.22.1	T19I	L24-	P25-	P26-	A27S	V83A	G142D	Y144-	H146Q	Q183E	Y200C
		1	1	1	1	1	1	1	1	1	1	1
		V213E	G339H	R346T	L368I	S371F	S373P	S375F	T376A	D405N	R408S	K417N
		1	1	1	1	1	1	1	1	1	1	1
		N440K	V445P	G446S	N460K	S477N	T478K	E484A	F486P	F490S	Q498R	N501Y
		1	1	1	1	1	1	1	1	1	1	1
		Y505H	D614G	H655Y	N679K	P681H	N764K	D796Y	Q954H	N969K		
		1	1	1	1	1	1	1	1	1		
1	FY.5.1.1	T19I	L24-	P25-	P26-	A27S	V83A	G142D	Y144-	H146Q	M153I	Q183E
		1	1	1	1	1	1	1	1	1	1	1
		F186S	Y200C	V213E	S247N	G252V	G339H	R346T	L368I	S371F	S373P	S375F
		1	1	1	1	1	1	1	1	1	1	1
		T376A	D405N	R408S	K417N	N440K	V445P	G446S	N460K	S477N	T478R	E484A
		1	1	1	1	1	1	1	1	1	1	1
		F486P	F490S	Q498R	N501Y	Y505H	T572I	D614G	H655Y	N679K	P681H	N764K
		1	1	1	1	1	1	1	1	1	1	1
		D796Y	Q954H	N969K								
		1	1	1								
1	FY.5.4	T19I	L24-	P25-	P26-	A27S	V83A	G142D	Y144-	H146Q	Q183E	Y200C
		1	1	1	1	1	1	1	1	1	1	1
		V213E	G252V	G339H	R346T	L368I	S371F	S373P	S375F	T376A	D405N	R408S
		1	1	1	1	1	1	1	1	1	1	1
		K417N	N440K	V445P	G446S	N460K	S477N	T478R	E484A	F486P	F490S	Q498R
		1	1	1	1	1	1	1	1	1	1	1

		N501Y	Y505H	T572I	D614G	H655Y	N679K	P681H	N764K	D796Y	Q954H	N969K
		1	1	1	1	1	1	1	1	1	1	1
1	XBB.1.24.1	T19I	L24-	P25-	P26-	A27S	V83A	G142D	Y144-	H146Q	Q183E	V213E
		1	1	1	1	1	1	1	1	1	1	1
		G252V	G339H	R346T	L368I	S371F	S373P	S375F	T376A	D405N	R408S	K417N
		1	1	1	1	1	1	1	1	1	1	1
		N440K	V445P	G446S	N460K	S477N	T478K	E484A	F486P	F490S	Q498R	N501Y
		1	1	1	1	1	1	1	1	1	1	1
		Y505H	L518Q	D614G	H655Y	N679K	P681H	N764K	D796Y	Q954H	N969K	
		1	1	1	1	1	1	1	1	1	1	
1	XBB.1.16.28	T19I	L24-	P25-	P26-	A27S	V83A	G142D	Y144-	H146Q	E180V	Q183E
		1	1	1	1	1	1	1	1	1	1	1
		V213E	G252V	G339H	R346T	L368I	S371F	S373P	S375F	T376A	D405N	R408S
		1	1	1	1	1	1	1	1	1	1	1
		K417N	N440K	V445P	G446S	N460K	S477N	T478R	E484A	F486P	F490S	Q498R
		1	1	1	1	1	1	1	1	1	1	1
		N501Y	Y505H	Q613H	D614G	H655Y	Q675K	N679K	P681H	N764K	D796Y	Q954H
		1	1	1	1	1	1	1	1	1	1	1
		N969K	V1230A									
		1	1									
2	FW.1.1	T19I	L24-	P25-	P26-	A27S	V83A	G142D	Y144-	H146Q	N148T	Q183E
		2	2	2	2	2	2	2	2	2	2	2
		V213E	G252V	G339H	R346T	K356T	L368I	S371F	S373P	S375F	T376A	R403K
		2	2	2	2	2	2	2	2	2	2	2
		D405N	R408S	K417N	N440K	V445P	G446S	N460K	S477N	T478K	E484A	F486S
		2	2	2	2	2	2	2	2	2	2	2
		F490S	Q498R	N501Y	Y505H	D614G	H655Y	N679K	P681H	A701V	N764K	D796Y
		2	2	2	2	2	2	2	2	2	2	2
		Q954H	N969K									
		2	2									
1	XBB.1.16.31	T19I	L24-	P25-	P26-	A27S	V83A	G142D	Y144-	E180V	Q183E	V213E

		1	1	1	1	1	1	1	1	1	1	1
		G252V	G339H	R346T	L368I	S371F	S373P	S375F	T376A	D405N	R408S	K417N
		1	1	1	1	1	1	1	1	1	1	1
		N440K	V445P	G446S	N460K	S477N	T478R	E484A	F486P	F490S	Q498R	N501Y
		1	1	1	1	1	1	1	1	1	1	1
		Y505H	D614G	H655Y	N679K	P681H	N764K	D796Y	Q954H	N969K		
		1	1	1	1	1	1	1	1	1		
2	JC.2	T19I	L24-	P25-	P26-	A27S	V83A	G142D	Y144-	H146Q	Q183E	V213E
		2	2	2	2	2	2	2	2	2	2	2
		G252V	L335S	G339R	R346T	L368I	S371F	S373P	S375F	T376A	R403K	D405N
		2	2	2	2	2	2	2	2	2	2	2
		R408S	K417N	N440K	V445P	G446S	N460K	S477N	T478R	E484A	F486P	F490S
		2	2	2	2	2	2	2	2	2	2	2
		Q498R	N501Y	Y505H	D614G	H655Y	N679K	P681H	N764K	D796Y	Q954H	N969K
		2	2	2	2	1	2	2	2	2	2	2
1	XBB.1.41.2	T19I	L24-	P25-	P26-	A27S	V83A	G142D	Y144-	H146Q	Q183E	V213E
		1	1	1	1	1	1	1	1	1	1	1
		G252V	L335S	G339H	R346T	L368I	S371F	S373P	S375F	T376A	R403K	D405N
		1	1	1	1	1	1	1	1	1	1	1
		R408S	K417N	N440K	V445P	G446S	N460K	S477N	T478K	E484A	F486P	F490S
		1	1	1	1	1	1	1	1	1	1	1
		S494P	Q498R	N501Y	Y505H	D614G	H655Y	N679K	P681H	N764K	D796Y	Q954H
		1	1	1	1	1	1	1	1	1	1	1
		N969K										
		1										
11	XBB.1.42	T19I	L24-	P25-	P26-	A27S	V83A	G142D	Y144-	H146Q	Q183E	V213E
		11	11	11	11	11	11	11	11	11	11	11
		G252V	G339H	R346T	L368I	S371F	S373P	S375F	T376A	D405N	R408S	K417N
		7	11	11	11	11	11	11	11	11	11	11
		N440K	V445P	G446S	N460K	S477N	T478K	E484A	F486P	F490S	Q498R	N501Y
		11	11	11	11	11	11	11	11	11	11	11

		Y505H	Q613H	D614G	H655Y	N679K	P681H	N764K	D796Y	Q954H	N969K	
		11	11	11	11	11	11	11	11	11	11	
1	XBB.2	T19I	L24-	P25-	P26-	A27S	V83A	G142D	Y144-	H146Q	Q183E	V213E
		1	1	1	1	1	1	1	1	1	1	1
		D253G	G339H	R346T	L368I	S371F	S373P	S375F	T376A	D405N	R408S	K417N
		1	1	1	1	1	1	1	1	1	1	1
		N440K	V445P	G446S	N460K	S477N	T478K	E484A	F486S	F490S	Q498R	N501Y
		1	1	1	1	1	1	1	1	1	1	1
		Y505H	D614G	H655Y	N679K	P681H	N764K	D796Y	Q954H	N969K		
		1	1	1	1	1	1	1	1	1		
1	XBB.2.3.2	T19I	L24-	P25-	P26-	A27S	V83A	G142D	Y144-	H146Q	Q183E	G184V
		1	1	1	1	1	1	1	1	1	1	1
		V213E	D253G	G339H	R346T	L368I	S371F	S373P	S375F	T376A	D405N	R408S
		1	1	1	1	1	1	1	1	1	1	1
		K417N	N440K	V445P	G446S	N460K	S477N	T478K	E484A	F486P	F490S	Q498R
		1	1	1	1	1	1	1	1	1	1	1
		N501Y	Y505H	P521S	D614G	H655Y	N679K	P681H	N764K	D796Y	Q954H	N969K
		1	1	1	1	1	1	1	1	1	1	1
1	HH.2.1	T19I	L24-	P25-	P26-	A27S	V83A	G142D	Y144-	H146Q	Q183E	G184V
		1	1	1	1	1	1	1	1	1	1	1
		V213E	D253G	G339H	R346T	L368I	S371F	S373P	S375F	T376A	D405N	R408S
		1	1	1	1	1	1	1	1	1	1	1
		K417N	N440K	V445P	G446S	N460K	S477N	E484A	F486P	F490S	Q498R	N501Y
		1	1	1	1	1	1	1	1	1	1	1
		Y505H	P521S	D614G	H655Y	N679K	P681H	N764K	D796Y	Q954H	N969K	
		1	1	1	1	1	1	1	1	1	1	
4	XBB.2.3.3	T19I	L24-	P25-	P26-	A27S	V83A	G142D	Y144-	H146Q	K182N	Q183E
		4	4	4	4	4	4	4	4	4	4	4
		V213E	D253G	G339H	R346T	L368I	S371F	S373P	S375F	T376A	D405N	R408S
		4	4	4	4	4	4	4	4	4	4	4
		K417N	N440K	V445P	G446S	N460K	S477N	T478K	E484A	F486P	F490S	Q498R

		4	4	4	4	4	4	4	4	4	4	4
		N501Y	Y505H	P521S	D614G	H655Y	N679K	P681H	N764K	D796Y	Q954H	N969K
		4	4	4	4	4	4	4	4	4	4	4
1	GJ.1.2	T19I	L24-	P25-	P26-	A27S	V83A	G142D	Y144-	H146Q	K182N	Q183E
		1	1	1	1	1	1	1	1	1	1	1
		V213E	D253G	G339H	R346T	L368I	S371F	S373P	S375F	T376A	D405N	R408S
		1	1	1	1	1	1	1	1	1	1	1
		K417N	N440K	V445P	G446S	N460K	S477N	T478K	E484A	F486P	F490S	Q498R
		1	1	1	1	1	1	1	1	1	1	1
		N501Y	Y505H	P521S	D614G	H655Y	N679K	P681H	N764K	D796Y	Q954H	N969K
		1	1	1	1	1	1	1	1	1	1	1
2	GJ.4	T19I	L24-	P25-	P26-	A27S	V83A	G142D	Y144-	H146Q	M153I	K182N
		2	2	2	2	2	2	2	2	2	2	2
		Q183E	V213E	D253G	G339H	R346T	L368I	S371F	S373P	S375F	T376A	D405N
		2	2	2	2	2	2	2	2	2	2	2
		R408S	K417N	N440K	V445P	G446S	N460K	S477N	T478K	E484A	F486P	F490S
		2	2	2	2	2	2	2	2	2	2	2
		Q498R	N501Y	Y505H	P521S	D614G	H655Y	N679K	P681H	N764K	D796Y	Q954H
		2	2	2	2	2	2	2	2	2	2	2
		N969K										
		2										
1	GJ.5.1	T19I	L24-	P25-	P26-	A27S	V83A	G142D	Y144-	H146Q	K182N	Q183E
		1	1	1	1	1	1	1	1	1	1	1
		V213E	G339H	R346T	L368I	S371F	S373P	S375F	T376A	D405N	R408S	K417N
		1	1	1	1	1	1	1	1	1	1	1
		N440K	V445P	G446S	N460K	S477N	T478R	E484A	F486P	F490S	Q498R	N501Y
		1	1	1	1	1	1	1	1	1	1	1
		Y505H	P521S	D614G	H655Y	N679K	P681H	N764K	D796Y	Q954H	N969K	
		1	1	1	1	1	1	1	1	1	1	
7	GZ.1	L24-	P25-	P26-	A27S	V83A	G142D	Y144-	H146Q	Q183E	F186L	V213E
		7	7	7	7	7	7	7	7	7	7	7

		D253G	G339H	R346T	L368I	S371F	S373P	S375F	T376A	D405N	R408S	K417N
		5	7	7	7	7	7	7	7	7	6	6
		N440K	V445P	G446S	N460K	S477N	T478Q	E484A	F486P	F490S	Q498R	N501Y
		6	7	7	7	7	7	7	7	7	7	7
		Y505H	P521S	D614G	H655Y	N679K	P681H	N764K	D796Y	Q954H	N969K	
		7	7	7	7	7	7	7	7	7	7	
58	GE.1	T19I	L24-	P25-	P26-	A27S	V83A	G142D	Y144-	H146Q	Q183E	N185-
		58	58	58	58	58	58	56	57	55	57	57
		F186I	V213E	D253G	G339H	R346T	L368I	S371F	S373P	S375F	T376A	D405N
		58	58	48	58	58	58	58	58	58	58	58
		R408S	K417N	N440K	V445P	G446S	N460K	S477N	T478R	E484A	F486P	F490S
		57	57	58	58	58	57	58	58	58	58	58
		Q498R	N501Y	Y505H	P521S	D614G	H655Y	N679K	P681H	N764K	D796Y	Q954H
		58	58	58	58	58	58	58	58	58	58	58
		N969K										
		58										
1	GE.1.3	L24-	P25-	P26-	A27S	V83A	G142D	Y144-	H146Q	Q183E	N185-	F186I
		1	1	1	1	1	1	1	1	1	1	1
		V213E	D253G	G339H	R346T	L368I	S371F	S373P	S375F	T376A	D405N	R408S
		1	1	1	1	1	1	1	1	1	1	1
		K417N	N440K	V445P	G446S	N460K	S477N	T478R	E484A	F486P	F490S	Q498R
		1	1	1	1	1	1	1	1	1	1	1
		N501Y	Y505H	P521S	D614G	H655Y	N679K	P681H	T732I	N764K	D796Y	Q954H
		1	1	1	1	1	1	1	1	1	1	1
		N969K										
		1										
6	GE.1.5	T19I	L24-	P25-	P26-	A27S	V83A	G142D	Y144-	H146Q	Q183E	N185-
		6	6	6	6	6	6	5	6	6	6	6
		F186I	V213E	D253G	G339H	R346T	L368I	S371F	S373P	S375F	T376A	D405N
		6	6	6	6	6	6	6	6	6	6	6
		R408S	K417N	N440K	V445P	G446S	N460K	S477N	T478R	E484A	F486P	F490S

		6	6	6	6	6	6	6	6	6	6	6
		Q498R	N501Y	Y505H	P521S	D614G	H655Y	N679K	P681H	N764K	D796Y	Q954H
		6	6	6	6	6	6	6	6	6	6	6
		N969K										
		6										
2	GE.1.6	T19I	L24-	P25-	P26-	A27S	V83A	L118I	G142D	Y144-	H146Q	Q183E
		2	2	2	2	2	2	2	2	2	2	2
		N185-	F186I	V213E	D253G	G339H	R346T	L368I	S371F	S373P	S375F	T376A
		2	2	2	2	2	2	2	2	2	2	2
		D405N	R408S	K417N	N440K	V445P	G446S	N460K	S477N	T478R	E484A	F486P
		2	2	2	2	2	2	2	2	2	2	2
		F490S	Q498R	N501Y	Y505H	P521S	D614G	H655Y	N679K	P681H	N764K	D796Y
		2	2	2	2	2	2	2	2	2	2	2
		Q954H	N969K									
		2	2									
1	GS.1	T19I	L24-	P25-	P26-	A27S	V83A	G142D	Y144-	H146Q	Q183E	V213D
		1	1	1	1	1	1	1	1	1	1	1
		D253G	G339H	R346T	L368I	S371F	S373P	S375F	T376A	D405N	R408S	K417N
		1	1	1	1	1	1	1	1	1	1	1
		N440K	V445P	G446S	N460K	S477N	T478R	E484A	F486P	F490S	Q498R	N501Y
		1	1	1	1	1	1	1	1	1	1	1
		Y505H	P521S	D614G	H655Y	N679K	P681H	N764K	D796Y	Q954H	N969K	
		1	1	1	1	1	1	1	1	1	1	
13	GS.4.1	T19I	L24-	P25-	P26-	A27S	V83A	G142D	Y144-	H146Q	Q183E	N185D
		13	13	13	13	13	13	13	13	13	13	11
		V213E	D253G	G339H	R346T	L368I	S371F	S373P	S375F	T376A	D405N	R408S
		13	13	13	13	13	13	13	13	13	13	13
		K417N	N440K	V445P	G446S	N460K	S477N	T478R	E484A	F486P	F490S	Q498R
		13	13	13	13	13	13	13	13	13	13	13
		N501Y	Y505H	P521S	D614G	H655Y	N679K	P681H	N764K	D796Y	Q954H	N969K
		13	13	13	13	13	13	13	13	13	13	13

2	GS.4.1.1	T19I	L24-	P25-	P26-	A27S	V83A	G142D	Y144-	H146Q	Q183E	N185D
		2	2	2	2	2	2	2	2	2	2	2
		V213E	D253G	G339H	R346T	L368I	S371F	S373P	S375F	T376A	D405N	R408S
		2	2	2	2	2	2	2	2	2	2	2
		K417N	N440K	V445P	G446S	N460K	S477N	T478R	E484A	F486P	F490S	Q498R
		2	2	2	2	2	2	2	2	2	1	2
		N501Y	Y505H	P521S	D614G	H655Y	N679K	P681H	N764K	D796Y	Q954H	N969K
		2	2	2	2	2	2	2	2	2	2	2
2	XBB.3.2	L24-	P25-	P26-	A27S	V83A	G142D	Y144-	H146Q	M177T	Q183E	V213E
		2	2	2	2	2	2	2	2	2	2	2
		G339H	R346T	L368I	S371F	S373P	S375F	T376A	D405N	R408S	K417N	N440K
		2	2	2	2	2	2	2	2	2	2	2
		V445P	G446S	N460K	S477N	T478K	E484A	F486S	F490S	Q498R	N501Y	Y505H
		2	2	2	2	2	2	2	2	2	2	2
		D614G	H655Y	N679K	P681H	N764K	D796Y	Q954H	N969K			
		2	2	2	2	2	2	2	2			
1	XAY.1.1.2	L18F	T19R	R21G	T95I	G142D	R158G	F186L	V213G	D253G	G339D	R346T
		1	1	1	1	1	1	1	1	1	1	1
		S371F	S373P	S375F	T376A	D405N	R408S	K417N	N440K	G446D	L452R	S477N
		1	1	1	1	1	1	1	1	1	1	1
		T478K	E484A	F486P	Q498R	N501Y	Y505H	D614G	P621S	H655Y	N679K	P681H
		1	1	1	1	1	1	1	1	1	1	1
		A706V	N764K	D796Y	Q954H	N969K	T1117I					
		1	1	1	1	1	1					
1	XBF	T19I	L24-	P25-	P26-	A27S	G142D	K147E	W152R	F157L	I210V	V213G
		1	1	1	1	1	1	1	1	1	1	1
		G257S	G339H	R346T	S371F	S373P	S375F	T376A	D405N	R408S	K417N	N440K
		1	1	1	1	1	1	1	1	1	1	1
		G446S	N460K	S477N	T478K	E484A	F486P	F490S	Q498R	N501Y	Y505H	D614G
		1	1	1	1	1	1	1	1	1	1	1
		H655Y	N679K	P681H	N764K	D796Y	Q954H	N969K				

		1	1	1	1	1	1	1				
4	XBF.7	T19I	L24-	P25-	P26-	A27S	G142D	K147E	W152R	F157L	I210V	V213G
		4	4	4	4	4	4	4	4	4	4	4
		G257S	G339H	R346T	S371F	S373P	S375F	T376A	D405N	R408S	K417N	N440K
		4	4	4	4	4	4	4	4	4	4	4
		G446S	N460K	S477N	T478K	E484A	F486P	F490S	Q498R	N501Y	Y505H	D614G
		4	4	4	4	4	4	4	4	4	4	4
		H655Y	N679K	P681H	N764K	D796Y	Q954H	N969K				
		4	4	4	4	4	4	4				
3	XBF.7.1	T19I	L24-	P25-	P26-	A27S	G142D	K147E	W152R	F157L	I210V	V213G
		3	3	3	3	3	3	3	3	3	3	3
		G257S	T323I	G339H	R346T	S371F	S373P	S375F	T376A	D405N	R408S	K417N
		3	3	3	3	3	3	3	3	3	3	3
		N440K	G446S	N460K	S477N	T478K	E484A	F486P	F490S	Q498R	N501Y	Y505H
		3	3	3	3	3	3	3	3	3	3	3
		D614G	H655Y	N679K	P681H	N764K	D796Y	Q954H	N969K			
		3	3	3	3	3	3	3	3			
8	XBU	T19I	L24-	P25-	P26-	A27S	G142D	K147E	W152R	F157L	I210V	V213G
		8	8	8	8	8	8	8	8	8	8	8
		G257S	G339H	R346T	S371F	S373P	S375F	T376A	D405N	R408S	K417N	N440K
		8	8	8	8	8	8	8	8	8	8	8
		K444T	L452R	N460K	S477N	T478K	E484A	F486V	Q498R	N501Y	Y505H	D614G
		8	8	8	8	8	8	8	8	8	8	8
		H655Y	N679K	P681H	N764K	D796Y	Q954H	N969K				
		8	8	8	8	8	8	8				
1	XCA	T19I	L24-	P25-	P26-	A27S	D111N	G142D	K147E	W152R	F157L	I210V
		1	1	1	1	1	1	1	1	1	1	1
		V213G	G257S	G339H	R346T	S371F	S373P	S375F	T376A	D405N	R408S	K417N
		1	1	1	1	1	1	1	1	1	1	1
		N440K	K444T	L452R	N460K	S477N	T478K	E484A	F486V	Q498R	N501Y	Y505H
		1	1	1	1	1	1	1	1	1	1	1

		D614G	H655Y	T678I	N679K	P681H	N764K	D796Y	Q954H	N969K		
		1	1	1	1	1	1	1	1	1		
		T19I	L24-	P25-	P26-	A27S	V83A	G142D	Y144-	H146Q	Q183E	V213E
		1	1	1	1	1	1	1	1	1	1	1
		G339H	R346T	L368I	S371F	S373P	S375F	T376A	D405N	R408S	K417N	N440K
		1	1	1	1	1	1	1	1	1	1	1
1	XCH.1	V445P	G446S	L455F	F456L	N460K	S477N	T478K	E484A	F486P	F490S	Q498R
		1	1	1	1	1	1	1	1	1	1	1
		N501Y	Y505H	T573I	D614G	H655Y	N679K	P681H	S704L	N764K	D796Y	Q954H
		1	1	1	1	1	1	1	1	1	1	1
		N969K										
		1										
		T19I	L24-	P25-	P26-	A27S	V83A	G142D	Y144-	H146Q	L176F	E180V
		4	4	4	4	4	4	4	4	4	4	4
		K182Q	Q183E	V213E	G252V	G339H	R346T	L368I	S371F	S373P	S375F	T376A
		4	4	4	4	4	4	4	4	4	4	4
4	XDA.1	D405N	R408S	K417N	N440K	V445P	G446S	F456L	N460K	S477N	T478R	E484A
		4	4	4	4	4	4	4	4	4	4	4
		F486P	F490S	Q498R	N501Y	Y505H	E554K	D614G	H655Y	N679K	P681H	A701V
		4	4	4	4	4	4	4	4	4	4	4
		N764K	D796Y	Q954H	N969K							
		4	4	4	4							
		T19I	R21T	L24-	P25-	P26-	A27S	S50L	H69-	V70-	V127F	G142D
		2	2	2	2	2	2	2	2	2	2	2
		Y144-	F157S	R158G	N211-	L212I	V213G	L216F	H245N	A264D	I332V	G339H
		2	2	2	2	2	2	2	2	2	2	2
2	XDD	K356T	S371F	S373P	S375F	T376A	R403K	D405N	R408S	K417N	N440K	V445H
		2	2	2	2	2	2	2	2	2	2	2
		G446S	N450D	L452W	L455S	N460K	S477N	T478K	N481K	V483-	E484K	F486P
		2	2	2	2	2	2	2	2	2	2	2
		Q498R	N501Y	Y505H	E554K	A570V	D614G	P621S	H655Y	N679K	P681R	N764K

		2	2	2	2	2	2	2	2	2	2	2
		D796Y	S939F	Q954H	N969K	P1143L						
		2	2	2	2	2						
1	XDD.1	S50L	H69-	V70-	V127F	G142D	Y144-	F157S	R158G	N211-	L212I	V213G
		1	1	1	1	1	1	1	1	1	1	1
		L216F	H245N	A264D	I332V	G339H	K356T	S371F	S373P	S375F	T376A	R403K
		1	1	1	1	1	1	1	1	1	1	1
		D405N	R408S	K417N	N440K	V445H	G446S	N450D	L452W	L455S	N460K	S477N
		1	1	1	1	1	1	1	1	1	1	1
		T478K	N481K	V483-	E484K	F486P	Q498R	N501Y	Y505H	E554K	A570V	D614G
		1	1	1	1	1	1	1	1	1	1	1
		P621S	H655Y	N679K	P681R	S704L	N764K	D796Y	S939F	Q954H	N969K	P1143L
		1	1	1	1	1	1	1	1	1	1	1
1	XDK	S50L	H69-	V70-	V127F	G142D	Y144-	F157S	R158G	N211-	L212I	V213G
		1	1	1	1	1	1	1	1	1	1	1
		L216F	H245N	A264D	I332V	G339H	K356T	S371F	S373P	S375F	T376A	R403K
		1	1	1	1	1	1	1	1	1	1	1
		D405N	R408S	K417N	N440K	V445H	G446S	N450D	L452W	L455S	N460K	S477N
		1	1	1	1	1	1	1	1	1	1	1
		T478K	N481K	V483-	E484K	F486P	Q498R	N501Y	Y505H	E554K	A570V	T572I
		1	1	1	1	1	1	1	1	1	1	1
		D614G	P621S	H655Y	N679K	P681R	N764K	D796Y	S939F	Q954H	N969K	P1143L
		1	1	1	1	1	1	1	1	1	1	1

[a]The most prevalent mutations shown in Table S1 were determined in accordance to the most common mutations of the lineages in this dataset, and as denoted by CoV-Spectrum (http//cov-spectrum.org/)(date last accessed 10 July 2024) [81].

[b]Total number of sequences that were identified for each lineage in this dataset.

[c]For lineages that were identified in lower frequency in this dataset, the CoV-Spectrum (http//cov-spectrum.org/)(date last accessed 10 July 2024) [81].

[d]The mutations that were listed as above 75% prevalence in CoV-Spectrum http//cov-spectrum.org/)(date last accessed 10 July 2024), but were found in lower frequencies (below 75%) in this dataset for specific lineages were also indicated [81].

Table S2. Uncommon S-protein Mutations of Omicron Variants that were Identified in this Dataset[a].

Total Sequences[b]	Lineage[c]	Mutations and Number of Sequences Identified for Each Mutation[d]					
3	BA.2.3.20	V1268I					
		1					
16	CH.1.1	L5F	L54F	F643L	A892S	D1153H	Y1215H
		1	7	1	1	1	2
3	CH.1.1.1	T299I	R403K	T478N			
		1	1	1			
5	DV.6	G339R	T385I				
		1	2				
2	DV.7.1.3	T393A	L1224F				
		1	1				
8	DV.7.1.4	A706V					
		4					
4	BN.1	T307I	T572I				
		1	1				
29	BN.1.3	D253G	G482-	K1266R			
		1	1	4			
9	BN.1.3.1	Y453H					
		1					
5	BN.1.5	I68M	T307I				
		4	1				
4	BN.3.1	C1248F					
		4					
1	BA.2.86	ins16MPLF					
		1					
26	BA.2.86.1	ins16MPLF	A260T	L455S	T678I	P681H	L1224F
		2	1	2	1	1	1

188	JN.1	L5F	P9S	ins16MPLF	S46L	H49Y	Q52H	V83A	A123V	E154D	G184S	N185S
		1	1	19	3	2	2	1	1	1	2	1
		G219S	S221L	T307I	E484A	T573I	Q613H	T678I	R683Q	S689I	T859N	T961M
		2	1	9	1	1	1	1	1	1	1	1
		M1050I	H1083R	V1133F	D1146H	K1191N	G1219C	L1224F	M1237I			
		1	1	1	3	3	2	6	3			
66	JN.1.1	Q52H	V83A	N481-	G482K	V483D	D614S	S680P	A688V	L1224F	G1246C	
		1	1	8	8	8	1	1	1	4	6	
6	JN.1.2	S31F	D1084Y									
		2	1									
60	JN.1.4	ins16MPLF	D111N	N148T	S221L	T376S	R634H	A845S	L1224F			
		3	1	1	1	1	1	2	1			
1	JN.1.5	V483-	E484K									
		1	1									
1	JN.1.7	R21T										
		1										
3	JN.1.16	N185S										
		1										
10	JN.2	N481E	N487T									
		1	1									
6	JN.3	L1224F										
		1										
4	JN.6	A475V										
		4										
28	JN.10	ins16MPLF	S221L	V227I	T415S	S680F	L1224F					
		2	1	1	1	1	10					
4	BA.5.1	T573I	I624V	D950G	P1079T							
		1	2	1	1							
4	BA.5.1.10	N450D										
		4										
13	BA.5.2	D138N	N450D	A942S	K1073T							

		2	4	2	1	
20	BA.5.2.1	L5F	S71F	P82H	A435S	S691F
		1	3	3	3	2
13	BF.5	S640F				
		1				
16	BF.7	L5F	Y269H	A653V	S939F	
		1	1	1	1	
9	BF.7.1	A222S				
		1				
6	BF.7.4.1	A263V				
		1				
1	BF.7.6	E1262V				
		1				
2	BF.7.7	S255F				
		2				
6	BF.7.8	C15R				
		1				
1	BF.7.20	I68-	V70T	Y200C	Q564E	
		1	1	1	1	
1	BF.7.22	T500A				
		1				
2	BF.7.23	K356T	T547I			
		1	1			
1	BF.7.24	Y269H				
		1				
1	BF.10	P272L				
		1				
17	BF.11	R78S	N460Y	T547I	N1023Y	T1136I
		1	1	1	4	1
14	BF.11.2	G75V				
		1				

11	BF.40	E661D	R1091H									
		1	1									
5	BA.5.2.20	S98F	R346T	V642G	D1146N							
		2	1	2	3							
1	CK.2	T547K										
		1										
2	BA.5.2.26	R346I	V1264L									
		1	1									
15	BA.5.2.34	Y144-	D215Y	V445F	I670V	E780K	S810A					
		1	2	1	1	1	1					
1	DQ.1	V1094A										
		1										
3	BQ.1	Y144-	S221L									
		2	1									
95	BQ.1.1	I68-	G75D	R78M	Y144-	N211-	P209S	L212I	P322S	T323I	E324K	G339H
		1	1	1	24	1	8	1	1	1	1	1
		T345I	T385I	T547I	K558M	E619Q	E661D	T678I	A831V	S939F	M1050I	H1083Q
		1	7	1	1	1	1	1	2	9	12	1
		G1124V	P1263S									
		1	8									
1	BQ.1.1.45	S494P										
		1										
1	BQ.1.1.47	W152L										
		1										
1	BQ.1.1.66	T19L										
		1										
13	BQ.1.1.79	S98F	Y144-	A852S	H1058Y	D1163Y						
		1	8	5	1	1						
5	BQ.1.2	H146-										
		1										
5	BQ.1.5	Y144-	E1144K									

		4	1									
1	BQ.1.10	Y144-										
		1										
3	BQ.1.13.1	G75A										
		2										
6	XBB.1	G184V										
		1										
52	XBB.1.5	Q14H	C15-	V16-	N17-	L18-	T19-	T20-	R21-	T22-	Q23-	S98F
		1	1	1	1	1	1	1	1	1	1	1
		V127F	H146K	Y200C	D215H	L244S	S255F	G257S	A263T	I434T	A475V	P491S
		4	6	1	2	1	1	2	1	1	1	1
		Y660H	A688V	S691Y	N703S	K776R	T1009I	W1102L				
		1	1	3	1	2	1	1				
4	XBB.1.5.12	T323I										
		4										
1	XBB.1.5.14	S221L	G1219C									
		1	1									
1	FD.1	L5F										
		1										
4	XBB.1.5.18	P621S										
		1										
11	EU.1.1	Q14K	H146K	T470N	V1068F							
		1	2	1	1							
6	XBB.1.5.28	H146K	T573I									
		2	6									
2	XBB.1.5.33	D427Y										
		2										
1	XBB.1.5.35	D198G										
		1										
2	XBB.1.5.37	G257S										
		1										

4	GV.1	S12F	Q607H	A623V	G1246V							
		1	1	1	1							
3	XBB.1.5.49	K182E	T478R									
		1	1									
12	XBB.1.5.52	S155R	G496S									
		1	3									
5	XBB.1.5.55	G257S	D627Y	I742V	P1162L							
		1	1	1	1							
2	XBB.1.5.63	Y145H										
		2										
1	GK.1.1.1	A352S	S494P									
		1	1									
6	JD.1.1	R158G										
		1										
1	JD.1.2	P251S										
		1										
63	XBB.1.9.1	G181V	G257S	T284I	A344V	V367F	T478N	P561S	L517F	L518P	Q675H	S689N
		1	2	2	1	1	5	1	1	1	25	1
		A706V	L752F	T883I	D950Y	T1009I	P1162S	V1264L				
		1	1	5	1	1	2	5				
17	FL.1.5.1	M153I										
		1										
6	HN.5	I332V	S704L	K1086R	V1228L							
		1	5	5	1							
4	KC.1	P82H	L249V	L1224F								
		1	1	1								
1	FL.2	T478R										
		1										
19	FL.3.1	H146K	G257S	A348S	G476S	G482R	S494P	Q675H	G1267E			
		1	2	2	1	1	1	4	2			
2	FL.5	D80G	G257S	W258C								

		1	1	1			
2	FL.13	H146K	F157L	A694V			
		1	1	1			
6	FL.15	H146P	R1014K	L1224F			
		1	1	1			
4	FL.15.1.1	I105F	G1219V	L1224F			
		1	1	1			
1	FL.17	V213G					
		1					
3	FL.18	V16F	L18S				
		1	1				
1	FL.25	T478R					
		1					
1	FL.26	Q836L					
		1					
1	FL.40	T478K					
		1					
1	XBB.1.9.2	G257S	W258C	A688V			
		1	1	1			
26	EG.1	G232C	G257S	D253Y	G257S	W258C	
		1	2	1	1	1	
2	EG.1.2	L5F					
		1					
3	EG.1.6	G1124V					
		2					
1	EG.5	N481K					
		1					
3	EG.5.1	H146K	L455F	L699I			
		1	1	1			
17	EG.5.1.1	L242-	A243-	W258R	L452R	L1224F	C1247F
		1	1	1	2	1	5

1	HK.2	G184V							
		1							
24	HK.3	E154K	M177I	F486L	Q675K	D936Y	D1084G	Y1110H	I1227V
		1	1	1	1	1	1	3	2
2	HK.3.1	A1078S							
		1							
2	HK.3.2	L1224F							
		1							
1	HK.3.9	E654K							
		1							
14	HK.6	P209L	G446I	L452R	V1104L				
		1	1	5	1				
18	HK.15	D138-	P139-	F140-	L141-	G142-	V143-	Y145-	
		1	1	1	1	1	1	1	
4	HK.26	I101T	P631T	L1224F					
		3	1	2					
11	EG.5.1.3	S256L	S371-	A372-	S373-	S375A	T376P	L455F	D936N
		2	1	1	1	1	1	1	1
1	JG.2	A262S							
		1							
14	JG.3	Q14K	S98F	L242-	A243-	Q1113L			
		1	1	2	2	1			
12	EG.5.1.6	H146K							
		4							
19	HV.1	H146K	I197V	L452W	S810L	H1101Y	L1224F		
		1	1	1	1	1	1		
20	EG.6.1	I128V	W258C						
		6	1						
2	EG.10.1.1	P272S							
		1							
1	EG.14	L5F							

		1							
7	XBB.1.16	W152L	Q613H	A1078S					
		1	1	2					
11	XBB.1.16.1	T274N	W258C	W258C	A783V				
		1	1	1	2				
1	FU.5	E156Q	E554K						
		1	1						
3	GY.1	I197T	A372S	A852V					
		3	3	1					
3	XBB.1.16.6	ins185LRV	F186G	A668V					
		1	1	1					
1	JF.4	H146K							
		1							
14	XBB.1.16.11	G142-	Y145D	V143-	Y144-	K182N	R214H	S459F	E661D
		1	1	1	1	1	1	1	3
9	XBB.1.16.13	P621S							
		1							
4	HF.1	V130I	E554D	T747I	D1153Y				
		1	1	1	2				
2	XBB.1.16.15	L293F	A684V						
		1	1						
2	XBB.1.16.21	E1258D							
		1							
3	FE.1.1	H146K							
		1							
1	GW.4	G184S							
		1							
1	FY.5.4	C1243F							
		1							
1	XBB.1.24.1	A262S							
		1							

1	XBB.1.16.31	L1224F										
		1										
1	XBB.1.41.2	Q218H	P561S									
		1	1									
11	XBB.1.42	G257S	W258C	E309G								
		1	1	1								
1	XBB.2.3.2	S929T	D1084Y									
		1	1									
1	HH.2.1	T478R										
		1										
4	XBB.2.3.3	D427H										
		1										
2	GJ.4	A67V										
		2										
7	GZ.1	T19L	D215G									
		7	5									
58	GE.1	N30S	L54F	E96G	Q115-	D138-	P139-	F140-	L141-	G142-	V143-	Y145-
		2	1	1	1	1	1	1	1	1	1	1
		H146K	R158G	I197V	S494P	I670M	A846V	V952I	S937T	S1242N		
		2	1	1	1	1	1	3	1	1		
6	GE.1.5	G142N										
		1										
13	GS.4.1	R158S										
		5										
2	GS.4.1.1	F490P										
		1										
2	XBB.3.2	T19L	S514F	G798D	S939F							
		1	1	2	1							
1	XBF	D1153Y	C1243F									
		1	1									
3	XBF.7.1	T1231I										

		1
8	XBU	S494P
		1
1	XCH.1	P1162S
		1
2	XDD	D420N
		1

[a]The mutations shown in Table S2 were determined based on the prevalence of mutations per lineage available at CoV-Spectrum (http//cov-spectrum.org/)(date last accessed 10 July 2024) [81].

[b]Total number of sequences that were identified for each lineage in this dataset.

[c]For lineages that were identified in lower frequency in this dataset, CoV-Spectrum (http//cov-spectrum.org/)(date last accessed 10 July 2024) was used for the expected common/uncommon mutations [81].

[d]The mutations that were listed as below 75% prevalence in CoV-Spectrum http//cov-spectrum.org/)(date last accessed 10 July 2024), but were found in higher frequencies (above 75%) in this dataset for specific lineages were also indicated [81].

81. Chen, C.; Nadeau, S.; Yared, M.; Voinov, P.; Xie, N.; Roemer, C.; Stadler, T. CoV-Spectrum: analysis of globally shared SARS-CoV-2 data to identify and characterize new variants. *Bioinformatics* **2022**, *38*, 1735–1737, doi:10.1093/bioinformatics/btab856.
